# Spatial lipidomics of *Fasciola hepatica* using high-resolution AP-SMALDI Orbitrap MSI – a lipid tissue atlas

**DOI:** 10.3389/fcimb.2026.1934046

**Published:** 2026-10-22

**Authors:** Carolin M. Morawietz, Stefanie Gerbig, Simone Haeberlein, Bernhard Spengler

**Affiliations:** 1 Institute of Inorganic and Analytical Chemistry, Justus Liebig University, Giessen, Germany; 2 TransMIT GmbH, Giessen, Germany; 3 Institute of Parasitology, Justus Liebig University, Giessen, Germany

**Keywords:** AP-SMALDI, *Fasciola hepatica*, fascioliasis, imaging, LC-MS/MS, lipids, MSI, neglected tropical diseases (NTDs)

## Abstract

Parasitic worms, helminths, cause significant morbidity and high infection rates globally. To understand parasite physiology and as basis for intervention strategies, knowledge on the parasite lipidome is a key component. High-resolution atmospheric pressure scanning microprobe MALDI MS imaging (AP-SMALDI MSI) enables spatially resolved molecular-level insights into biological samples. We applied this technique to study the lipidome of the common liver fluke *Fasciola hepatica* which causes fascioliasis in humans and grazing animals across all inhabited continents. In particular, four of the most relevant worm organs in terms of parasite-host contact and reproduction (tegument, intestine, ovary, and testis) were examined. We extracted *m*/*z* signals for each organ from AP-SMALDI MSI data of worm sections and the native sample surface, annotated them to lipid species using a database and eventually assigned 73% (positive-ion mode) and 61% (negative-ion mode) of the previously annotated signals to (bulk) lipid species by comparison with complementary LC-MS/MS data. Data revealed differences in the lipid constitution of the organs. Among other findings, we observed (Hex)Cer species exclusively present in the tegument and intestine, PS species to vary in abundance between dorsal and ventral tegument side, and PI lipids to occur with saturated C-chains in the tegument and with unsaturated ones in the gonads. High-resolution MSI also allowed for differentiation between intestinal ducts and gastrodermis and resolved several molecular layers in/around the ovary tissue. The comprehensive dataset comprises reproducibly detected lipid species and their distributions across the worm and offers a spatial lipidomic resource for investigators of *F. hepatica* and related species.

## Introduction

Fascioliasis is a food-borne zoonosis and a neglected tropical disease (NTD) which is in large parts caused by the common liver fluke *Fasciola hepatica* (*F. hepatica*). The disease involves an acute stage attributable to the destruction of liver tissue on which immature parasites feed, and chronic disease caused by the mature, adult worms that parasitize the bile duct and feed on its epithelium. Globally omnipresent, fascioliasis poses a threat to grazing animals and affects, according to common estimations, 2.6−17 million humans (Infantes et al., 2023) with many more at risk of infection.

The only medically approved drug for human use to date is triclabendazole (TCBZ). Even though there is progress in the understanding of the drug’s mode of action (Olivares-Ferretti et al., 2023), uncovering drug operation mechanisms remains a challenging task. Yet, tackling it is critical for the targeted development of new drugs, which are urgently needed since TCBZ resistances are more frequently reported in humans (Branco et al., 2020; Morales et al., 2021).

Over the last decades, proteomics and transcriptomics have been used to decipher the parasite’s gene expression, even for individual organs (Cwiklinski and Dalton, 2018; Gramberg et al., 2024) but there is a lack of spatially resolved lipidome data, which would also be of high interest. Since it has become apparent that lipids play a role far beyond being cell membrane components in cell signaling, as diagnostic marker molecules, and energy storage (Hannun and Obeid, 2008; Nakamura et al., 2014; Drain et al., 2016; Yu et al., 2017) they must be considered in order to better understand worm biochemistry and from that, drug operation on a molecular level. Consequentially, new insights may also support finding novel drug targets, and eventually aid the development of new drugs. Moreover, lipids also take part in immune response highlighting their relevance in regards to parasite-host interaction (Hubler and Kennedy, 2016; Wiedemann et al., 2022). Most publications on the lipid composition and lipid metabolism of *F. hepatica* originate from the last century, where different lipid classes (PC, PE, PA, PS, PI, Lyso-species, DG, TG, SM, PE/PC-plasmalogens, cardiolipins (Oldenborg et al., 1975; Brouwers et al., 1996)) were identified only in bulk *F. hepatica.* A recent mass spectrometric study focused on the class of glycolipids in *F. hepatica* (Luh et al., 2024). However, a detailed elucidation of lipids in the glycerophospholipid mass range is to date lacking in the literature.

The 1–3 cm long, bilateral and leaf-shaped body of *F. hepatica* accommodates numerous different organs and tissue types. A schematic overview on the organs in *Fasciola hepatica* is shown in **Figure 1A** (Kazakova, 2024; Gramberg, 2025). Of particular interest is the parasite’s surface organ (tegument) and the intestinal system since both keep contact to the host, either directly or via ingestion of host material. Both epithelial tissues play a role in nutrient uptake and host defense and thereby ensure that the parasites can survive for several years in their host (Leiper, 1938; Dalton et al., 2004). To stay attached to the host tissue, the worm is equipped with two suckers on the ventral side and has spines on the tegument to cling to the host tissue. A close view on the worm tegument is given by electron microscopic images in **Figure 1B**.

**Figure 1:**
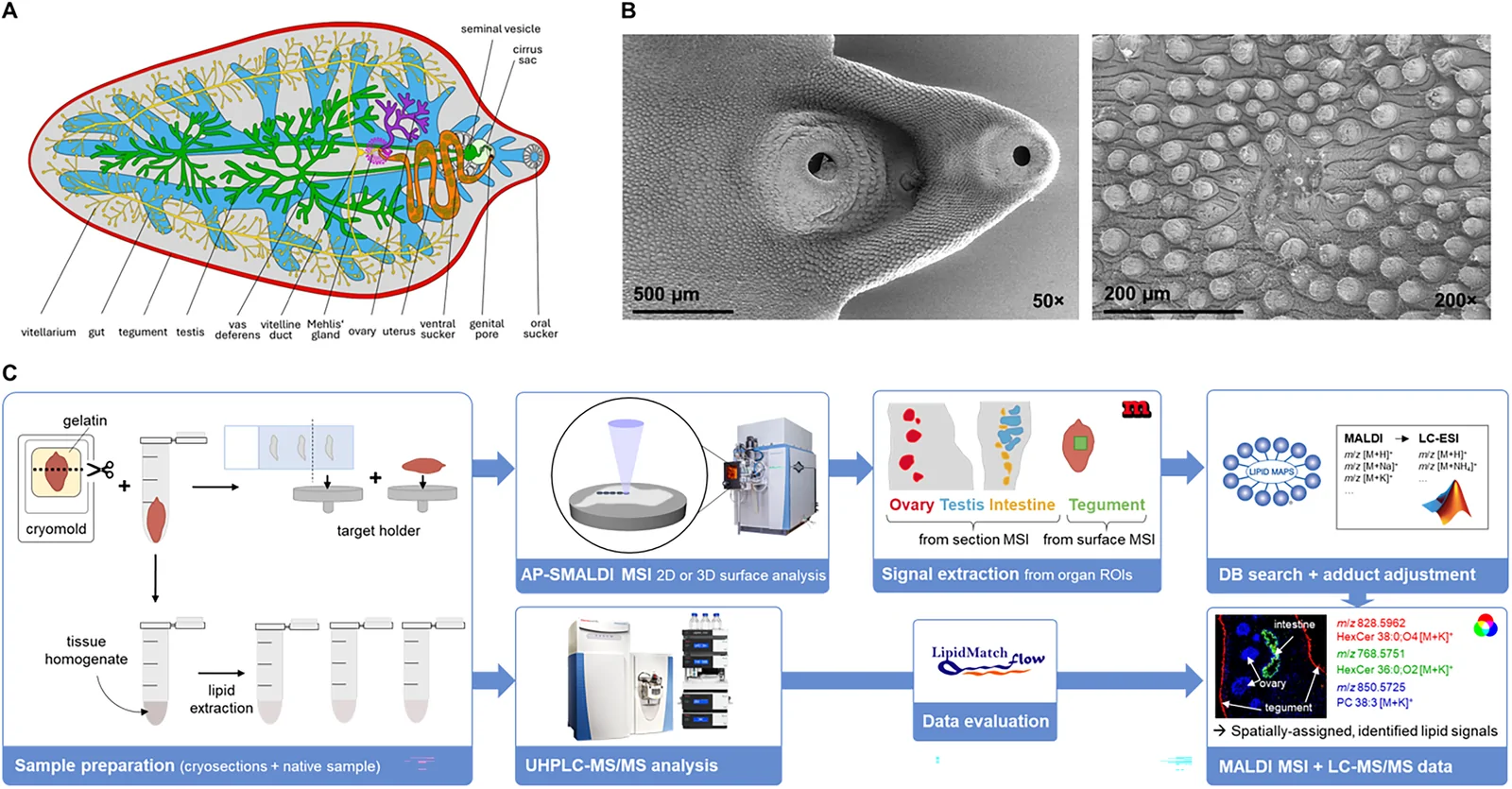
Anatomy of adult *F. hepatica* in the ventral view (Kazakova, Gramberg). The lipid atlas in this study covers tegument, gut, testis, and ovary. **(A)**, and electron microscopic images of the *Fasciola hepatica* tegument with spines **(B)**. General workflow starting with *Fasciola hepatica* worm sample that is either embedded and cut into sections, left native (both for fixation on a target holder for following APMALDI MSI analysis), or homogenized and its lipids extracted. Sections and native samples were subsequently analysed with AP-SMALDI MSI, and the obtained m/z signals were extracted from the individual organ tissue ROIs. Signals were annotated via a database search and then correlated with the information obtained from UHPLC-MS/MS analysis, considering differences in adduct formation. As a result, the annotated or (bulk)-identified lipid signals were spatially visualized in MS images from AP-SMALDI MSI analyses **(C)**.

Like most trematodes, *F. hepatica* is a hermaphrodite, developing both male (testis) and female gonads (vitellarium, ovary) in one individual. These allow for an astonishing reproductive capacity, culminating in the production of up to 50.000 eggs by a single worm per day (Happich and Boray, 1969). We rank tegument, intestinal system, ovary, and testis under the most relevant organs in terms of host contact and reproduction and followed the aim to create organ-specific lipidome information in this study.

Liquid Chromatography-Tandem Mass Spectrometry (LC-MS/MS) analysis can readily provide detailed information on the lipids overall present in the parasite. However, knowledge on the lipid distribution over the different worm tissues and organs is crucial for understanding their actual function. Matrix-assisted laser desorption/ionization (MALDI) mass spectrometry imaging (MSI) allows for label-free and spatially resolved analysis of biological material and is well suited for targeting analytes in a *F. hepatica*-sized sample, especially in the lipid mass range. Utilizing an Atmospheric-Pressure Scanning Microprobe MALDI (AP-SMALDI) (Spengler et al., 1994) MSI system coupled with an orbital trapping mass spectrometer, high resolution in space (down to 5 µm step size) and mass (usually 240,000 at *m*/*z* 200) can be achieved (Mokosch et al., 2021; Dreisbach et al., 2022). This generates highly informative datasets offering the spatial resolution needed for resolving all relevant organs in *F. hepatica* and the high mass resolution necessary for distinguishing between individual *m*/*z* signals, especially in the highly-populated mass range of *m*/*z* 650−900. Additionally, it provides a mass accuracy in the low-ppm range with which database search results for the obtained *m*/*z* signals become meaningful. The AP-SMALDI MSI system is equipped with an autofocus function, enabling pixel-wise autofocusing when using the 3D-surface imaging mode. Through this feature, non-flat or native samples, which can change in height due to moisture loss during the measurement, can be analyzed with constant laser spot diameters and thus fluence, guaranteeing comparative mass spectra behind each pixel.

Due to these benefits, we employed AP-SMALDI Orbitrap MSI to determine the lipids present in *F. hepatica* in a spatially-resolved manner by analyzing worm sections as well as the native parasite surface and complemented the obtained MSI information with LC-MS/MS data of bulk *F. hepatica*.

## Materials and methods

### Ethical statement

Animal experiments using rats (*Rattus norvegicus*) as model hosts were performed in accordance with Directive 2010/63/EU on the protection of animals used for scientific purposes and the German Animal Welfare Act. The experiments were approved by the Regional Council (Regierungspraesidium) Giessen (V54-19c20–15 h 02 GI 18/10 Nr. A16/2018). Parasites were obtained from a total of four rats, with a yield of 2–3 parasites per animal.

### Sample preparation

Chemicals used for the experiments are listed in **Supplementary Table 1**. with their purities and manufacturer information. The procedure of *F. hepatica* worm sampling and sample preparation was already described earlier (Morawietz et al., 2022) but is again outlined here: *F. hepatica*, freshly harvested from rats 20 weeks after infection, were temporarily stored in culture medium before they were successively washed in PBS and distilled water for one minute each. The worms were then put in a cryomold with 8wt% aqueous gelatin solution and immediately frozen on dry ice. In addition, some worms were frozen without embedding for native surface MSI and LC-MS/MS analysis. All samples were stored at −80 °C.

For AP-SMALDI MSI analysis, the gelatin block was fixated inside of a cryostat (HM525, Thermo Fisher Scientific) with *F. hepatica* in transversal orientation and the sample was cut into sections of 20 µm thickness at −23 °C. Section quality was controlled with a digital light microscope (VHX-5000, Keyence, Japan) and the samples were afterwards stored at −80 °C until further processing.

Before MSI analysis, sectioned samples were prepared in a similar way to previous studies (Morawietz et al., 2020; Mokosch et al., 2021; Morawietz et al., 2022): Sections were thawed in a desiccator for 20 min and then covered with matrix using the SMALDIPrep pneumatic sprayer system (TransMIT GmbH, Giessen, Germany). For positive-ion mode measurements, 80 µL of 2,5-dihydroxybenzoic acid (DHB, 30 g/L, in acetone/H_2_O, 1:1, *v*:*v*) spiked with 0.1vol% trifluoroacetic acid (TFA) was used, for negative-ion mode, 300 µL of 1,5-diaminonaphthalene (DAN, 3.5 g/L in MeOH/H_2_O, 9:1, *v*:*v*) was applied. A matrix flow rate of 10 µL/min and an N_2_ pressure of 1 bar was set in both cases.

Additionally, whole non-embedded worms were thawed, fixated on a MALDI target holder with a tiny drop of saturated gelatin solution (ventral worm side to the top), and dried in a desiccator at 100 mbar for 15 min. With the worm surface now dry enough for matrix application, the sample was covered with DHB (120 µL) or DAN (350 µL) matrix solutions of the above-mentioned concentrations using the SMALDIPrep device.

In preparation for LC-MS/MS analysis, lipids were extracted based on the MTBE protocol (Matyash et al., 2008; Breitkopf et al., 2017): Three 20-week-old *F. hepatica* worms were transversally parted into two halves and weighed. The front (anterior) parts (enriched in ovary tissue) and back (posterior) parts (enriched in testis tissue) were homogenized separately with a potter homogenizer on ice for 5 min. A calculated volume of NH_4_Ac buffer (50 mmol/L) was added for each worm-half resulting in a tissue concentration of 10 mg tissue/mL. For the following steps, Eppendorf tubes and pipette tips were pre-rinsed with the solvents they were intended for before use. All solvents were tempered to 5 °C before use.

150 µL of the diluted homogenates were transferred to Eppendorf tubes and 350 µL of MeOH was added. The mixtures were shaken for 1 min at 1500 rmp at 4 °C. After adding 1 mL of MTBE to each tube, the solutions were shaken again at 1000 rmp for 1 h (4 °C). Next, 300 µL of H_2_O were added, shaken again for 1 min at 1000 rpm and then centrifuged at 13,000 rmp for 8 min. The upper organic phases were taken off and stored on ice. The residues were re-extracted following the same procedure with half of the solvent volumes used previously. The combined organic phases of each sample were dried under an N_2_-stream on ice and then stored at −80 °C. Just before UHPLC-MS/MS analysis, samples were reconstituted with 200 µL ACN/isopropanol/H_2_O (65:30:5, *v*:*v*). All steps were parallelly executed with 100 µL NH_4_Ac buffer only (without sample) to use as an extraction blank.

### AP-SMALDI MSI analysis

*F. hepatica* sections originating from four biological replicates were used for MSI. From each individual worm, one transversal section from the anterior part (containing ovary tissue) and one transversal section from the posterior part (containing testis tissue) were analyzed in positive-ion mode. Consecutive sections of those measured in positive-ion mode were analyzed in negative-ion mode, resulting in four datasets per biological replicate and 16 measurements in total. Each measured section also contained tegument tissue and intestinal channels.

Analyses were performed using an AP-SMALDI^5^ AF ion source (Kompauer et al., 2017) (TransMIT GmbH, Giessen, Germany) coupled with an orbital trapping mass spectrometer (Q Exactive HF or Orbitrap Exploris 480, Thermo Fisher Scientific (Bremen) GmbH, Bremen, Germany). The ion source was operated with the SMALDIControl software (TransMIT GmbH, version 1.4) and the MS using the Tune software (Thermo Fisher Scientific, version 2.11 (QE HF), version 4.2 (OE 480)). Worm sections were measured in 2D-pixel mode and native worm surfaces in 3D-surface mode with pixel-wise autofocusing to keep the MALDI laser in focus with varying sample height due to moisture loss of the worm during the measurement. Pixel sizes were set to 7 or 8 µm with 50 laser pulses per pixel, the mass range to *m*/*z* 250−1000 and the mass resolving power to 240,000 (@*m*/*z* 200) in all cases. High voltages of 3.0 kV or −3.5 kV were applied.

Internal mass locking was executed scan-to-scan, utilizing a matrix mass (*m*/*z* 716.12462, corresponding to [5DHB − 4H_2_O + NH_4_]^+^) or Easy-IC (at OE 480 MS).

### On-tissue MS/MS

To complement the information from LC-MS/MS experiments, on-tissue MS/MS analyses were carried out on *F. hepatica* sections of several selected highly intense signals in positive- and negative-ion mode. The *m*/*z* isolation width was set to 1 and the HCD collision energy was adjusted individually for every precursor.

### H&E staining after AP-SMALDI MSI

To secure the correct identification of organs and tissue types, the matrix layers of the previously analyzed sections were removed with 80vol% aqueous EtOH and subsequently stained according to the standard hematoxylin and eosin (H&E) staining protocol. Optical images were recorded using a digital light microscope (VHX-5000, Keyence).

### LC-MS/MS analysis

LC separation was executed on a UHPLC system (Ultimate 3000 UPLC, Thermo Fisher Scientific) utilizing a reversed-phase C18 column (ACQUITY UPLC BEH C18 1.7 µm, 2.1×150 mm, Waters GmbH, Eschborn, Germany). Mobile phase A was constituted of H_2_O/ACN (40:60, *v*:*v*), mobile phase B of isopropanol/ACN/H_2_O (90:8:2, *v*:*v*). Ammonium formate (10 mmol/L) and 0.1 vol% formic acid were added to both phases. Column gradient parameters are listed in **Supplementary Table 2**. 20 µL of sample solution were injected and for each run a flow rate of 0.25 mL/min was set. Samples were measured in random order.

MS(/MS) data were acquired with an orbital trapping mass spectrometer (Q Exactive HF-X, Thermo Fisher Scientific). Ion source (HESI) parameters and MS(/MS) parameters can be found in **Supplementary Tables 3** and **4**. Exclusion lists for both ion modes were generated based on two MS^1^ extraction blank datasets each using MS-DIAL (Tsugawa et al., 2015) (version 5.1.230517). Parameters are listed in **Supplementary Table 5**. Inclusion lists resulted from previously acquired MALDI MSI data and contained the “reproducibly detected signals” (see Data analysis section) of each organ with the masses of the adducts expected in LC-MS/MS. Samples from the anterior part of the worm (ovary-enriched) were analyzed with the ovary-inclusion list, samples from the testis-enriched posterior part with the testis-inclusion list.

Lipid extracts of three biological replicates were analyzed in both, positive- and negative-ion mode (MS and ddMS^2^ scan). Additionally, pooled samples containing all three biological replicates, and a total of three extraction blanks were measured. No lipid standards were used during the analysis.

### Data analysis

Ion images were generated with the Mirion software package (Paschke et al., 2013) (TransMIT GmbH). In each dataset, a region of interest (ROI) was defined for the ovary, testis, and intestine tissue based on certain marker masses, i.e., *m*/*z* 850.5725 (ovary, positive), *m*/*z* 889.5830 (ovary, negative), *m*/*z* 713.4518 (testis, positive), *m*/*z* 913.5811 (testis, negative), *m*/*z* 822.5416 (intestine, positive), *m*/*z* 786.5298 (intestine, negative). Even though we did not observe smearing of the lipids when comparing optical and MS images, it should be noted that ROI selection based on MS signals did not meet highest anatomical precision and the ROIs might therefore include a low fraction of neighboring tissues.

Mass lists were created for each ROI fulfilling three criteria: 1. Minimum pixel coverage within the ROI of 5%, 2. Decimal places between 0.2 and 0.8 to focus on lipids and remove matrix signals, 3. Average signal intensity >150. Signals of the four biological replicates were then compared and signals that fulfilled the above-mentioned criteria in at least three biological replicates were considered “reproducibly detected” for the respective organ tissue.

Since the tegument is represented by much fewer pixels than the other organs in the section datasets, data from the native surface analyses (three biological replicates, 80×80 pixels each) were used for signal list generation, proceeding in the same way as described for the ROIs of the other organs. ^13^C isotopologue signals in the lists were manually identified based on mass difference (+1.003 Da between the isotopologues), signal intensity (in the investigated mass range the signal of the first ^13^C isotopologue is roughly 50%, and the second roughly 10% of the intensity of the pure ^12^C compound), and spatial distribution (isotopologues have the same spatial distribution in the MS image) relative to the base signal and were removed.

The resulting signal lists for each organ and ion mode were then loaded into the LIPIDMAPS database (Fahy et al., 2007) employing the *m*/*z*-based Lipid Maps Structure Database (LMSD) search. The parameters set for the search can be viewed in **Supplementary Table 6**. Commonly, the overlap reported between signals obtained with MALDI MS(I) and LC-(ESI) MS is, particularly in positive-ion mode, rather low (20−35%) (Vandenbosch et al., 2024; Rorsman et al., 2025). One reason for this is that different adduct ions are available and favored in these two techniques. In negative-ion mode, the effect is not as pronounced since in both techniques, [M−H]^−^ is the commonly formed ion. To increase the overlap between AP-SMALDI and LC-(ESI)-MS, we used an in-house-written tool in MATLAB (The MathWorks Inc., Natrick Massachusetts, version R2017a) to calculate the *m*/*z* values that are more likely to occur with LC-MS on the basis of the (MALDI MSI) adducts we chose in LIPIDMAPS (**Table 1**).

**Table 1:** Considered adduct ions for the two ionization techniques used.

Technique	Positive-ion mode	Negative-ion mode
MALDI MSI	[M+H]^+^, [M+H−H_2_O]^+^, [M+Na]^+^, [M+K]^+^, [M+2Na−H]^+^	[M−H]^−^, [M+Cl]^−^
LC-MS	[M+H]^+^, [M+H−H_2_O]^+^, [M+Na]^+^, [M+NH_4_]^+^	[M−H]^−^, [M+Cl]^−^, [M+HCO_2_]^−,^ [M−H−H_2_O] ^−^

The resulting mass lists were then also used as inclusion lists for the LC-MS/MS measurements.

LC-MS/MS data were evaluated with the automated, rule-based LipidMatch Flow 3.5 software (Koelmel et al., 2017).

Single-replicate and pooled MS^2^data were labeled as “ddMS^2^”, single-replicate MS data as “sample” and pooled MS data as “target”.

MS^1^ and MS^2^ *m*/*z* search tolerance was ±0.005 Da and ±5 ppm, respectively. Retention time was used for matching signals with MS/MS scans. Identifications were executed based on pooled sample data, however peak areas from the single replicate datasets were later on reported in the software output confirming the presence of the listed lipids in all datasets.

Next, the LipidMatch flow output files (Pos_MolecularSpecies and Neg_MolecularSpecies) were compared with the LIPIDMAPS signal annotations in the MATLAB tool output file, where the different adduct masses expected from “LC-MS adducts” were listed.

### Principle component analysis

For PCA plot generation, the datasets (as .raw files) were loaded into the SciLS Lab software (version 2024a Pro, Bruker Daltonics), and analyzed similarly to existing studies (Andersen et al., 2021; Frendi et al., 2024). ROIs were defined for each organ (ovary, intestine, testis, tegument) and a feature list with signals having decimal places between 0.2 und 0.8 was created (including the 1217 most intense signals in positive-ion mode and the 804 most intense signals in negative-ion mode). A PCA comparing the signals from the replicate-combined ovary-, testis-, intestine-, and tegument-ROIs was calculated (using TIC normalization, no denoising, 5 components, unit variance scaling, individual spectra) for both ion modes. The PCA uses individual pixels from at least two biological and technical replicates as independent observations.

## Results

### Identification of lipid class profiles for major organs in *F. hepatica*

Aiming to generate an organ-specific lipidome dataset for *F. hepatica*, we performed AP-SMALDI MSI on sections and the surface of worms, following the workflow summarized in **Figure 1C**. Signal lists were obtained for each selected organ – ovary, testis, intestine, and tegument – in positive- and negative-ion mode. Reproducibly detected signals (in total 377 in positive-ion mode and 150 in negative-ion mode) were then loaded into the LIPIDMAPS database yielding one or more annotations for on average 61% (positive-ion mode) and 77% (negative-ion mode) of the signals. For confirmation, we additionally performed LC-MS/MS analyses from bulk *F. hepatica* lipid extracts and compared the annotated signals from the previous AP-SMALDI MSI analyses to the lipid species found in the LC-MS/MS data output. Given that adduct formation may lead to different *m*/*z* values for the same compound in MALDI MS and LC-(ESI) MS, we compared the data based on calculated *m*/*z* values. As a result, on average 73% (positive-ion mode) and 61% (negative-ion mode) of the previously annotated, reproducibly detected signals were assigned to one or more (bulk) lipid species which were found with (LC-)MS/MS. For clear communication of signal identification confidence, we used the following levels. 1: Reproducibly detected signals without LIPIDMAPS database match, presumably not lipids. 2: Ambiguously database-annotated signal, no MS/MS information. 3: Unambiguous database-annotated signal, no MS/MS information. 4: Signals with (LC)-MS/MS match(es) resolving the bulk structure (lipid class, #carbons, saturation), 5: Signals with MS/MS match(es) with more than bulk structure resolved (typically individual FAs with respective saturation). It should be noted, that our data do not allow for deeper structural elucidation such as resolution of sn-position of the FAs or double bond positions.

The (Hex)Cer lipid species were assigned to confirmation level 4 or 5, even though they were rarely found in the LC-MS/MS output files. This is due to the fact that most of the Cer species were confirmed by manual LC-MS/MS spectra evaluation. An example is given in **Supplementary Figure 1**. In **Supplementary Sheet 1**, the diagnostic fragments used for (bulk) structure confirmation are listed in the “comments” column of the respective ceramide signal. The mass tolerance for the fragments was ±0.01 Da from the exact mass and a minimum of three diagnostic fragments (2x water-loss, at least one N^II^ fragment) were used for assignment to ceramide class. The more polar HexCer lipids were presumably hardly extracted with the MTBE method, however, they were typically the only database hits within reasonable mass deviation for the respective MALDI MSI signals. We see additional evidence when considering the spatial distribution: The signals assigned to (Hex)Cer species were exclusively present in the tegument and/or the intestinal channels while glycerophospholipids did not show this characteristic distribution.

PCA analysis of the signals demonstrated a separation of tegument, intestine, ovary, and testis in both ion modes, indicating that there are *m*/*z* signals that characterize the individual organs (**Figure 2**). The highest degree of individuality is observable for the tegument in positive-ion mode. In parts, but certainly not completely, this may originate from an overlap with pixels from the worm surrounding. The gonads (ovary and testis) occur in proximity to one another in both ion modes. Among the most distinctive features in positive-ion mode we found *m*/*z* 828.597 and *m*/*z* 796.627 (both tegument), *m*/*z* 822.541 and *m*/*z* 782.596 (both intestine), and *m*/*z* 870.587 and *m*/*z* 697.478 (both testis). In negative-ion mode, *m*/*z* 631.470 and *m*/*z* 762.529 (both tegument), *m*/*z* 788.543 and *m*/*z* 824.561 (both intestine), and *m*/*z* 929.656 and *m*/*z* 742.537 (both ovary) were most distinctive. The PCA loading plots can be viewed in **Supplementary Figure 2** and a csv-file with the loadings coordinates is provided as a **Supplementary File**.

**Figure 2:**
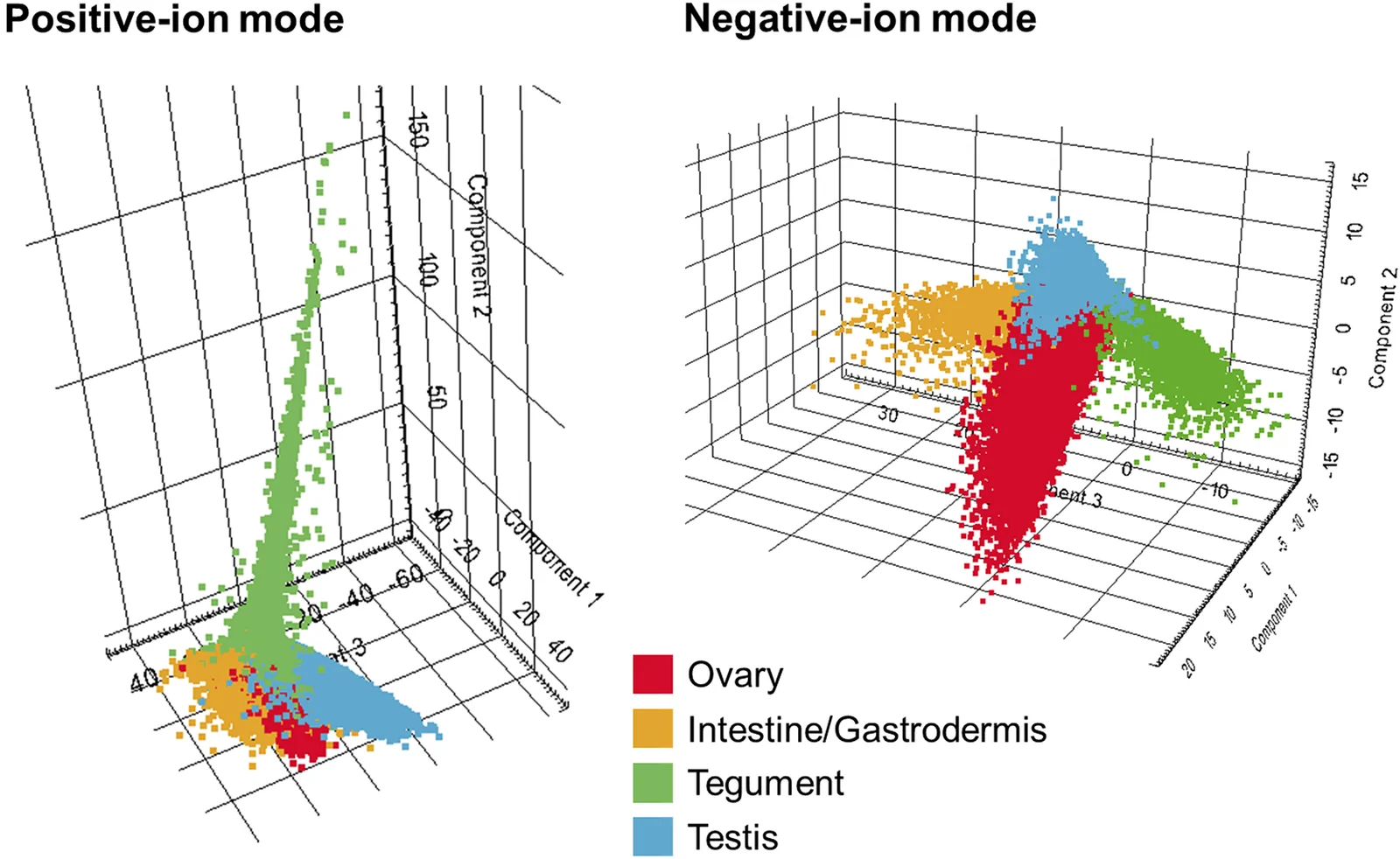
Principal component analysis (PCA) plots based on signals in the range *m*/*z* 250−1000 with decimal places between 0.2 and 0.8. The four organs of interest (ovary, intestine, tegument, and testis) were selected as ROIs and compared based on their *m*/*z* signal composition using PCA . Data were obtained from 2−4 different *F. hepatica* specimens.

A list showing all reproducibly detected signals, their *m*/*z* values in MALDI MSI and, if applicable, LC-MS(/MS), the deviations from the putative exact masses, the lipid assignments, sum formulae, adducts, confidence levels of identification, and the locations in *F. hepatica* is given in **Supplementary Sheet 1** (positive-ion mode) and **Supplementary Sheet 2** (negative-ion mode). In positive-ion mode, species annotated as PA lipids by LIPIDMAPS presumably originated from in-source fragmentation in MALDI and were therefore also searched for as the corresponding PC or PE in the LC-MS/MS data and, if found, assigned as such. More detailed structural information from the lipidmatch flow output, if existent, is given in parentheses behind the bulk lipid species in the list. Information coming from on-tissue MS/MS experiments is noted under “comments”. In **Supplementary Images 1** (positive-ion mode) and **Supplementary Images 2** (negative-ion mode), the spatial distributions of all the reproducibly detected signals can be viewed for a section from the anterior and posterior worm part. The pixel brightness of the images was individually adjusted for every image in order to clearly visualize the signal distribution. Therefore, pixel brightness must not be linked to signal intensity when comparing images against each other.

In **Figure 3**, the lipid class profiles of the worm organs are depicted. Especially in positive-ion mode, the tegument stood out through its high ceramide content ((Hex)Cer and ICP species) which was also observed in the intestine to a lower extent in both ion modes. Furthermore, we observed the highest fraction of lysated lipids in the tegument. The lipid profiles of the gonads were very similar in positive-ion mode. In negative-ion mode, the testis tissue shows a particularly high PI lipid content, while the ovary is richer in (mono-/di-methylated) PE and PC lipid species. It should be noted that a more detailed comparison on a low-percentile level may not be reasonable because the charts are based on qualitative data and do not necessarily refer to organ-specific signals.

**Figure 3:**
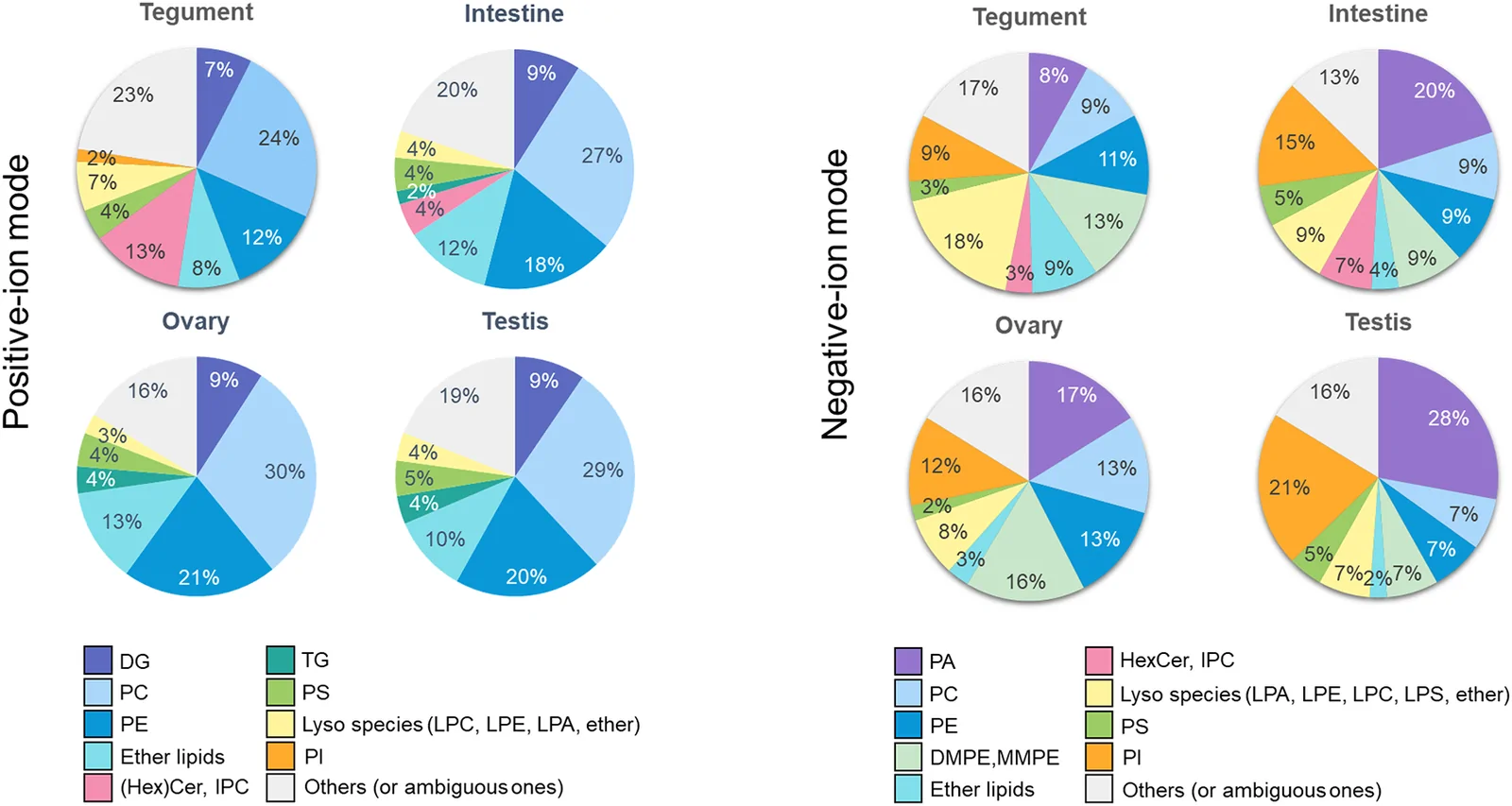
Lipid class profiles of the four organs of interest in positive- and negative-ion mode. Pie charts are based on the reproducibly detected signals detected for each organ and do not reflect organ-specific signals. The fractions should not be interpreted on a single-percentile-accuracy level; they rather show major trends since they are based on qualitative data. All signals annotated via LC-MS/MS and/or unambiguously annotated in LIPIDMAPS were included. Lipid abbreviations: DG, diacylglyceride; PC, phosphatidylcholine; PE, phosphatidylethanolamine; (Hex)Cer, (hexosyl)ceramide; IPC, inositolphosphorylceramide; TG, triacylglyceride; PS, phosphatidylserine; PI, phosphatidylinositol; PA, phosphatidic acid; M/DMPE, mono/dimethylphosphatidylethanolamine.

### Dorso-ventral differences in the lipid composition of the tegument

We obtained MALDI MSI data of the liver fluke tegument from two different approaches: 3D-surface imaging and imaging of transversal sections of worms. Most high-quality spectral data was generated by 3D-surface analysis from the native worm surface, an approach that allowed a more precise visualization of the tegument. This becomes visible when taking a look at the MALDI MS images of all reproducibly detected signals in the **Supplementary Sheets 1 and 2**. Signals detected on the native *F. hepatica* surface often occur localized to the tegument but only with low intensity and pixel coverage in the section measurements. **Figure 4** shows one measurement in each ion mode consisting of 80×80 pixels (7 µm pixel size) from an area on the ventral worm surface. In positive-ion mode, a structure is visible correlating with the location of the parasite’s spines, which are tiny scale-like hooks on the worm tegument aiding the attachment to the host tissue. However, this is most likely originating from the way how DHB matrix crystallizes on the uneven surface, because we observe this pattern also in the Total Ion Count (TIC) image of the positive-ion mode measurements and not in negative-on mode, where a different matrix was used.

**Figure 4:**
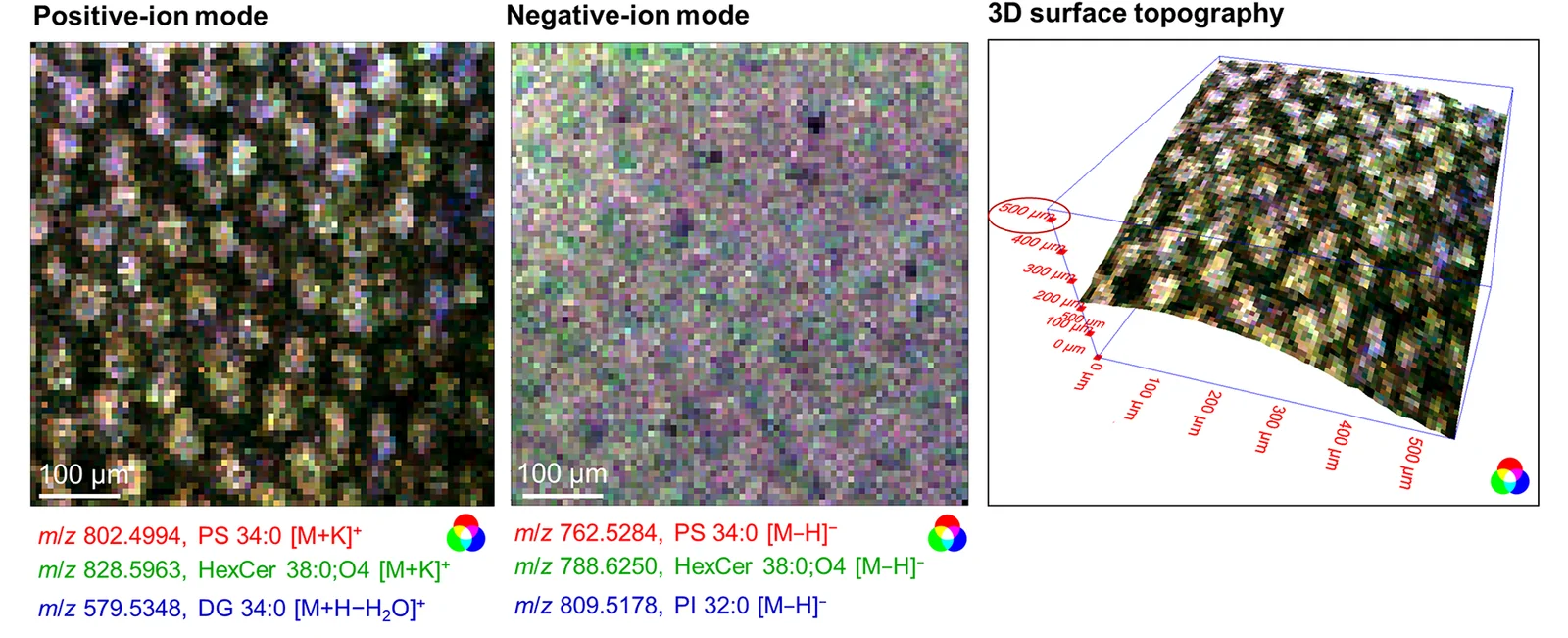
AP-SMALDI MS RGB images acquired in 3D-surface mode, analysing a 80×80 pixel area on the native worm surface in positive- and negative ion mode. Pixel size in both images is 7 µm. The 3D topography image of the positive-ion mode measurement shows sample height variations of 500 µm during the course of the measurement.

The native sample lost humidity in the course of the analysis causing changes in sample height. Through the pixel-wise autofocusing integrated in the AP-SMALDI^5^ AF ion source a meaningful analysis of the native surface was enabled. We noticed a lowering of the sample in z direction of around 500 µm within the 4 h-measurement (**Figure 4**). Without adjusting the MALDI laser focus plane to the changing sample height, the sample would have gone out of focus resulting in signal intensity decrease to the point of total signal loss.

Among the lipids predominantly present in the tegumental region, we noticed some distinctive features which are summarized in **Figures 5A–F**. We found lipid species that are primarily present in the tegument on the ventral worm side. One example is PS 34:0, which was detected in moderate intensity in positive-ion mode (**Figure 5B**), and very intensely in negative-ion mode. The lipid is further present in the sub-tegumental layer of cytons that are connected to the syncytial surface (**Figure 5C**). This stands in contrast to, for example, the tegument-specific (Hex)Cer lipids which are much more evenly distributed between the ventral and dorsal side (**Figures 5B, C, F**). Besides PS 34:0, we found also other (mostly saturated PS-) lipids with increased intensity on the ventral side (PS 32:0, PS 33:0, PS 36:3, Am-Hex-PE 36:5). When comparing ROIs of the ventral and dorsal sides in a PCA, no clear separation was observed (data not shown), presumably because it is a quantitative, and not a qualitative difference of only few *m*/*z* signals. Nevertheless, the above-mentioned species do clearly vary in abundance between ventral and dorsal side consistently through technical and biological replicates. When comparing the average intensities of the mentioned signals between the dorsal and ventral ROIs, we observed a 2–9 times higher intensity on the ventral tegument side.

**Figure 5:**
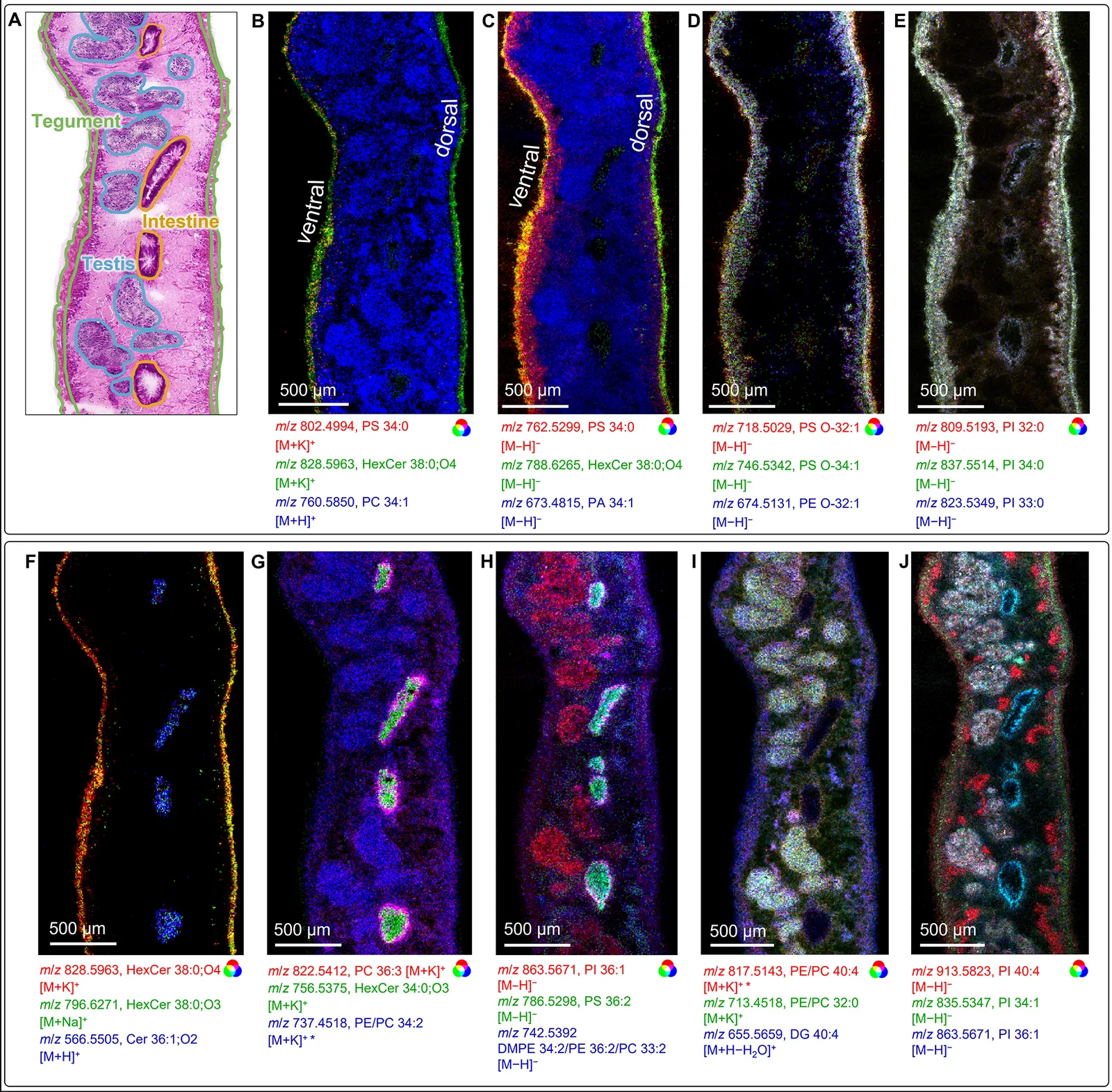
Optical image of H&E-stained section (from posterior worm part) which was previously analysed in positive-ion mode **(A)**, AP-SMALDI MS RGB-image in positive-ion mode **(B)**, AP-SMALDI MS RGB-image of a consecutive section in negative-ion mode **(C)**. Both highlight the hexosylceramide species exclusively occurring in the tegument (green colour channel) and the PS 34:0 species that is predominantly detected on the ventral worm side. AP-SMALDI RGB negative-ion mode images in **(D)** and **(E)** visualize the vastly co-localized ether- and saturated PI lipid species in the tegument and the sub-tegumental cyton layer. AP-SMALDI MS RGB-image in (F) showcases the tegumental and intestinal distribution of (Hex)Cer lipid species in positive-ion mode. AP-SMALDI MS RGB-images in **(G)** and **(H)** highlight signals in the intestine, revealing a substructure that enables differentiation between intestinal lumen and gastrodermis in both ion modes. Signals from both ion modes accumulating in the testis tissue are overlayed in AP-SMALDI MS RGB-images in **(I)** and **(J)** Pixel size in all images is 7 µm.

We also noticed that some signals occurred enriched at the ventral side of the parenchyma, too (e.g., *m*/*z* 745.2373, *m*/*z* 833.2531, *m*/*z* 851.2636, *m*/*z* 995.3056, all positive-ion mode). However, these signals did not have any matching molecular species in LIPIDMAPS and are therefore likely not lipids.

Another observation we made is the presence and strong co-localization of several ether-linked lipid species (plasmalogens and plasmanyls) in the tegument, such as PS O-32:1, PS O-34:1, and PE O-32:1 (**Figure 5D**). A similarly strong spatial correlation can be noticed among the saturated PI lipids PI 32:0, PI 33:0, PI 34:0 in the tegument and sub-tegumental cyton layer (**Figure 5E**).

### Intestinal lumen and gastrodermis are discriminated by distinct lipid species

As mentioned earlier, both organs with epithelial tissues - the intestine and the tegument - contain a high number of (Hex)Cer species. We found a tendency that the hydroxylation degree of these compounds is higher in the tegument than it is in the intestine, which is shown for Cer 36:1;O2 (intestine only), HexCer 38:0;O3 (present in both, intestine and tegument), and HexCer 38:0;O4 (tegument only) in **Figure 5F**. In the images of both ion modes, the pixel size of 7 µm allowed for resolving a sub-structure in the intestine area (**Figures 5G, H**). Some compounds were detected inside the intestinal lumen while others were present in the gastrodermis, delimiting the lumen from the surrounding tissue. We observed that the area corresponding to the lumen exclusively contained (Hex)Cer, IPC, and PS species and the gastrodermis consisted of PC(-O), PE(-O), DMPE, PA, and PI species, as well as the DGs of the same C-chain lengths (e.g., PC-O 36:2, PE 36:2, DMPE 36:2, PA 36:2, PI 36:2, and DG 36:2, but PS 36:2 in intestinal cannel).

### Shared and unique lipid species in different reproductive organs

The adult, mature stage *F. hepatica* has both male (testis) and female (ovary and vitellarium) reproductive organs. While the ovary is a more compact organ located in the anterior half of the body, the vitellarium and testis are follicular and spread through a large part of the body (**Figure 1**). The vitellarium, or yolk gland, produces vitelline cells that are major component of the composite eggs of flatworms (Hanna et al., 2021). All organs can readily be discriminated in H&E-stained tissue sections (**Figures 5A**, **6A**). Our MALDI MSI data revealed that the testis tissue is rich in unsaturated (DM)PE, PC, PI, and corresponding DG species (**Figures 5I, J**), which is also the case for the ovary tissue. Examples present in both testis and ovary tissue are PC 40:4 and PC 40:5, with their corresponding DGs, PC 42:6 (positive-ion mode), and PI O-34:2 (mainly around the testis tissue), PI 38:2, and PI 40:6 (negative-ion mode). The high number of PI lipid species detected in negative-ion mode (**Figure 5J**) was already indicated in **Figure 3**. The PIs found in the testis and ovary tissue were all unsaturated, in contrast to the saturated ones detected in the tegument.

**Figure 6:**
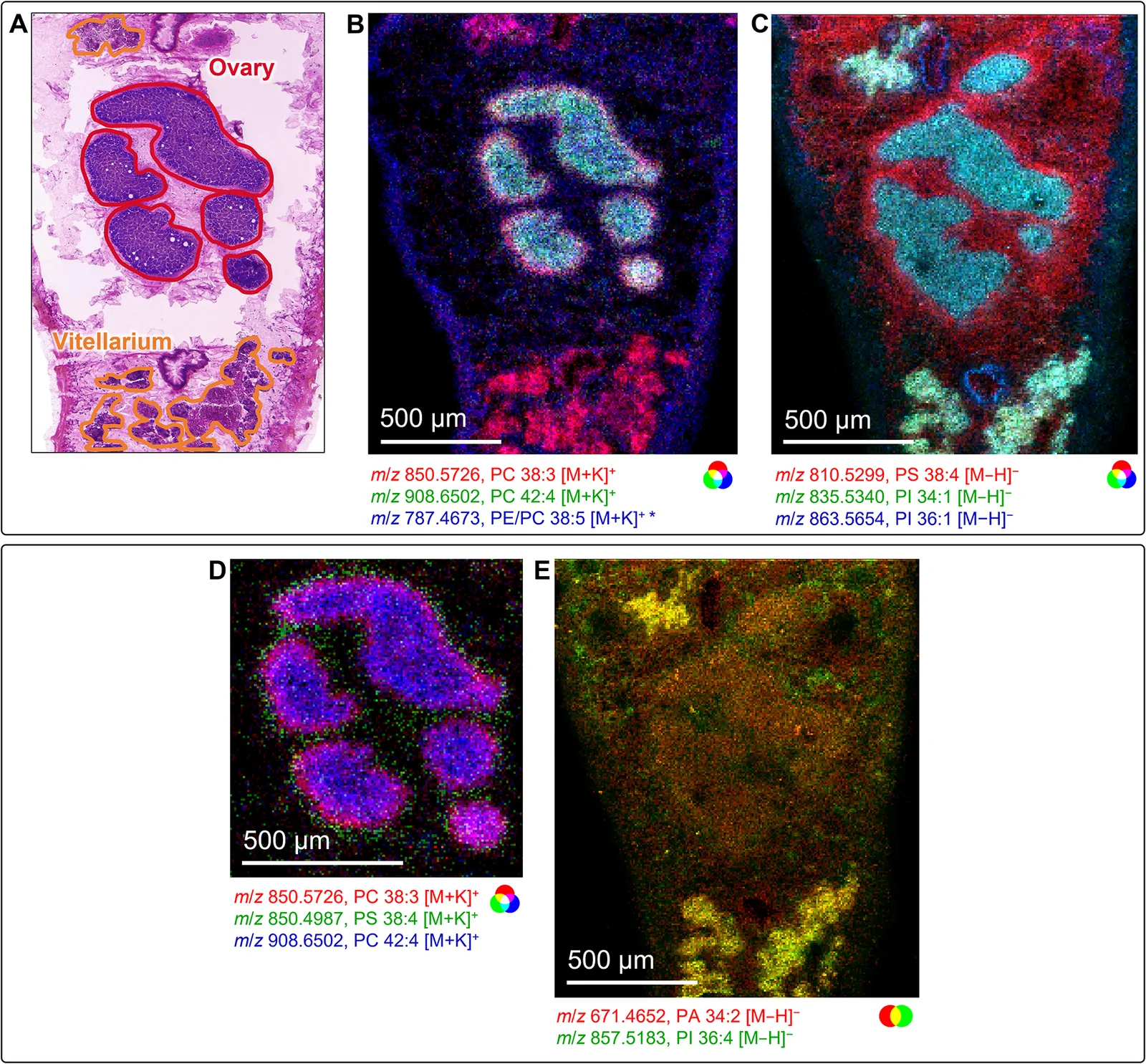
Optical image of H&E-stained section (from anterior worm part) which was previously analysed in positive-ion mode **(A)**, AP-SMALDI MS RGB-image in positive-ion mode visualizing PC and PE species co-localized in the ovary **(B)**. In negative-ion mode, PI species detected in the ovary and vitellarium, and PS 38:4, accumulating in a layer surrounding the ovary layer are shown **(C)**. AP-SMALDI MS image in (D) reveals a three-layered substructure via an RGB-overlay of species from **(B)** and **(C)**. In E, an AP-SMALDI MS RG-image of two lipid species strongly accumulating in the vitellarium is presented in negative-ion mode. Pixel size in all images is 7 µm.

Evaluation of the anterior worm part revealed various lipid species that accumulated in the ovary, particularly PC 42:4, which was exclusively detected in this tissue type. PC 38:3 was, in addition to the ovary, also detected in the gastrodermis and the vitellarium. PC 38:3 tends to be more abundant in the outer margin of the ovary (**Figure 6B**). In negative-ion mode, we found unsaturated PI species occurring in ovary and vitellarium, and another unsaturated PS, PS 38:4, accumulated around the ovary (**Figure 6C**). The latter in low intensity was also detected in positive-ion mode, allowing for an RGB overlay of the above-mentioned species for exact comparison of their location (**Figure 6D**): PC 38:3, PS 38:4, and PC 42:4 shape a three-layered sub-structure in and around the ovary tissue. We also found PS 40:4 and PS 41:3 spatially superimposed with PS 38:4 in the outmost layer (data not shown). Two other lipid species, PA 34:2 and PI 36:4, were scarcely detected in the ovary and parenchyma but are noticeably accumulated in the vitellarium (**Figure 6E**).

### Other noticeable observations and distributions

Our MALDI MSI and LC-MS/MS data show the presence of lipid species containing odd-numbered C-chains in *F. hepatica*. Odd-chained FA-lipids are not naturally produced in humans and most other animals, except for ruminants (Vlaeminck et al., 2006). Among the odd-chain FA compounds found in *F. hepatica* were PC 31:0, PC 33:0, PE 35:1, and PS 43:4, the shorter-chained ones presumably built from a C_15_ or C_17_ fatty acyl (FA) component. When looking at FA components in general, we also observed some that rank among the “very long-chain” FAs. Among the reproducibly detected lipids, C_22_ (e.g., in PC 42:6 or PS 40:4 in **Supplementary Sheet 1**) and C_24_ (in PC 42:2 in **Supplementary Sheet 1**) were found and, beyond that, LC-MS/MS data (not shown) also suggest C_26_, C_28_, C_30_, and C_32_ components present in *F. hepatica*.

Images from the anterior and the posterior worm sections suggest that all identified or annotated triglycerides (TGs) have the same spatial distribution in the parenchymatic tissue, exemplarily shown for TG 56:6, TG 54:6, and TG 58:10 in **Figure 7A**. In addition, glycerophospholipids of various classes, chain lengths, and saturation degrees were detected in a spatial pattern indicated with arrows in **Figures 7B, C**. It remains unclear which tissue structures relate to these signals. Excretory ducts may be one possibility since they were found to contain lipid vesicles within the duct lumen (Burren et al., 1967).

**Figure 7:**
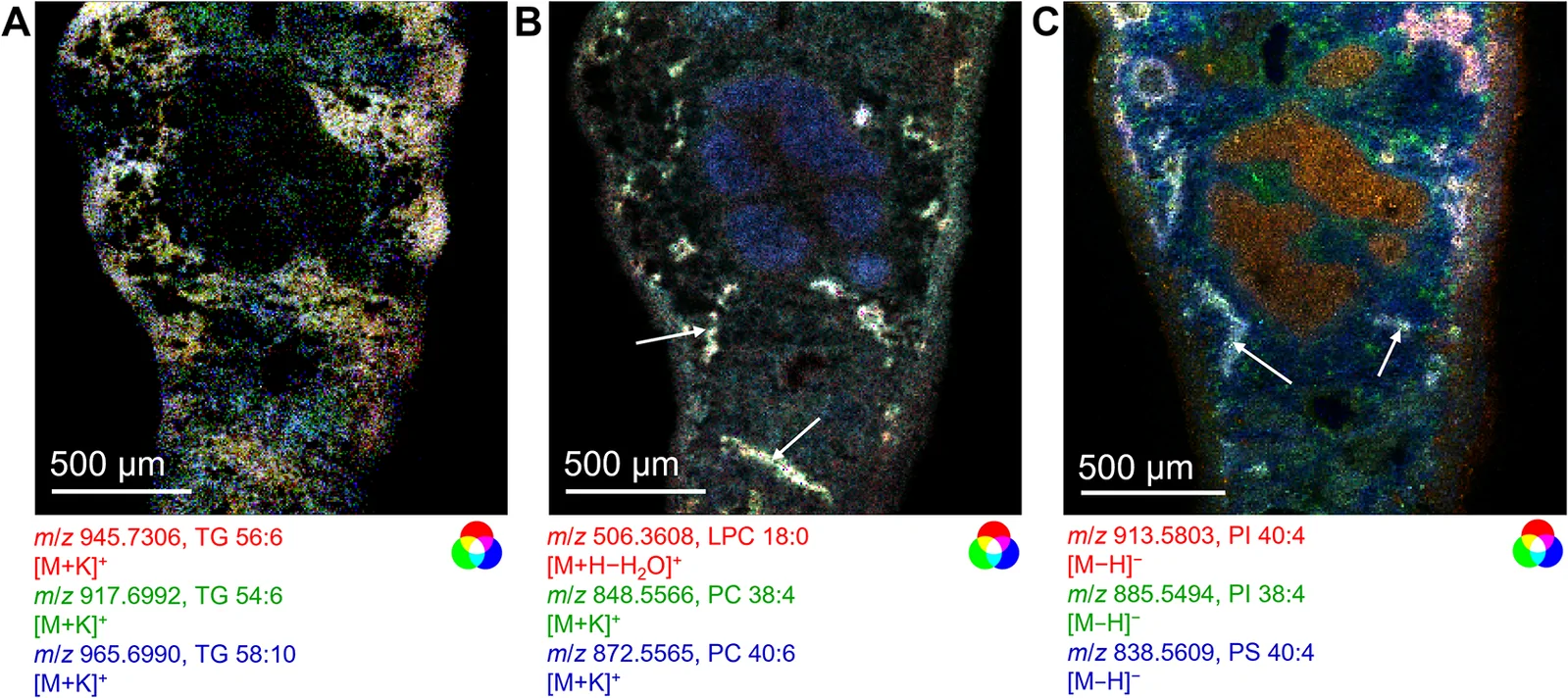
AP-SMALDI MS RGB-image in positive-ion mode visualizing exemplary triglyceride (TG) species that are vastly co-localized **(A)**. AP-SMALDI MS RGB-images in **(B)** and **(C)** (positive- and negative-ion mode) highlight lipid species which were detected most likely in the presumed excretory cells of *F. hepatica*, indicated by the white arrows. All images can be compared to the optically imaged area in **Figure 6A**. Pixel size in all images is 7 µm.

## Discussion

In this study, we aimed to advance the understanding of *F. hepatica* physiology by generating a comprehensive spatial lipidomic atlas of the parasite. Using MALDI MSI, we characterized lipid distributions across the tegument, intestine, testis, and ovary, and assigned a substantial proportion of the detected lipid signals by integrating MALDI MSI data with complementary LC-MS/MS analyses.

### Ion adduct adjustment leads to a high fraction of identified lipid signals

From the analytical point of view, our experiments yielded an above-average (Vandenbosch et al., 2024; Rorsman et al., 2025) signal bulk structure-identification rate. On average, 73% (positive-ion mode) and 61% (negative-ion mode) of the previously database-annotated signals were assigned to one or more bulk lipid species that were found with LC-MS/MS. This is due to the fact that we took the differences in ion adduct formation into account which are observed between the two different ionization methods MALDI and ESI. For example, in positive-ion mode, TGs are detected as [M+K]^+^ in MALDI but as [M+NH_4_]^+^ in LC-ESI. DGs behave the same in LC-ESI but form [M+H-H_2_O]^+^ in MALDI. PC/PE/PI/PS species are detected as [M+H]^+^ in LC-ESI but tend to be most intense as [M+Na]^+^ or [M+K]^+^ in MALDI. In negative-ion mode, L(PC)s occur as [M−H]^−^ in MALDI but as [M+HCO_2_]^−^ in LC-ESI. These differences lead to different *m*/*z* values for the same analyte molecule and, if not considered, strongly lower the molecular species match between MALDI and LC-ESI.

### Lipids in the tegument can be allocated to enzymes

The tegument of parasitic flatworms is a specialized, syncytial epithelium overlying a network of body wall muscles and sunken cytons, allowing for efficient nutrient absorption and immune evasion (Tyler and Hooge, 2004).

It is reasonable that among the various organs of a parasite like *F. hepatica*, the tegument and the intestine are of highest importance for parasite survival since they are directly exposed to the host and its immune system (Dalton et al., 2004). The results of our PCA based on spatially-assigned mass spectrometric signals confirmed this exceptional role when examining *m*/*z* signals in the lipid mass range. Especially the tegument seems comprised of a unique lipid composition. Our data suggest that in comparison with the other organs investigated, lipids of the categories (Hex)Cer and IPC as well as lyso- and ether-linked glycerophospholipids are often specific to or accumulated in the tegument and/or its sub-layers. Here, the relation to the intestine as the other epithelial parasite-host-contact area is noticeable, since some of the tegument lipids, or closely related species, were also found there. The literature reports sphingomyelinases in tegument and gastrodermis and a lysophospholipid-regulating Group XV phospholipase A2 in the tegument (Cameron et al., 2017; Bennett et al., 2022), which may be involved in the synthesis of mentioned lipid species. The observation that glycerophospholipids are also found in the tegument is in line with the previously described detection of phospholipid transporting ATP-ase at the same location (Morales and Espino, 2012). Thus, our organ-resolved lipidome data help to allocate biomolecules to enzymes involved in lipid synthesis.

### Possible roles of ceramides in the epithelial tissue

The presence of hydroxylated glycosphingolipids in parasitic teguments has been reported earlier in cestodes (Singh et al., 1987). In this context, it was proposed that the capacity of hydroxylated ceramide moieties for intermolecular hydrogen bonding among themselves and with phosphoglycerides aids plasma membrane stabilization, even more so when the species are saturated. Glycoceramides in the tegument surface may therefore be relevant for maintaining a strong barrier function (Singh et al., 1987). Hexosylceramides were also reported in *Fasciola* spp. homogenates by Wuhrer et al., who also located globotriaosylceramide (CD77) species to the tegument by immunohistochemistry. These gangliosides are presumed to induce damage to host lymphocytes (Wuhrer et al., 2001), suggesting that tegumental glycoceramides might also have a signaling function in parasite-host interaction. Higher-mass species closely related to the (Hex)Cers detected in this study were recently demonstrated by our group to be present in the *F. hepatica* tegument and intestine using AP-SMALDI MSI (Luh et al., 2024). In the *F. hepatica*-related helminth *S. mansoni*, sphingomyelins, which can break down into ceramides, were reported enriched in the tegument compared to the inner worm (Kadesch et al., 2020). Even though we hardly found any sphingomyelins in our data, we noticed the presence of sphingolipids in the tegument as a parallel.

Also, inositolphosphoryl ceramides (ICPs) were found to be tegument-enriched in our study. ICPs are primarily known from plants and fungi but were also found in protozoan parasites like *Leishmania*, where they, together with other ceramides, are suggested to have protective functions shielding the parasite membrane from sterol-specific toxins (Levatti et al., 2017; Haram et al., 2023).

### Possible functions of lysated and ether-linked glycerophospholipids in the worm tegument

A high fraction of lysated lipids has already been reported in the tegument of *S. mansoni*, a related trematode (Retra et al., 2015; Kadesch et al., 2020). In this parasite, lipid mediators including lyso-species are released from the tegument. LPC and LPS species have a signaling role via Toll-like receptor 2 (TLR2) which, in the case of LPC, mediates eosinophil recruitment and function that can support fibrosis and tissue repair and may therefore contribute to the pathophysiology of chronic schistosome infection (Magalhaes et al., 2010). It was further shown that LPC and LPS activate TLR2 on host immune cells, inducing a down-regulation of host immune response (van der Kleij et al., 2002; Retra et al., 2008; Magalhaes et al., 2019). Similar effects have been reported on macrophages (Assunçao et al., 2017). Regarding *F. hepatica* specifically, the literature does not report any information on lysated lipids. The accumulation in the tegument of *F. hepatica* found in the present study may point to a similar function of lyso lipid species as observed in *S. mansoni*.

Ether-linked glycerophospholipids have already been detected in lipid extracts of homogenized *F. hepatica (*Oldenborg et al., 1975). However, their location inside the worm and their function have not yet been elucidated. In general, the chemical structure of ether-linked glycerophospholipids allows for stronger intermolecular hydrogen-bonding between the lipid headgroups compared to diacyl phospholipids, which has influence on membrane structure and thus can affect membrane fusion or the activity of membrane-bound proteins (Lohner, 1996). In line with our findings, it is known from *S. mansoni*, that ether lipids are enriched in the tegument where they might contribute to a reduction of host immune response (Brouwers et al., 1998; Retra et al., 2015). It is also known that they can shield against reactive oxygen species (Zoeller et al., 2002) that are released by host immune cells.

### Phosphatidylserine lipids might indicate localized tissue function and cell types

The main two observations regarding PS lipids in the present study are the specific occurrence of saturated PS species in the tegument (primarily at the ventral side) and the accumulation of unsaturated ones in the gonads (primarily outer layer of the ovary). A previous study on *S. mansoni* also found PS elevated on the worm surface (Kadesch et al., 2020). PS lipids are generally known for their signaling function, especially in the regulation of apoptosis (Wanderley et al., 2020). Various protozoan parasites use PS lipids to outwit the host immune system and foster infection of host cells (apoptotic mimicry) (Wanderley et al., 2020). Further, it was found that an exposure of PS by protozoan parasites is related to the release of transforming growth factor β (TGF-β) by macrophages, triggering an anti-inflammatory response, as it is done by apoptotic cells (DaMatta et al., 2007). Our spatial lipidomic observations alone do not allow for functional interpretations, however, they can serve as a starting point for further research. It should be confirmed by other methods that the PS lipids in the *F. hepatica* tegument are genuinely exposed to the surface, and if so, investigated whether they play a role in host immune system evasion. If this is the case, further studies could elucidate whether this process is rather a defence mechanism as a reaction to host immune cell attack or an active apoptotic mimicry. For *F. hepatica*, it is reported that apoptosis of host immune cells by excretory secretory products (ESPs) is induced through an increase in reactive oxygen species (ROS), mostly H_2_O_2_ (Bosurgi and Rothlin, 2021). It is not mentioned which components of the ESP trigger this effect, however, we can imagine PS species to be involved in this process due to their apoptosis-regulating properties. Since the ventral tegument side is where the worm suckers are located, it is in direct contact with the host tissue. Possibly, the ventral side therefore needs more protection than the dorsal one. The presence of several unsaturated PS species (38:4, 40:4, and 41:3) in a layer surrounding the liver fluke ovary is of particular interest because this organ has a highly organized architecture. The ovary is surrounded by a basement membrane that clearly separates the gonad from the surrounding tissue. A thin muscle layer surrounds the ovary, adjacent to the basement membrane. Within the ovary, mitotically active germline stem cells (oogonia) are located in a peripheral ring. These oogonia give rise to early primary oocytes that further develop to late primary oocytes, which fill the center of the ovary (Hanna, 2015). In human cells, PS 38:4 is one of the lipids involved in cell division regulation (Atilla-Gokcumen et al., 2014; Kimura et al., 2016). The abovementioned PS species may therefore represent lipids characteristic for the ovary-associated muscle layer. The spatial pattern of detected signals further suggests that the stem-cell layer is characterized by PC 38:3, while late primary oocytes in the center of the ovary are characterized by PC 42:4. Of note, it was shown that the lipidome of human cells changes with the cell cycle (Kimura et al., 2016), and arachidonoyl-containing PCs play a role in controlling the cell cycle (Koeberle et al., 2013). Even though we cannot derive functional information from our spatial data, they might point towards a similar lipid-mediated cell-cycle regulation in parasitic flatworms like *F. hepatica*.

### Different saturation of phosphatidylinositols in the tegument and the gonads

For PI lipids we also noticed the saturated species (e.g., PI 32:0, PI 34:0) present in the tegument while unsaturated ones (e.g., PI 34:1, PI 36:1) were localized predominantly in the gonads). The reason for these differences in phospholipid saturation is not known. Generally, a higher degree of unsaturation in phospholipids increases lipid and protein lateral diffusion velocity (Filippov et al., 2007). However, these effects seem small and are also very dependent on other lipid properties such as chain lengths (Harayama and Antonny, 2023). A high degree of saturation is also associated with a low tendency for lipid peroxidation which might be beneficial for an environmentally exposed tissue like the tegument.

PI lipids are constituents of glycoinositolphospholipids (GPI) that anchor surface proteins. Three major surface antigens of *S. mansoni* were found linked to the membrane by GPIs (Pearce and Sher, 1989). In *T. cruzi*, mucin proteins coat the entire surface of the parasite where they are involved in immune evasion (Cardoso et al., 2016). Mucin genes have been demonstrated to occur in both, *S. mansoni* and *F. hepatica* (Cancela et al., 2015), suggesting a similar function on the parasitic surface and a need for GPIs and PIs in the tegument.

### Lipids with unclear function

We also detected several TGs in *F. hepatica*, all showing the same spatial distribution in the parasite’s parenchyma. TG lipids are usually used as energy storage entities. It is not clear, however, whether TGs can actually still be used as energy source by adult *Fasciola* since despite having all necessary enzymes for β oxidation left from earlier life stages with aerobic metabolism, the pathway does not seem active anymore (Barrett and Korting, 1976). (Very-) long chain fatty acids (VLCFAs) and FAs with odd-numbered carbon chains incorporated into higher lipid species were already reported in helminths, including *F. hepatica* in the literature (Kadesch et al., 2020; Kokova et al., 2021; Luh et al., 2024). To our knowledge, nothing is mentioned in the literature about the function of both odd-chain FAs and VLCFAs in parasites. However, in the nematode worm *C. elegans*, endogenous molecules containing VLCFA and Long Chain-Polyunsaturated Fatty Acids (LC-PUFAs) were associated with a longer life span, an barrier function, and efficient neurotransmission (Lesa et al., 2003; Hillyard and German, 2009; Kage-Nakadai et al., 2010; Wang et al., 2021).

Taken together, our lipid tissue atlas identified new lipid profiles and patterns for several organs and even potential cell types in the adult life stage of *F. hepatica*. The data also stimulate future research questions, ranging from the role of certain lipid types in the parasite’s physiology and its cell cycle control to functions in host-parasite interaction.

### Limitations of the study

The analyses in this study were non-quantitative and do not identify lipid species to defined structural isomers with acyl chain arrangements and double bond positions.

Furthermore, since we analysed the bulk sample with LC-MS/MS, isobaric lipids from other locations in the tissue may have been co-attributed to signals from the organ ROIs.

The organ ROIs were not defined with anatomical precision and may therefore slightly overlap with neighbouring tissues. Tissue features smaller than our lateral resolution of 7 or 8 µm were not distinguishable.

## Data Availability

The MS imaging data are publicly available via this link: https://metaspace2020.org/project/morawietz-2026. The LC-MS/MS data files can be viewed in the MassIVE repository (https://massive.ucsd.edu/ProteoSAFe/static/massive.jsp?redirect=auth) using the following dataset identifier: MSV000103384.
