# Supplementary material for: Spatial lipidomics of Fasciola hepatica using high-resolution AP-SMALDI Orbitrap MSI – a lipid tissue atlas
Source: Front Cell Infect Microbiol. 2026 Oct 22;16:1934046. doi: 10.3389/fcimb.2026.1934046 (PMC13735547; doi:10.3389/fcimb.2026.1934046)
Supplement: Supplementary file 1 [file DataSheet1.zip › Supplementary images 1.pdf]

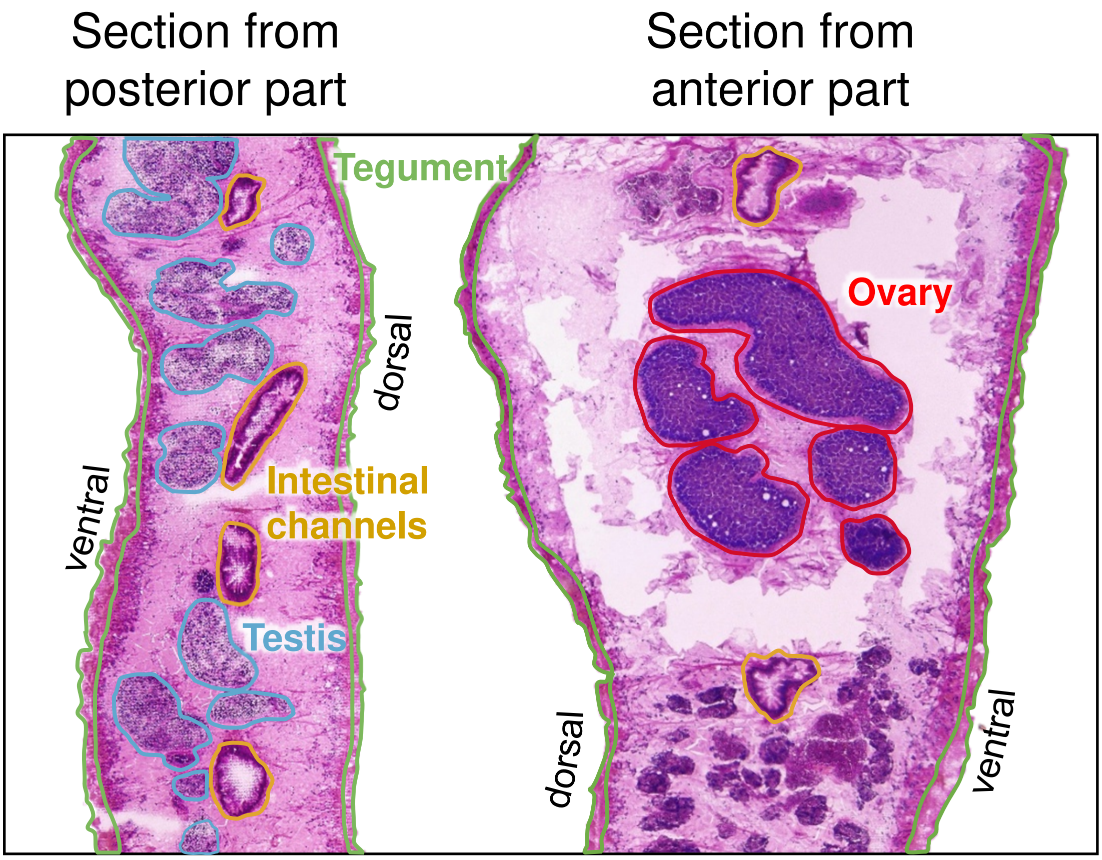

Section from posterior part
Section from anterior part
Tegument
dorsal
ventral
Intestinal channels
Testis
Ovary
dorsal
ventral

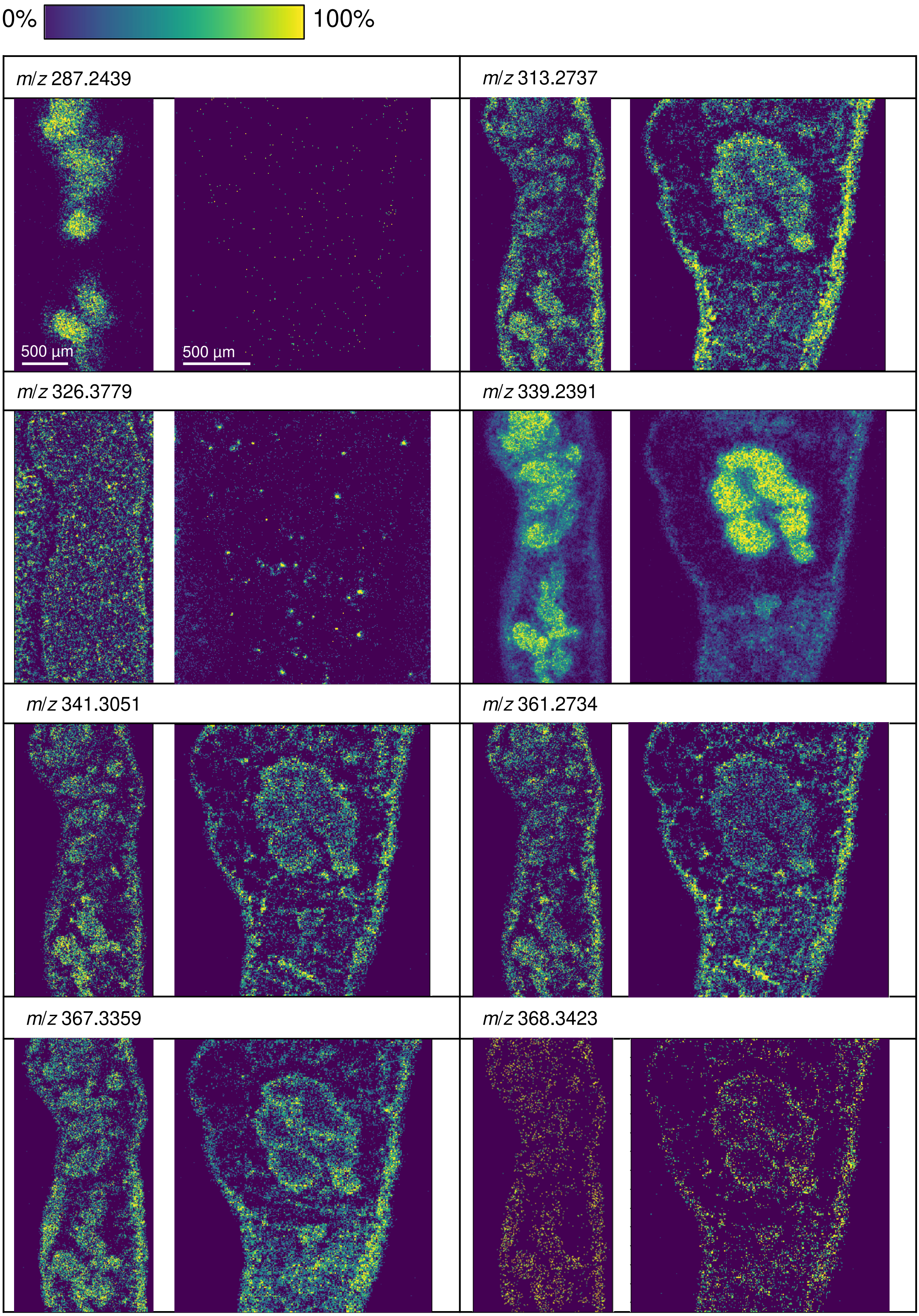
0%
100%
m/z 287.2439
500 µm
500 µm
m/z 313.2737
m/z 326.3779
m/z 339.2391
m/z 341.3051
m/z 361.2734
m/z 367.3359
m/z 368.3423

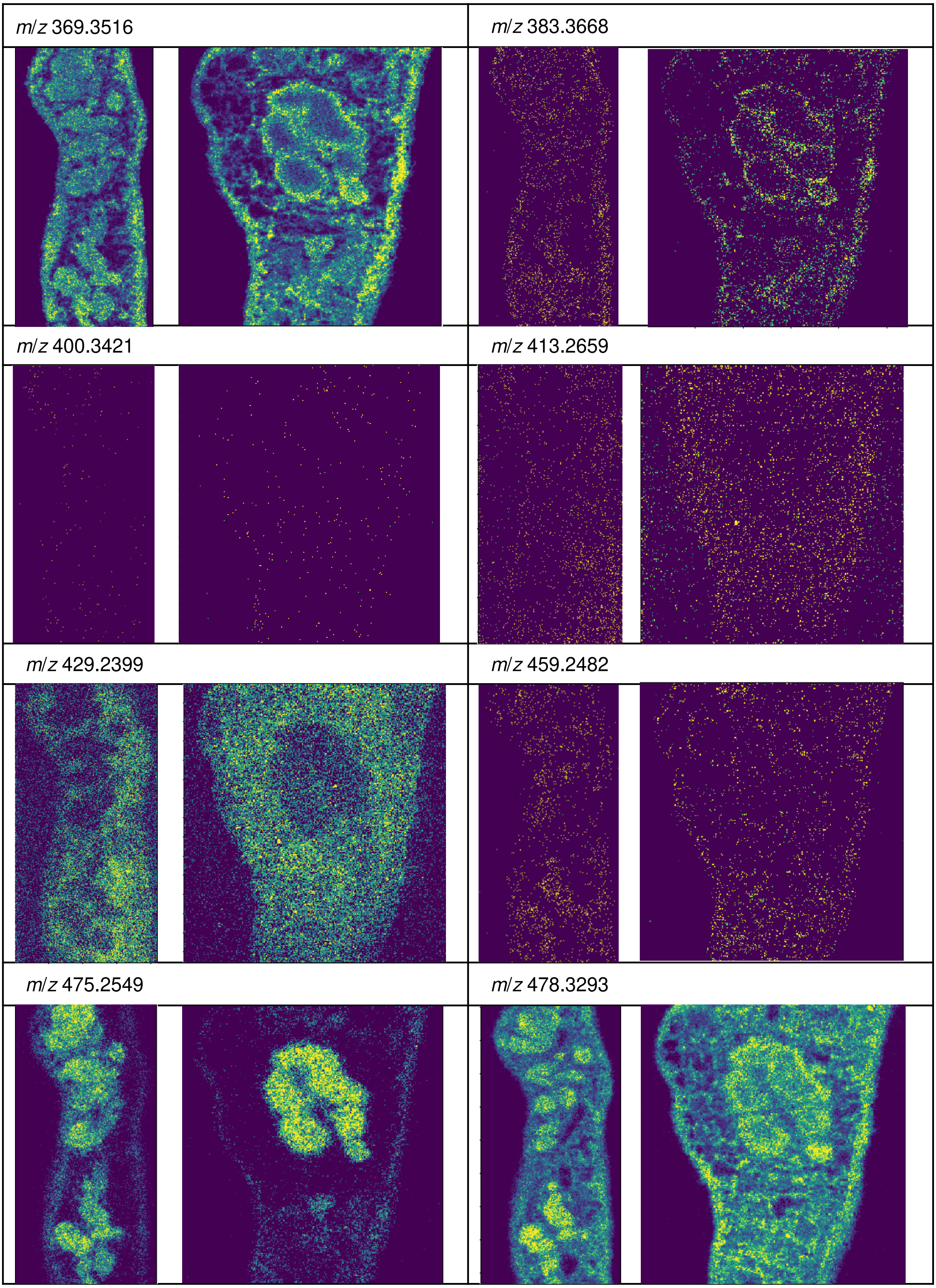

m/z 369.3516
m/z 383.3668
m/z 400.3421
m/z 413.2659
m/z 429.2399
m/z 459.2482
m/z 475.2549
m/z 478.3293

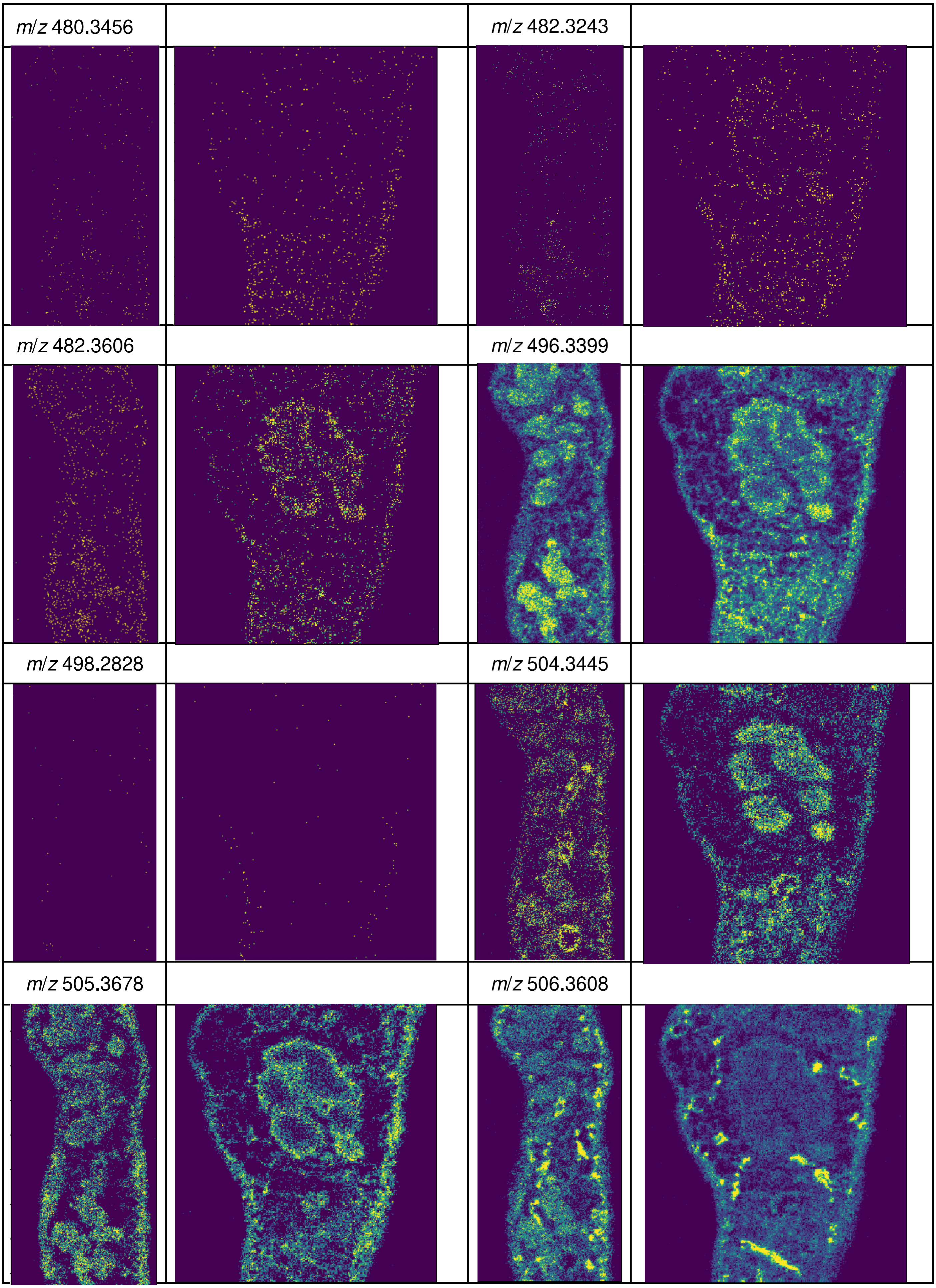
m/z 480.3456
m/z 482.3243
m/z 482.3606
m/z 496.3399
m/z 498.2828
m/z 504.3445
m/z 505.3678
m/z 506.3608

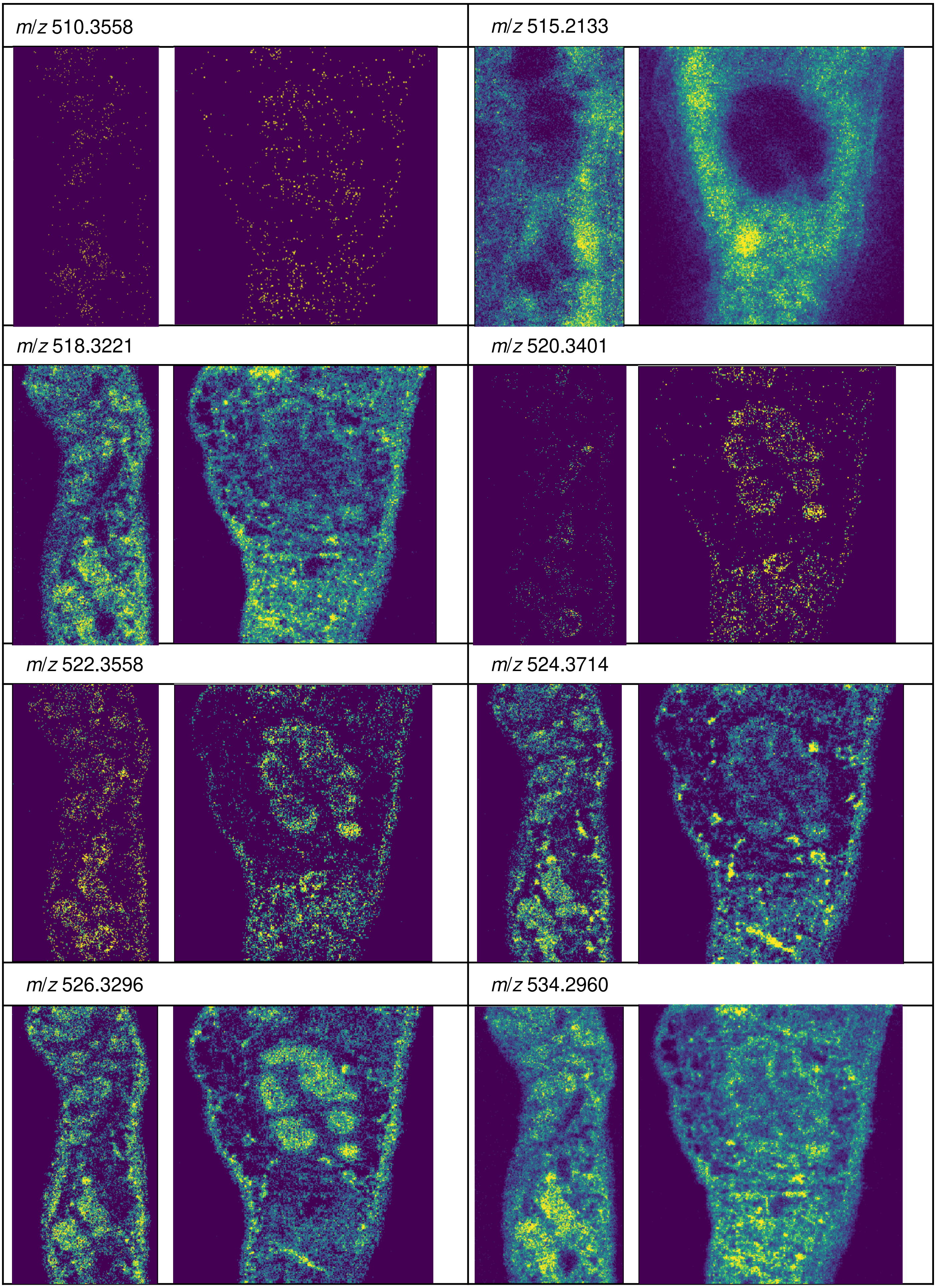
m/z 510.3558
m/z 515.2133
m/z 518.3221
m/z 520.3401
m/z 522.3558
m/z 524.3714
m/z 526.3296
m/z 534.2960

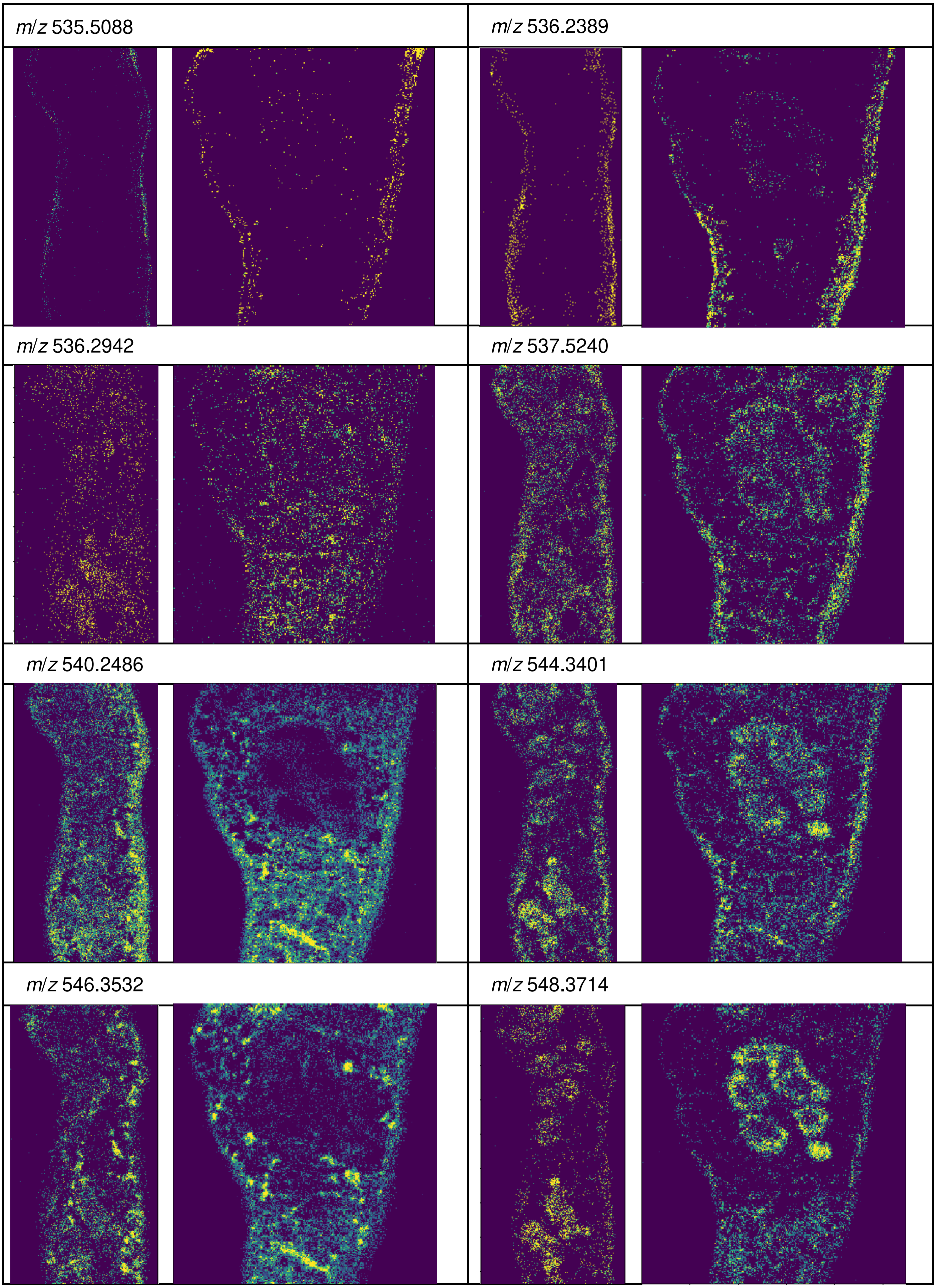
m/z 535.5088
m/z 536.2389
m/z 536.2942
m/z 537.5240
m/z 540.2486
m/z 544.3401
m/z 546.3532
m/z 548.3714

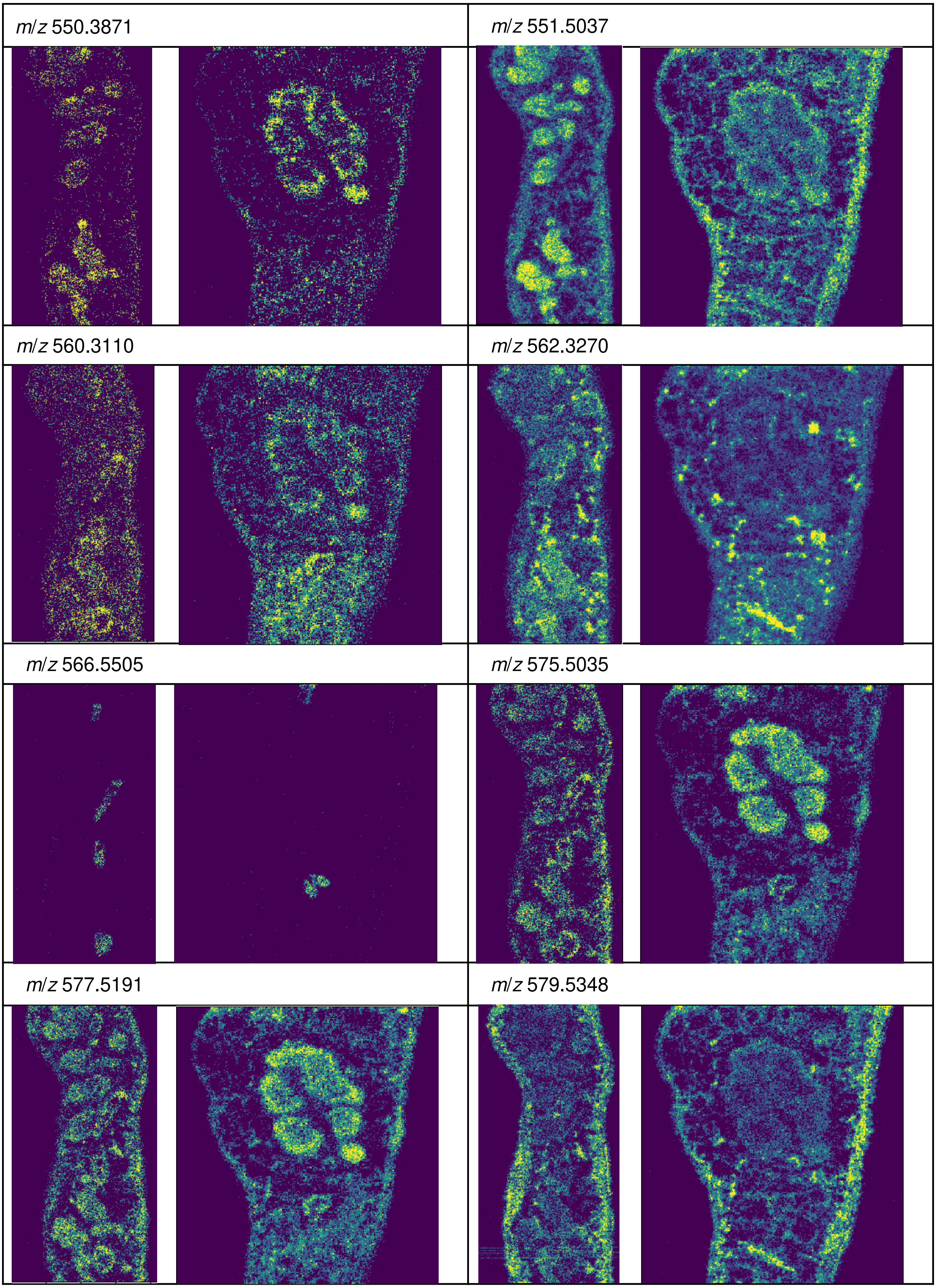

m/z 550.3871
m/z 551.5037
m/z 560.3110
m/z 562.3270
m/z 566.5505
m/z 575.5035
m/z 577.5191
m/z 579.5348

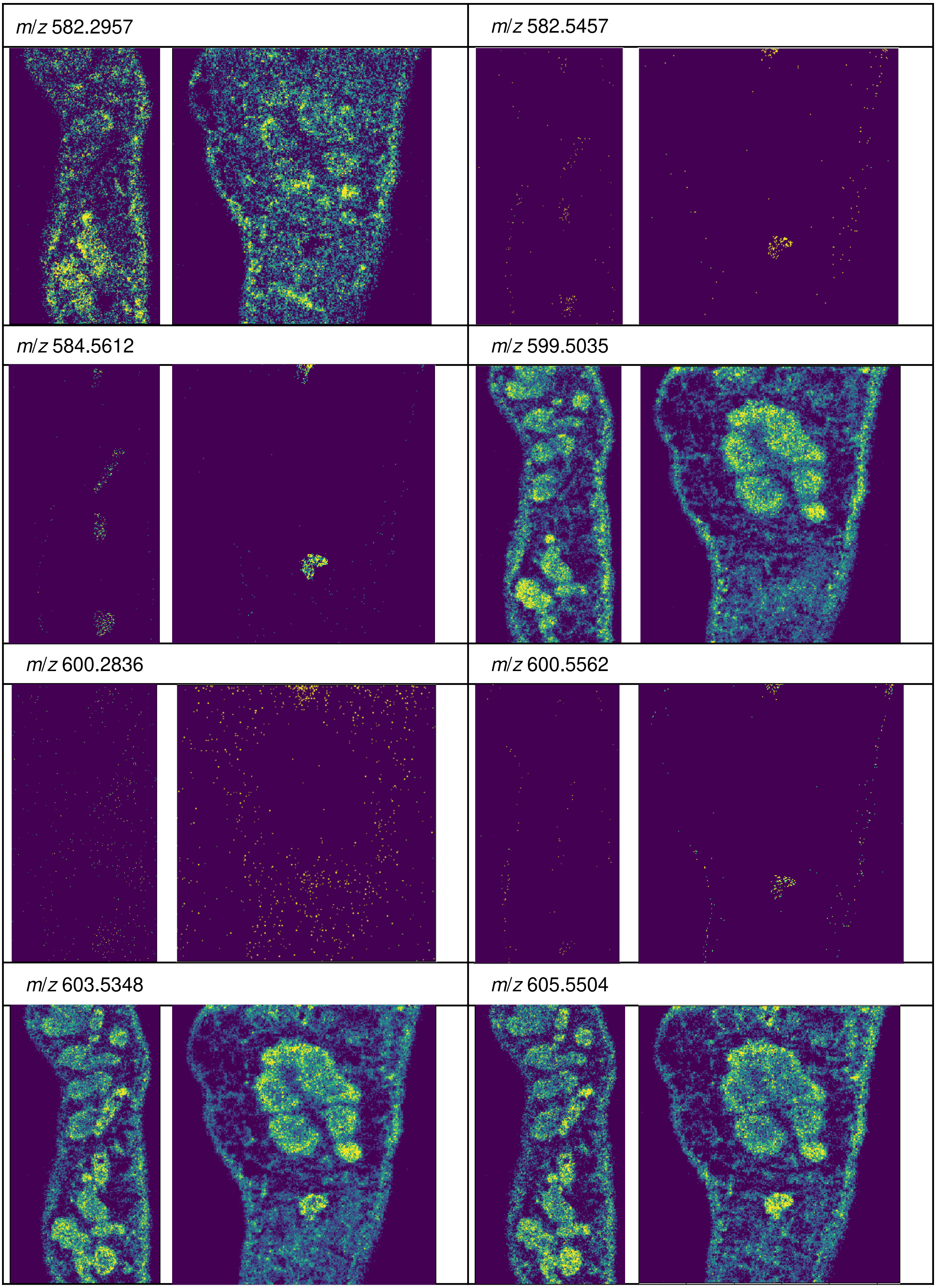
m/z 582.2957
m/z 582.5457
m/z 584.5612
m/z 599.5035
m/z 600.2836
m/z 600.5562
m/z 603.5348
m/z 605.5504

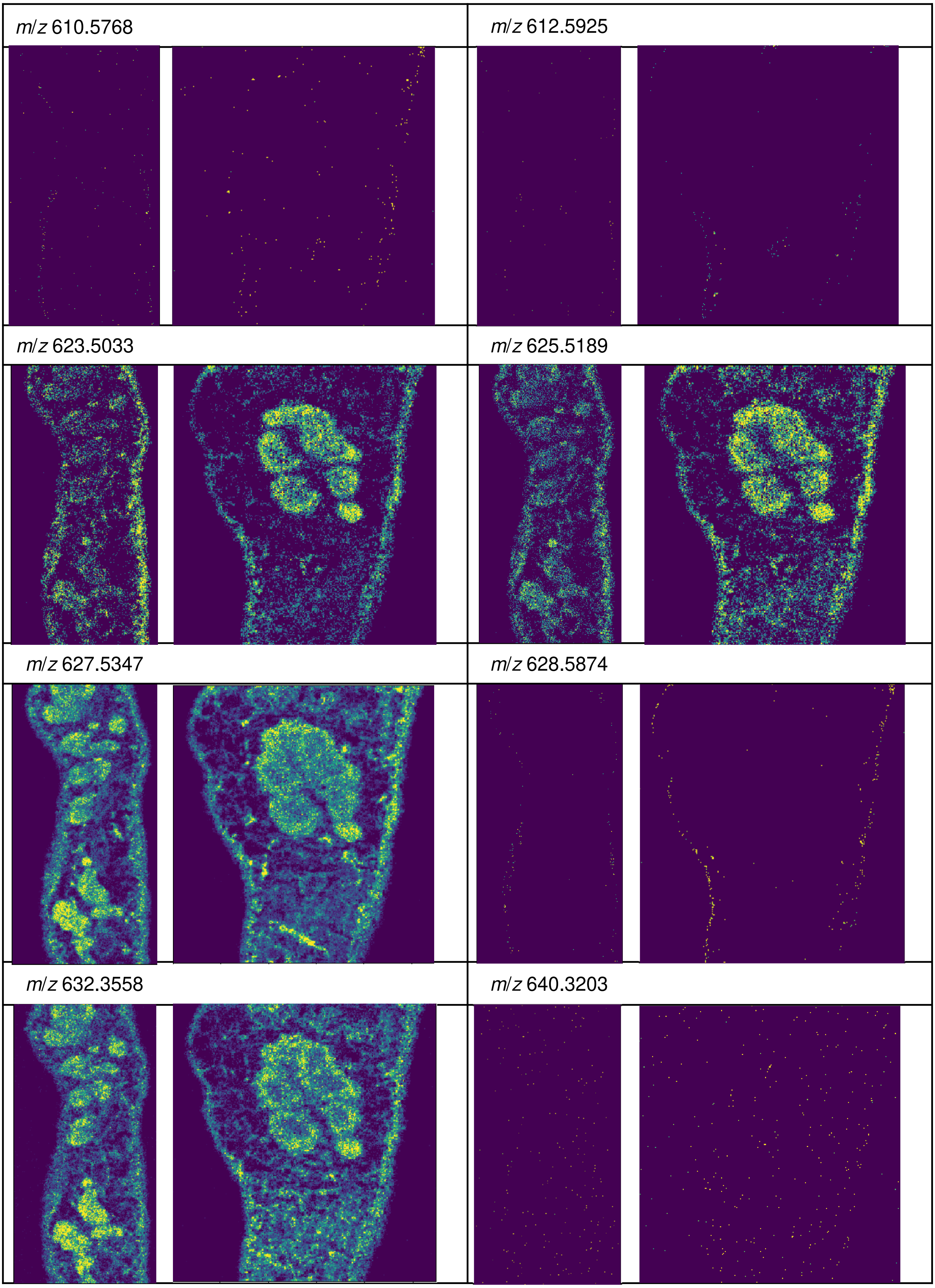

m/z 610.5768
m/z 612.5925
m/z 623.5033
m/z 625.5189
m/z 627.5347
m/z 628.5874
m/z 632.3558
m/z 640.3203

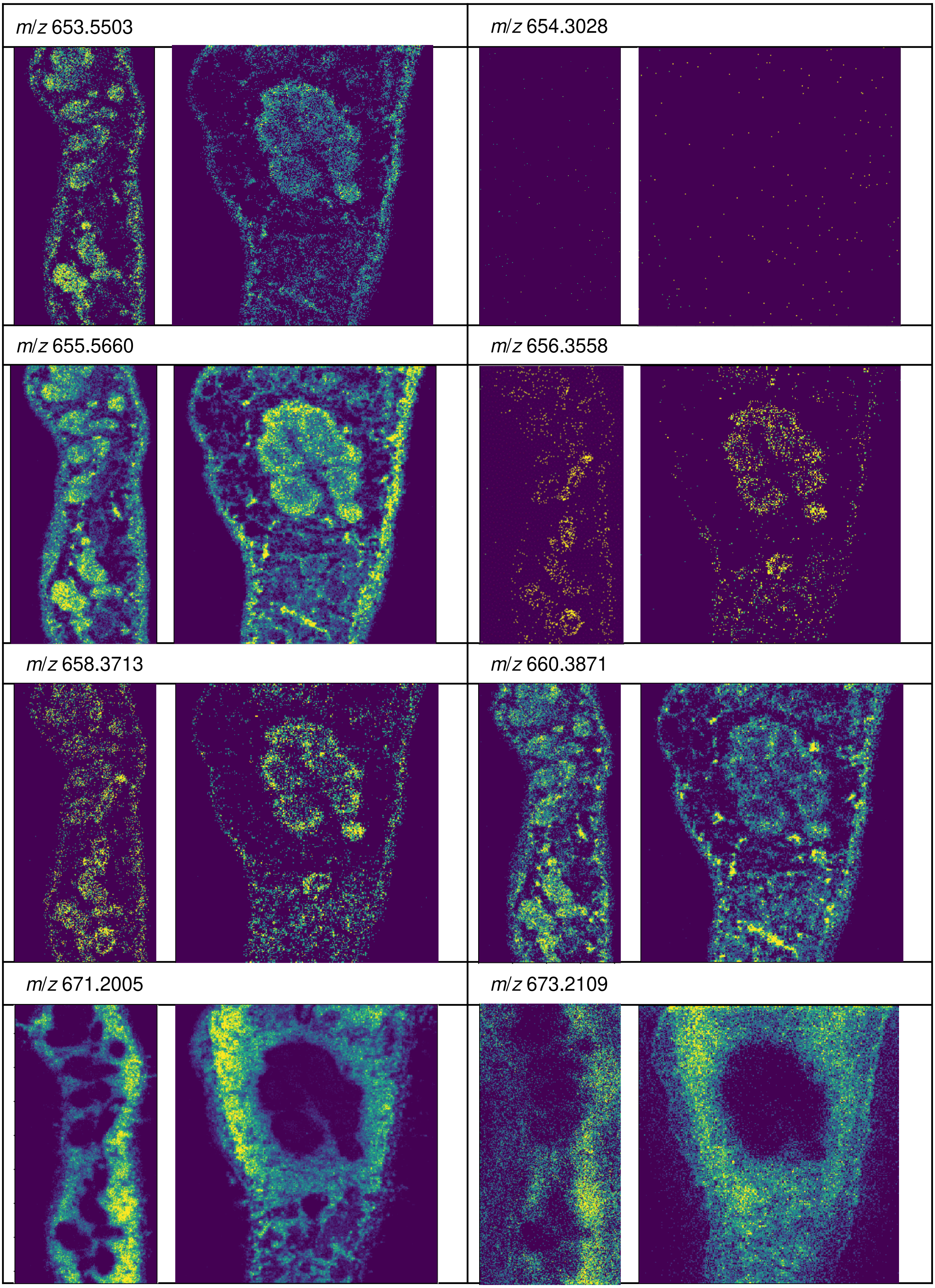
m/z 653.5503
m/z 654.3028
m/z 655.5660
m/z 656.3558
m/z 658.3713
m/z 660.3871
m/z 671.2005
m/z 673.2109

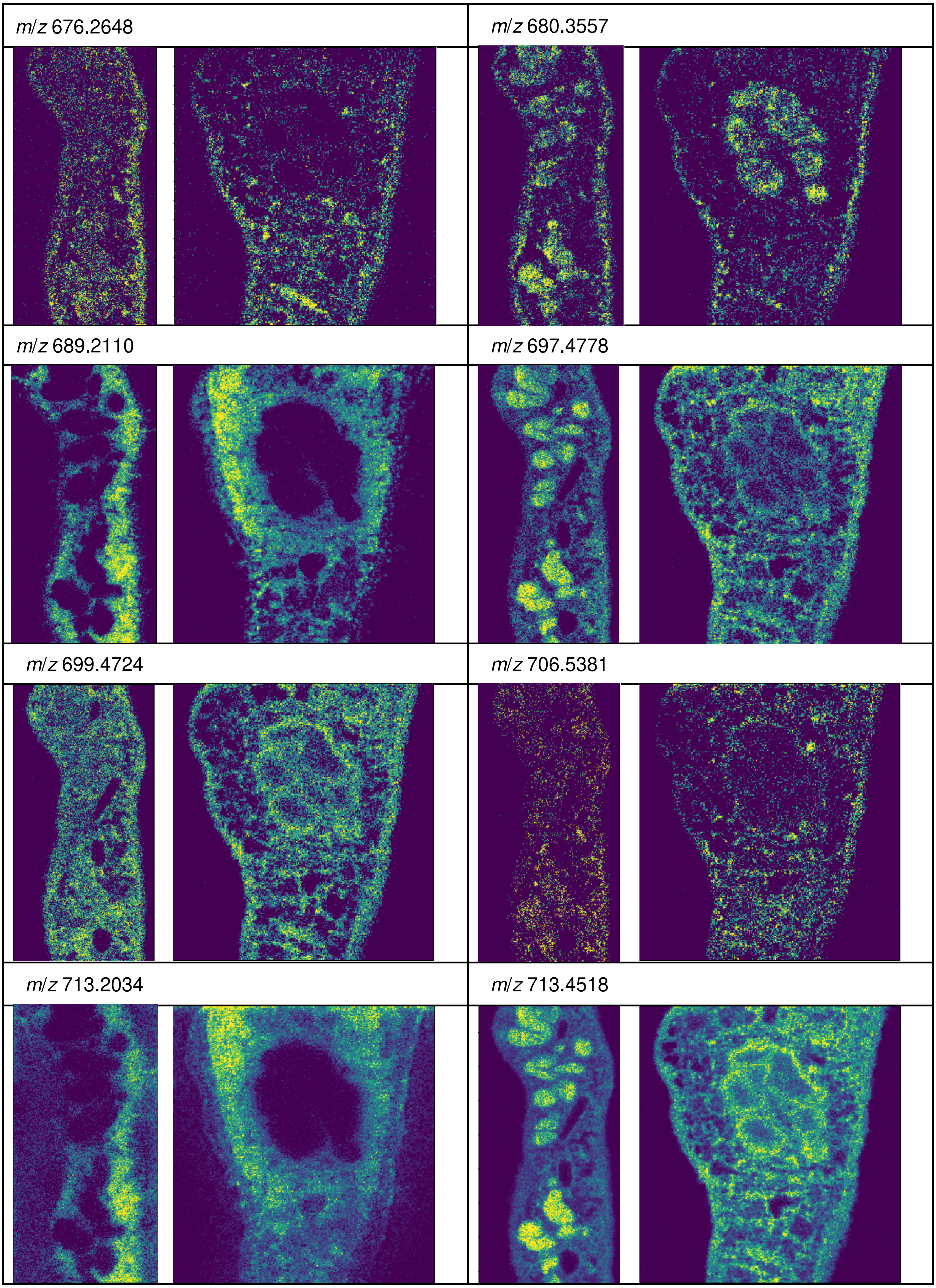
m/z 676.2648
m/z 680.3557
m/z 689.2110
m/z 697.4778
m/z 699.4724
m/z 706.5381
m/z 713.2034
m/z 713.4518

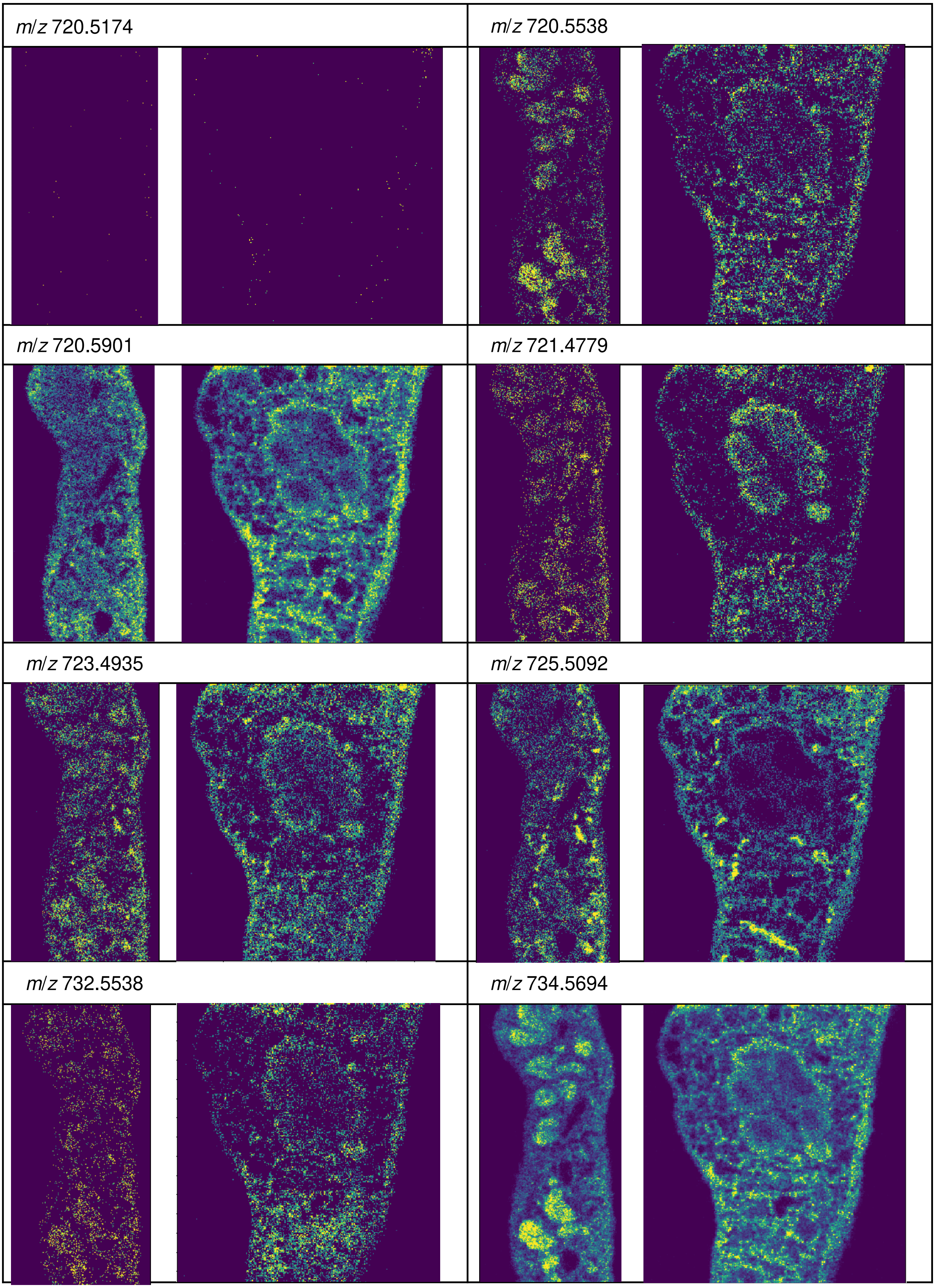
m/z 720.5174
m/z 720.5538
m/z 720.5901
m/z 721.4779
m/z 723.4935
m/z 725.5092
m/z 732.5538
m/z 734.5694

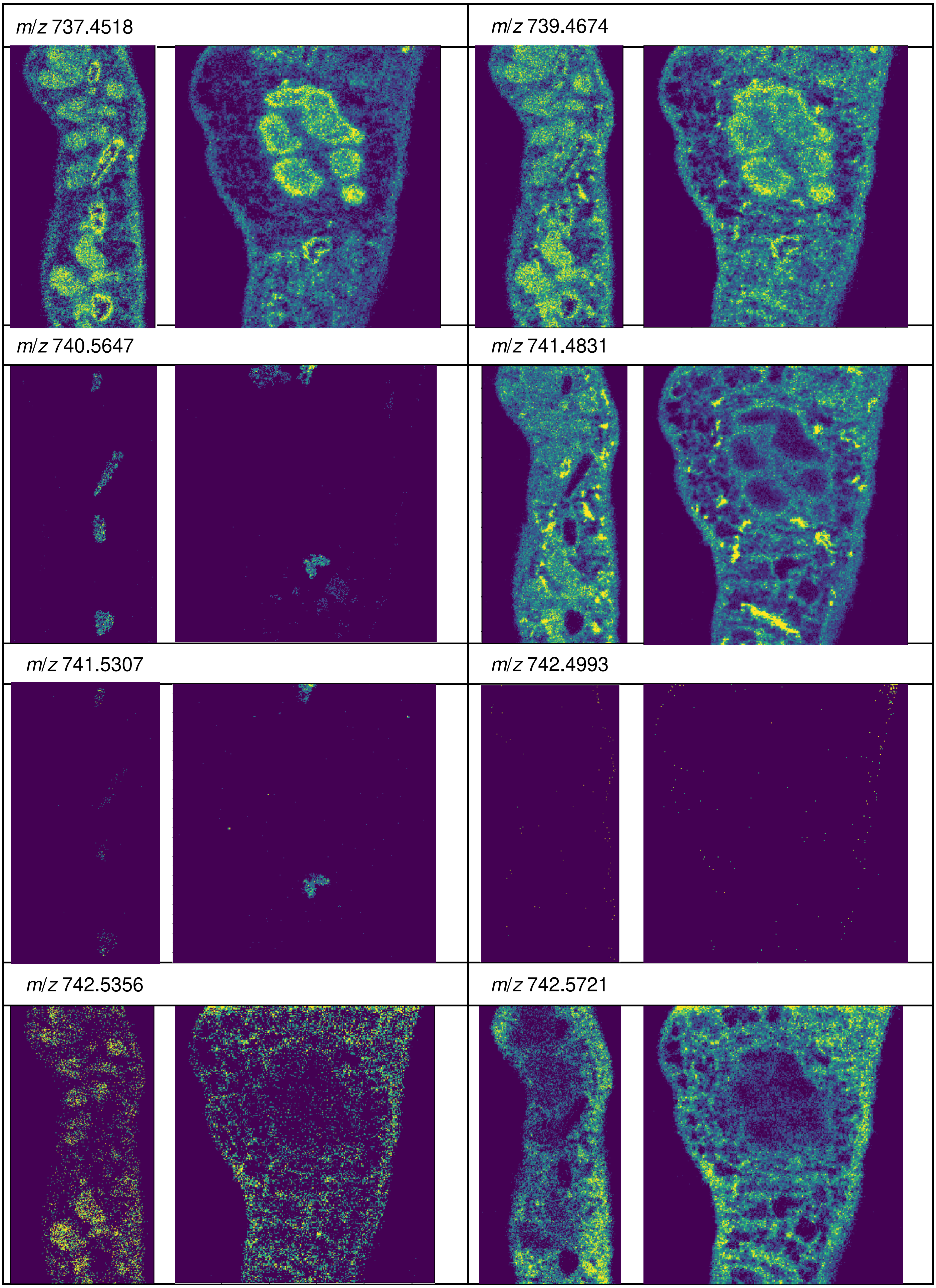
m/z 737.4518
m/z 739.4674
m/z 740.5647
m/z 741.4831
m/z 741.5307
m/z 742.4993
m/z 742.5356
m/z 742.5721

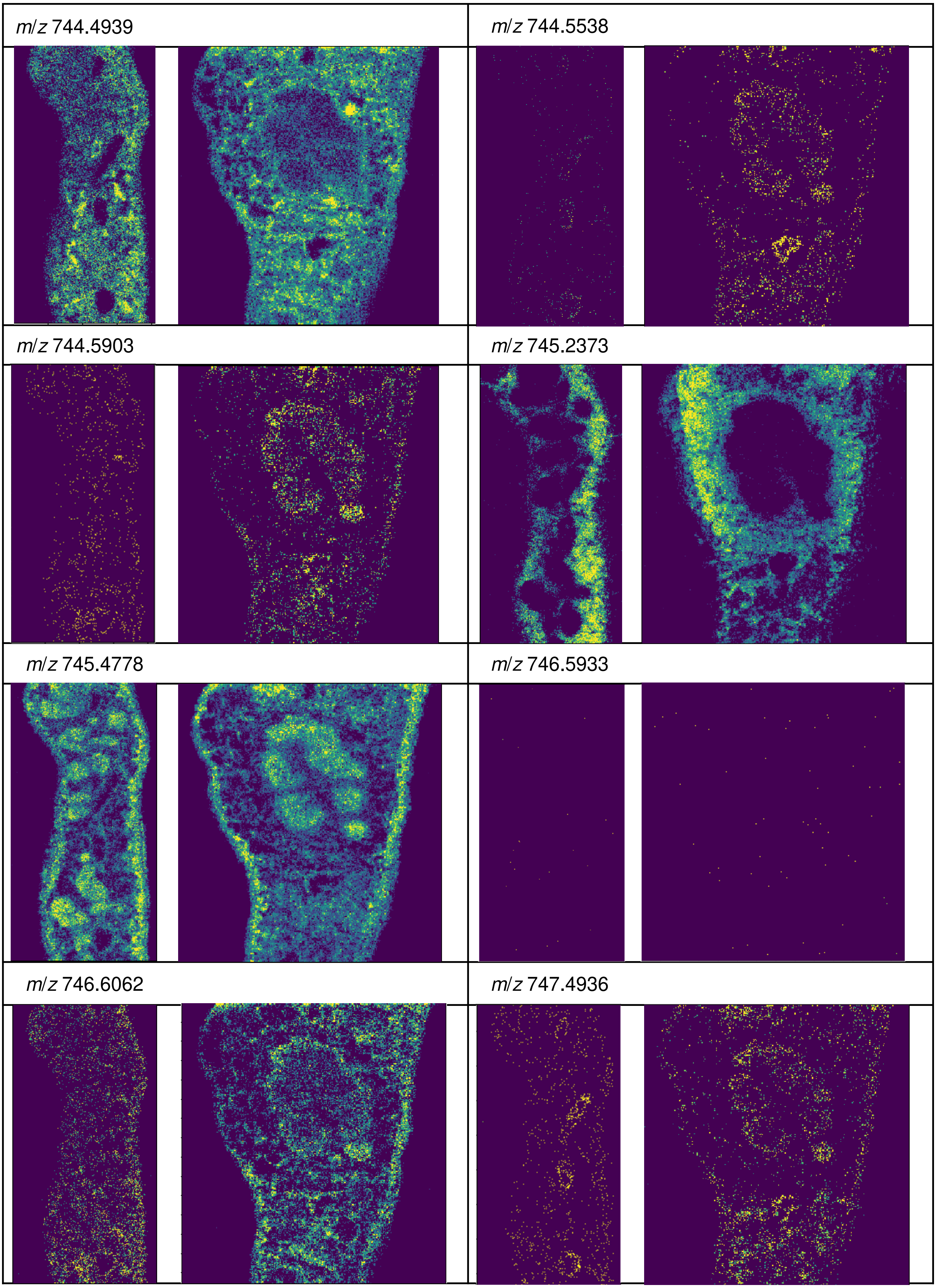

m/z 744.4939
m/z 744.5538
m/z 744.5903
m/z 745.2373
m/z 745.4778
m/z 746.5933
m/z 746.6062
m/z 747.4936

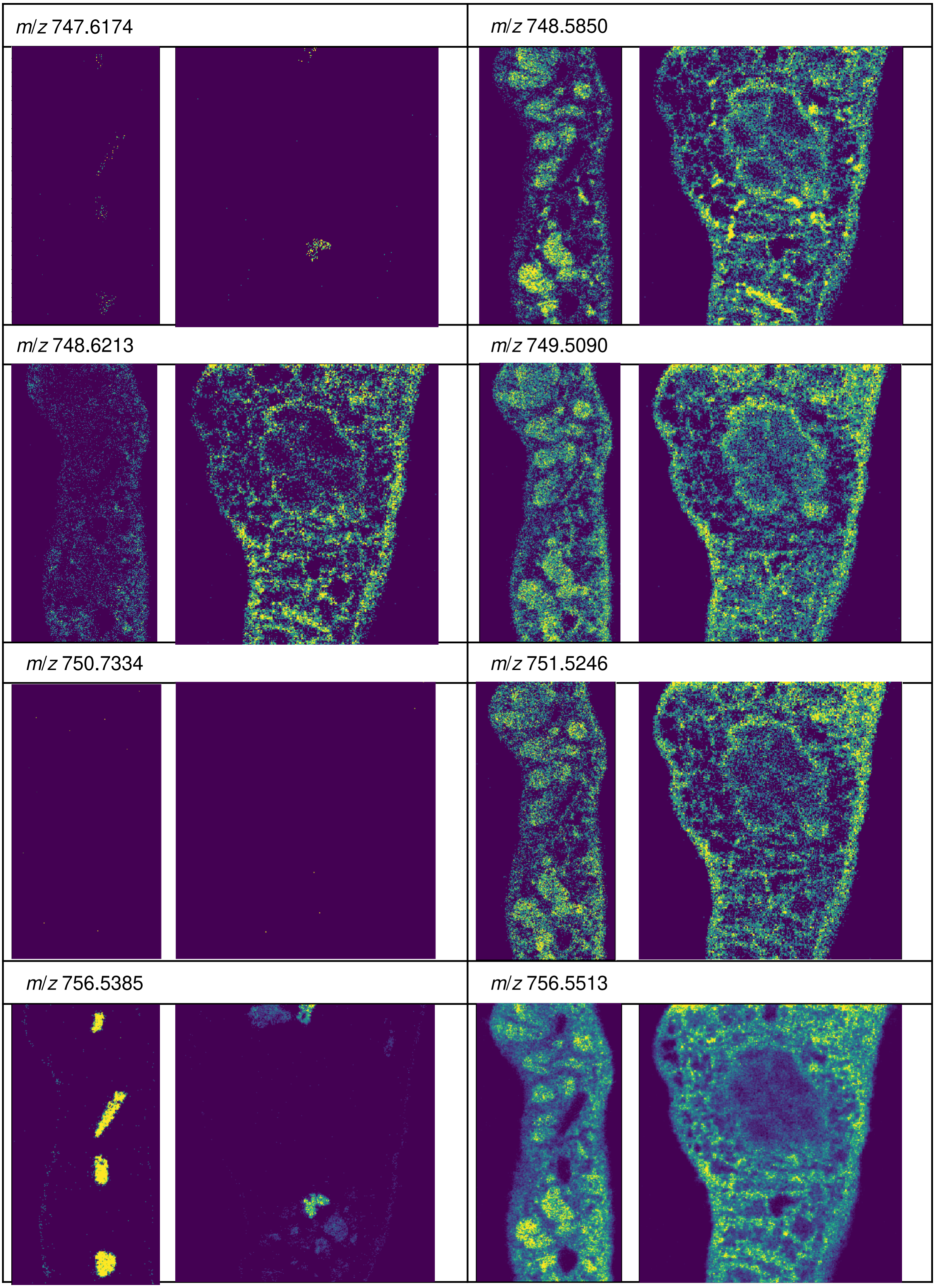
m/z 747.6174
m/z 748.5850
m/z 748.6213
m/z 749.5090
m/z 750.7334
m/z 751.5246
m/z 756.5385
m/z 756.5513

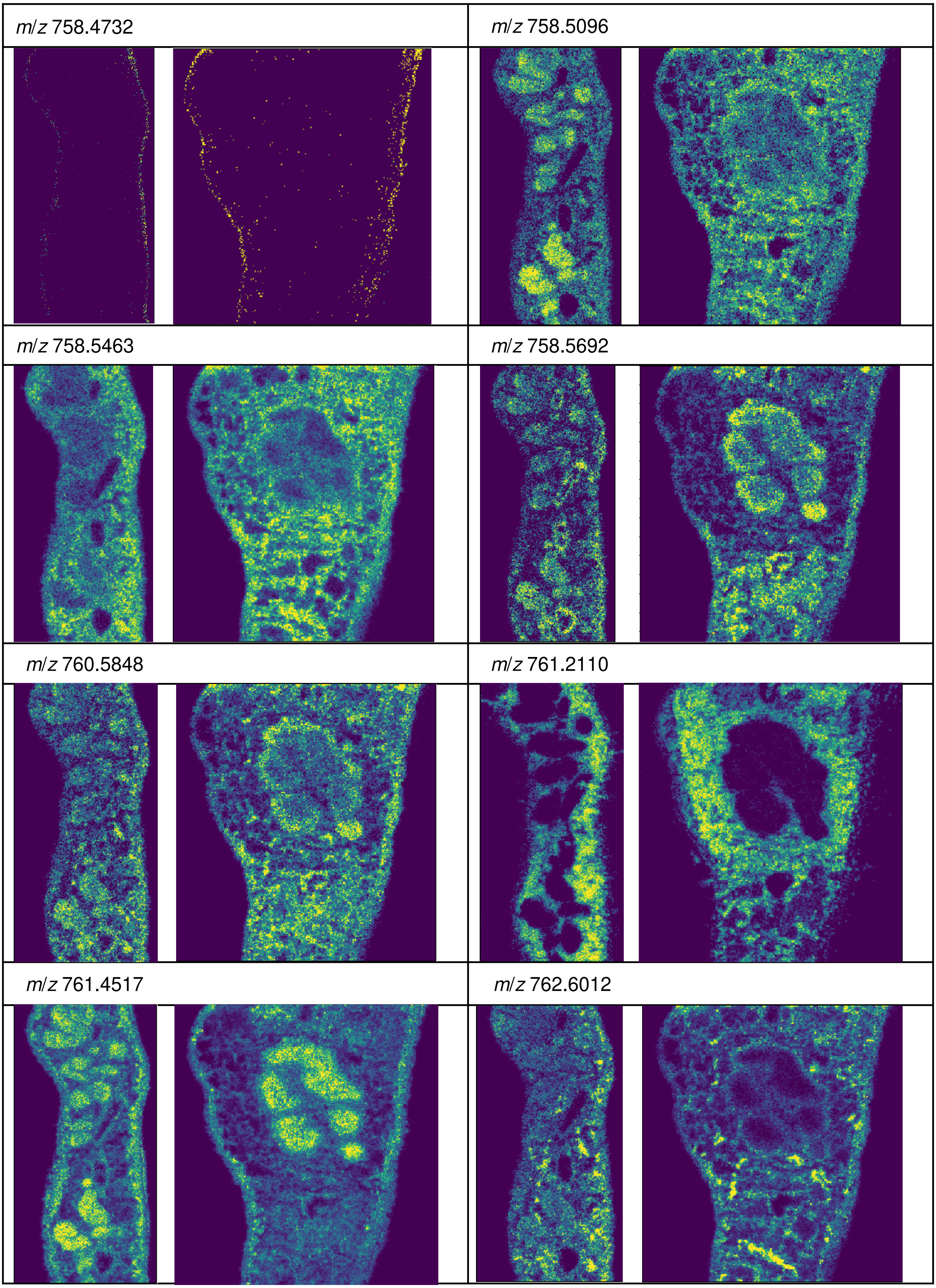

m/z 758.4732
m/z 758.5096
m/z 758.5463
m/z 758.5692
m/z 760.5848
m/z 761.2110
m/z 761.4517
m/z 762.6012

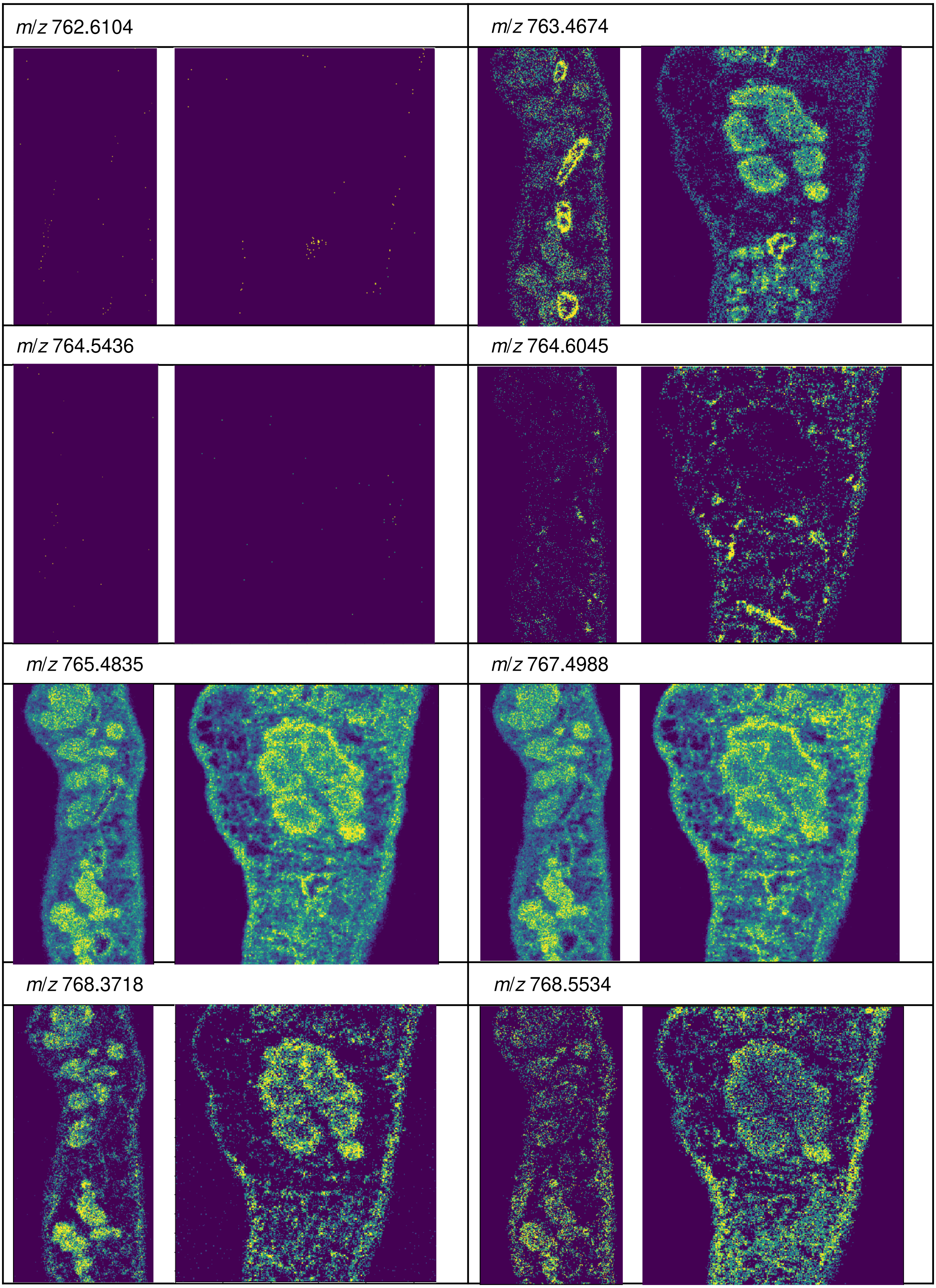

m/z 762.6104
m/z 763.4674
m/z 764.5436
m/z 764.6045
m/z 765.4835
m/z 767.4988
m/z 768.3718
m/z 768.5534

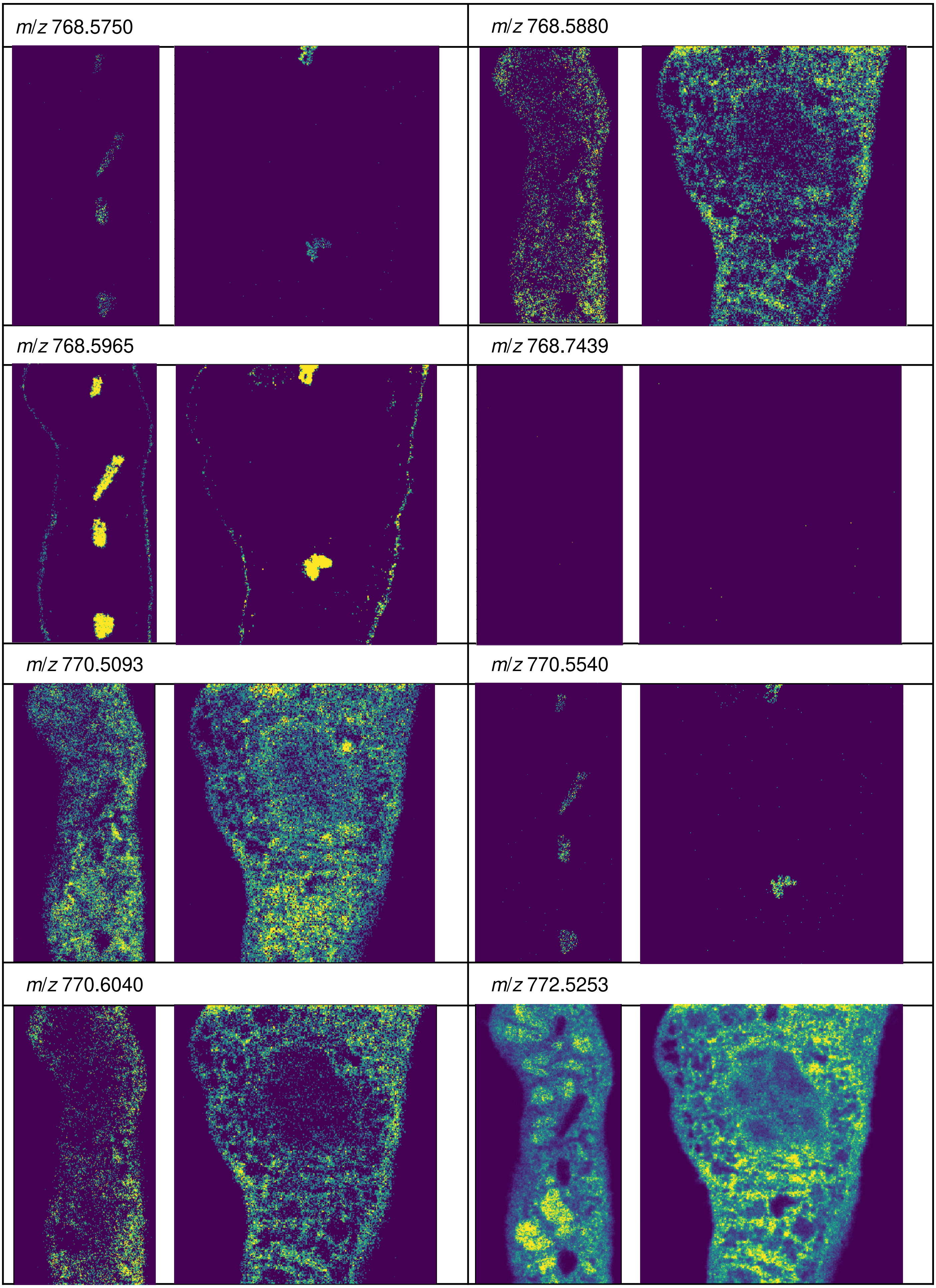
m/z 768.5750
m/z 768.5880
m/z 768.5965
m/z 768.7439
m/z 770.5093
m/z 770.5540
m/z 770.6040
m/z 772.5253

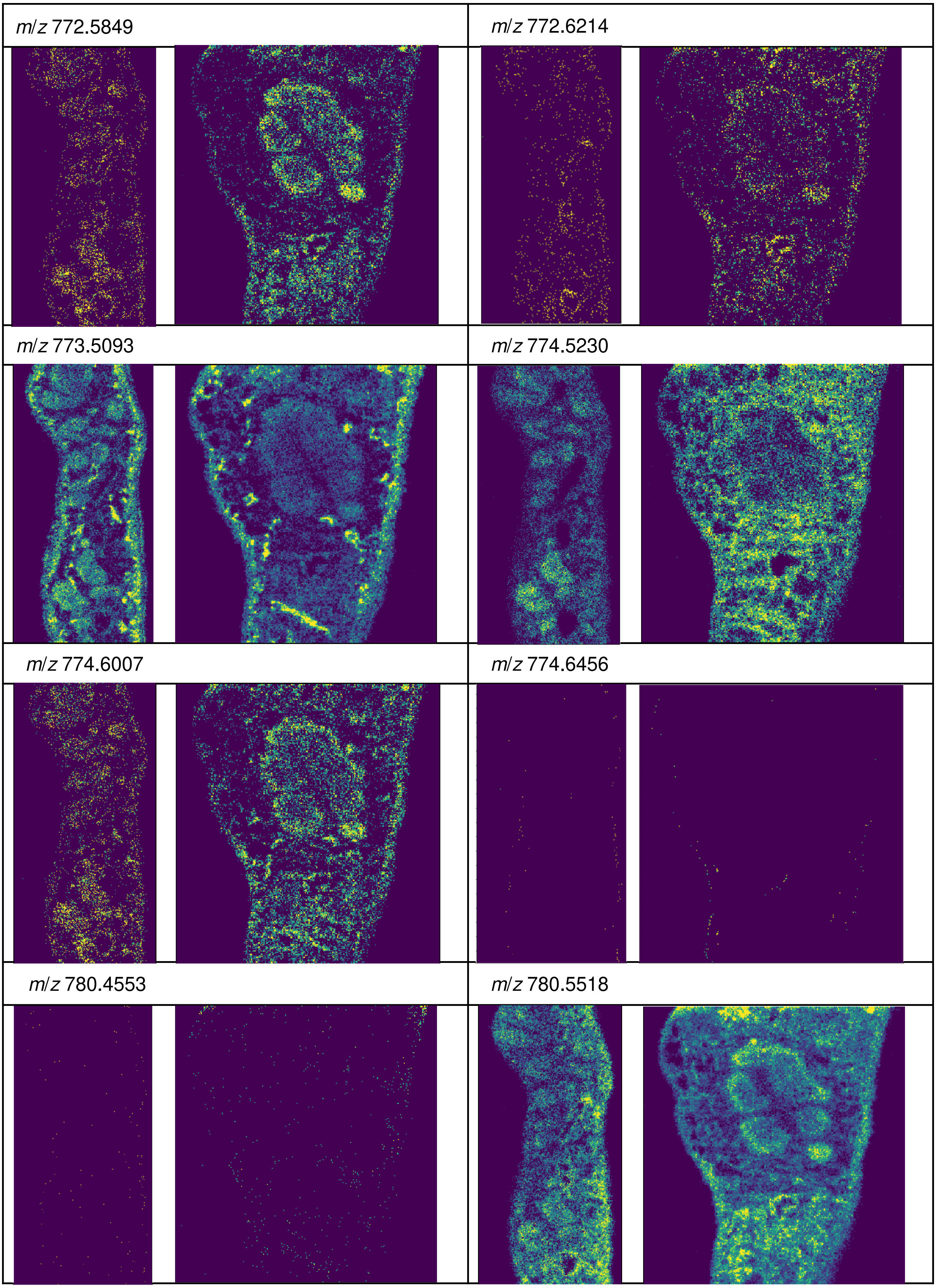

m/*z* 772.5849
m/*z* 772.6214
m/*z* 773.5093
m/*z* 774.5230
m/*z* 774.6007
m/*z* 774.6456
m/*z* 780.4553
m/*z* 780.5518

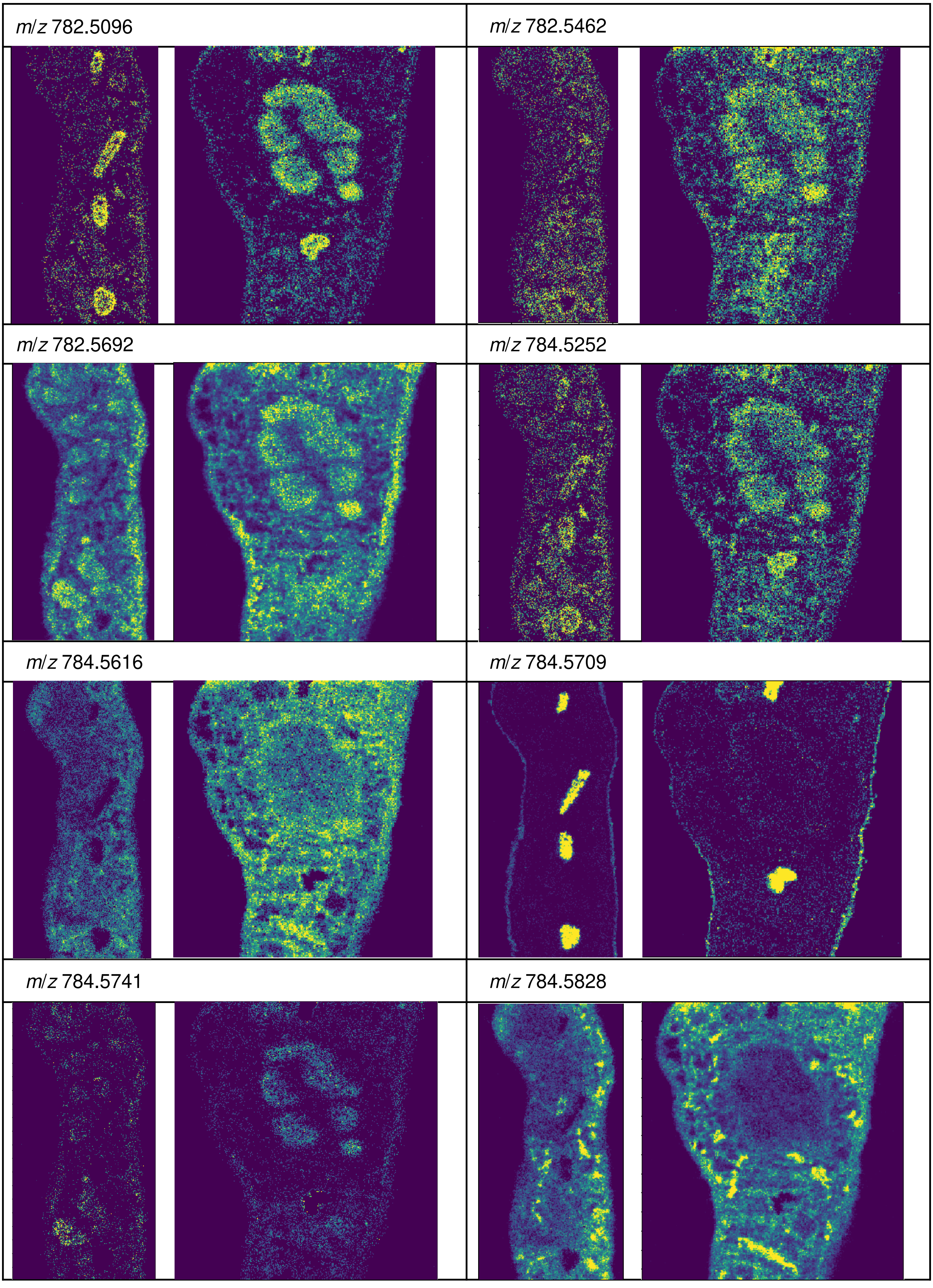

m/z 782.5096
m/z 782.5462
m/z 782.5692
m/z 784.5252
m/z 784.5616
m/z 784.5709
m/z 784.5741
m/z 784.5828

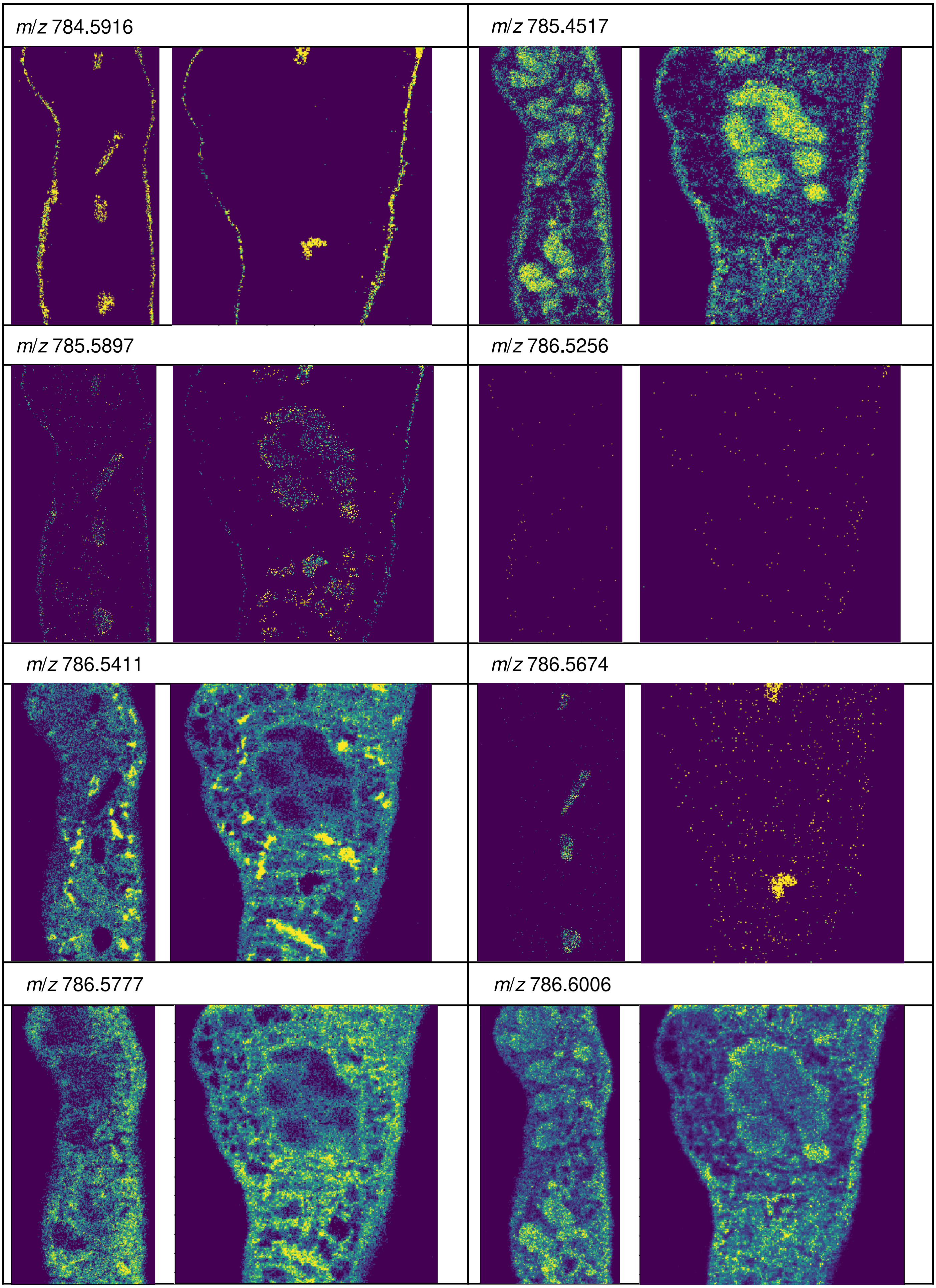
m/z 784.5916
m/z 785.4517
m/z 785.5897
m/z 786.5256
m/z 786.5411
m/z 786.5674
m/z 786.5777
m/z 786.6006

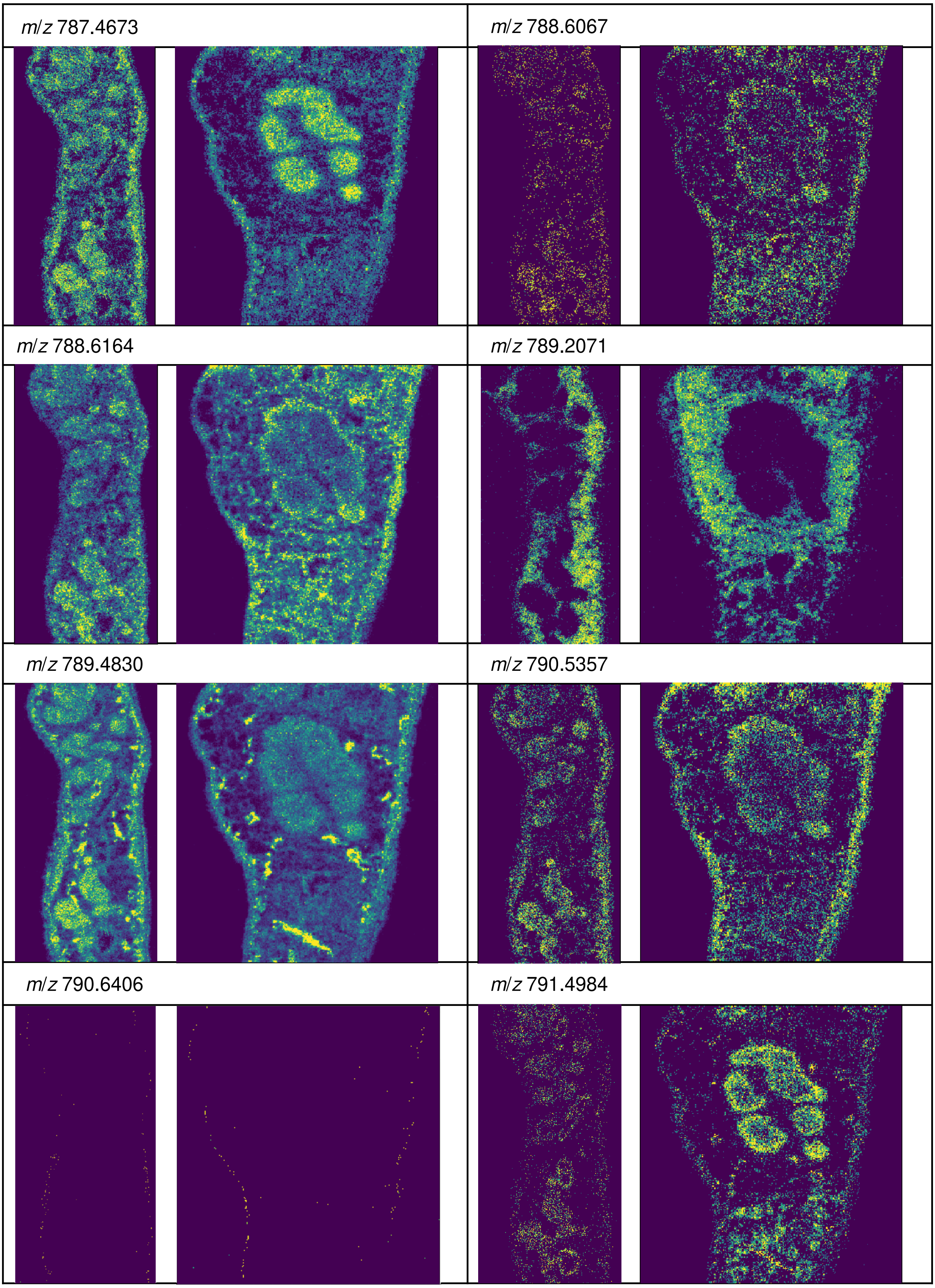
m/z 787.4673
m/z 788.6067
m/z 788.6164
m/z 789.2071
m/z 789.4830
m/z 790.5357
m/z 790.6406
m/z 791.4984

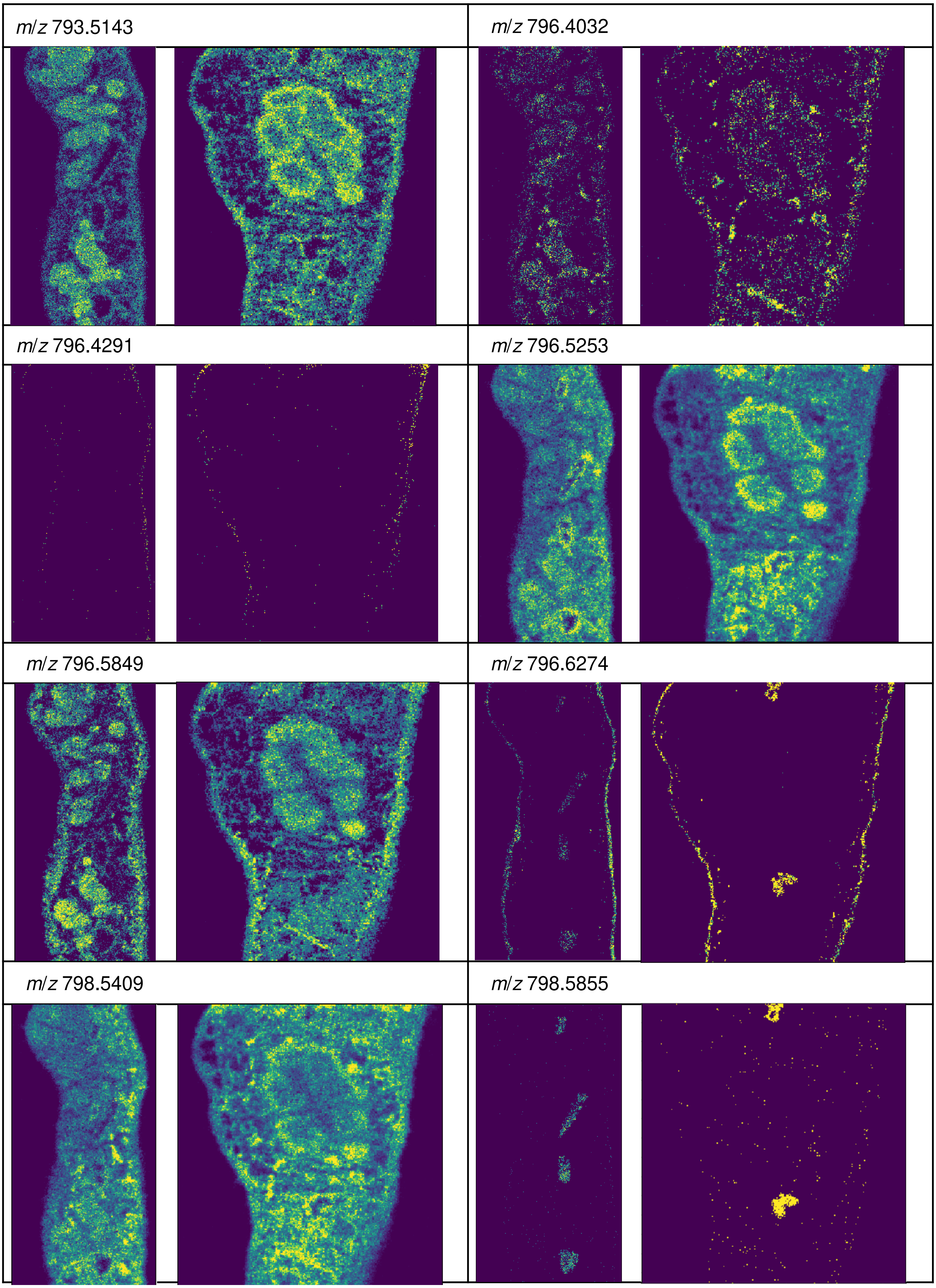

m/z 793.5143
m/z 796.4032
m/z 796.4291
m/z 796.5253
m/z 796.5849
m/z 796.6274
m/z 798.5409
m/z 798.5855

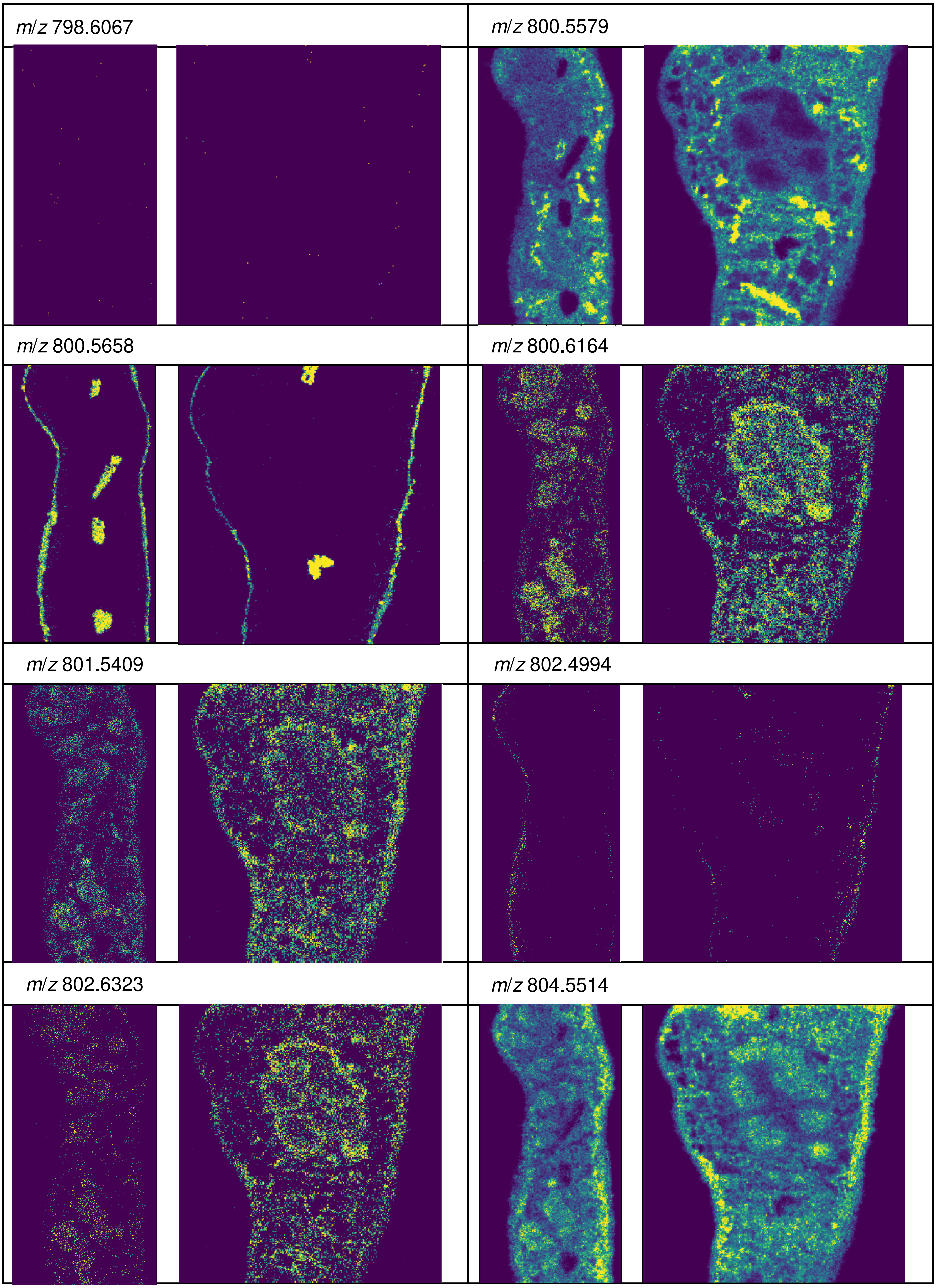
m/z 798.6067
m/z 800.5579
m/z 800.5658
m/z 800.6164
m/z 801.5409
m/z 802.4994
m/z 802.6323
m/z 804.5514

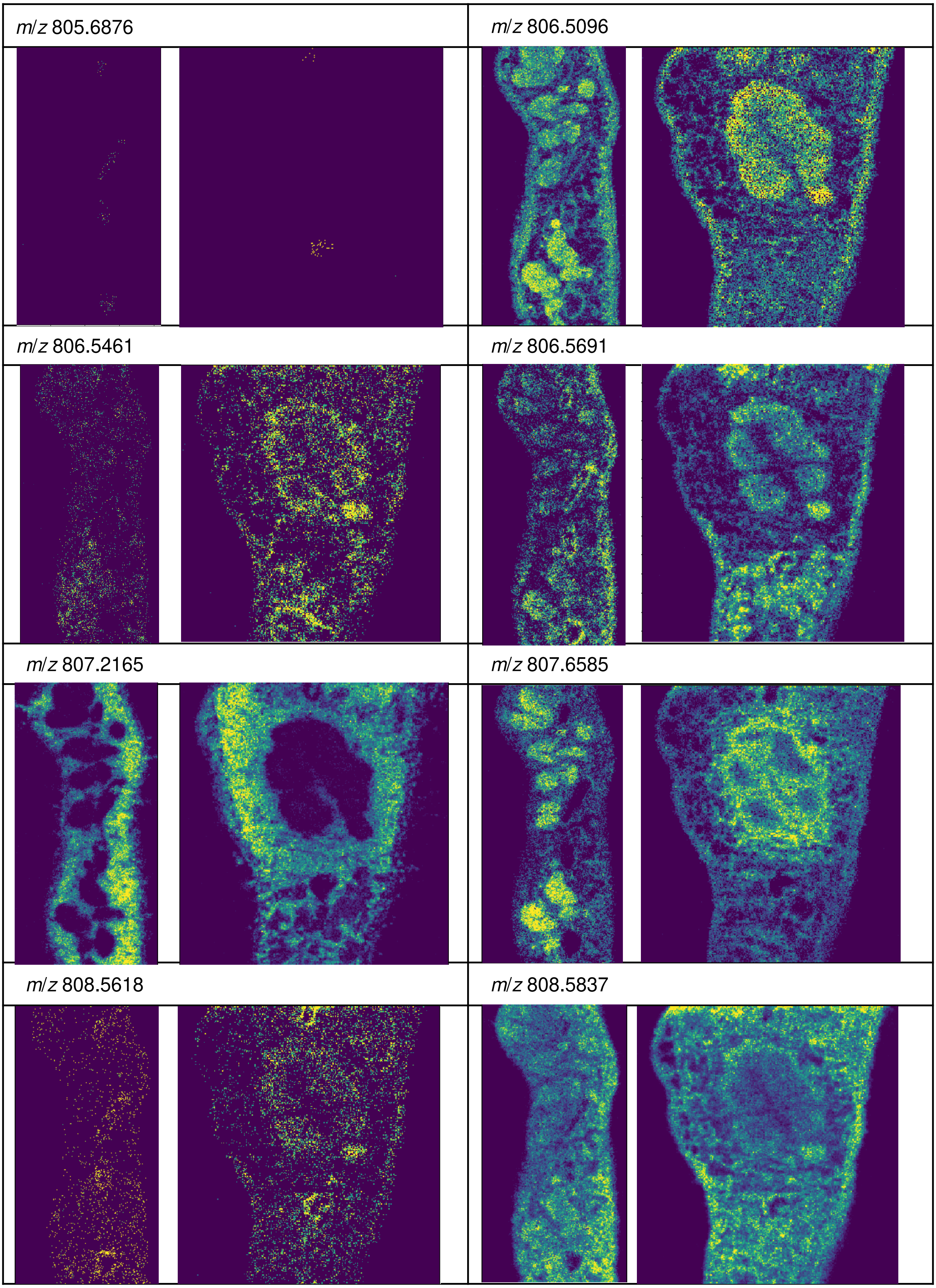
m/z 805.6876
m/z 806.5096
m/z 806.5461
m/z 806.5691
m/z 807.2165
m/z 807.6585
m/z 808.5618
m/z 808.5837

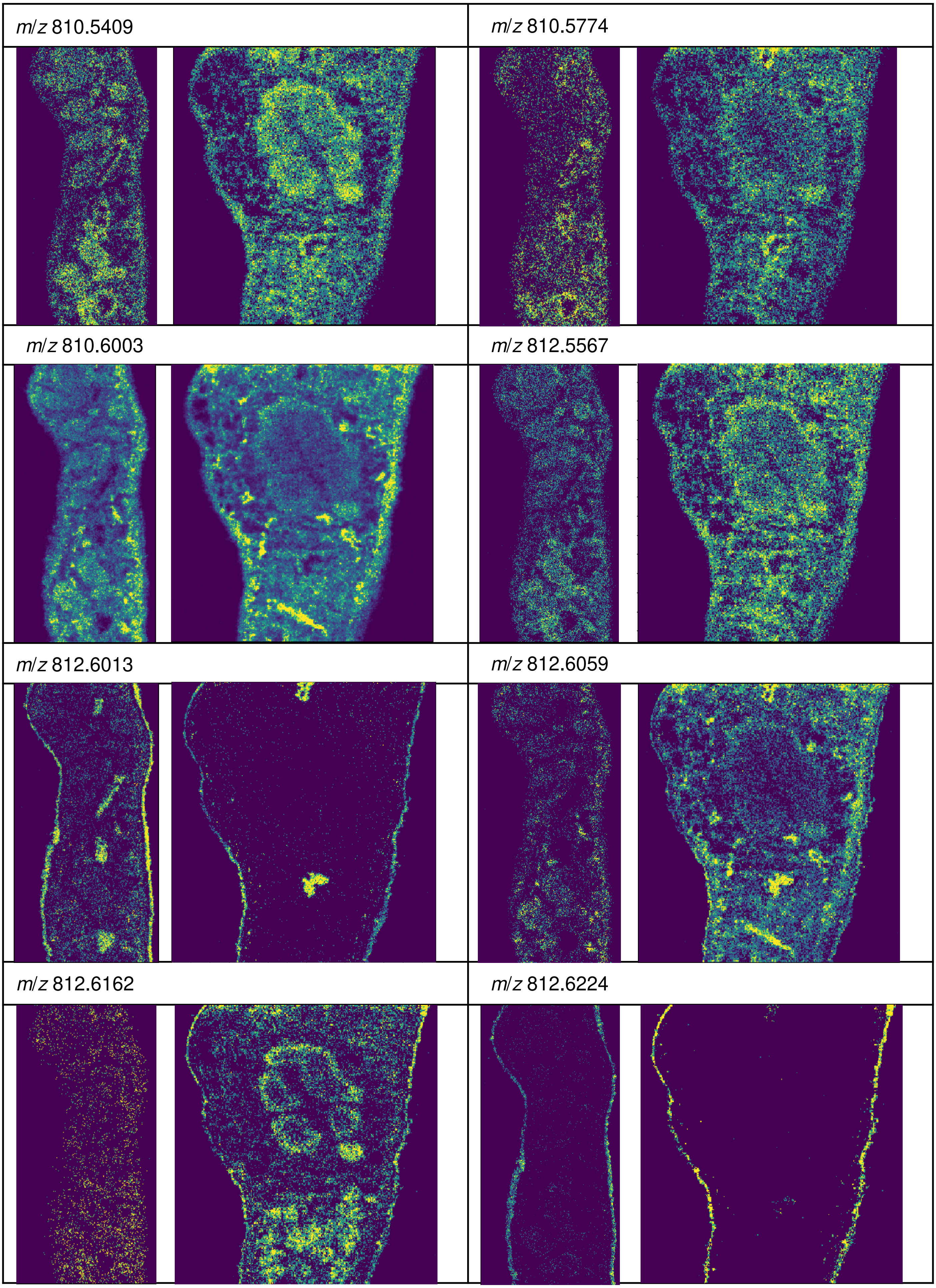
m/z 810.5409
m/z 810.5774
m/z 810.6003
m/z 812.5567
m/z 812.6013
m/z 812.6059
m/z 812.6162
m/z 812.6224

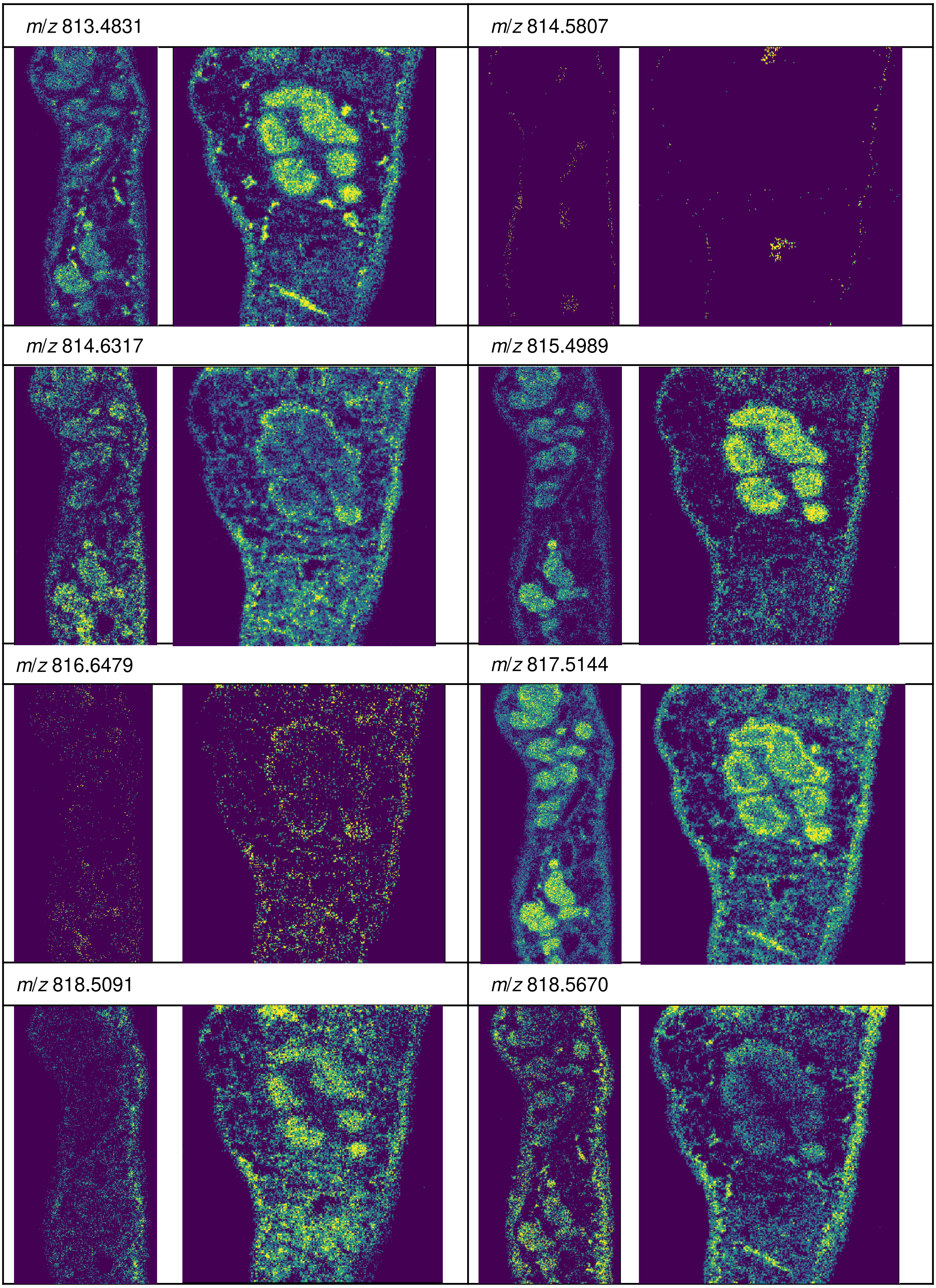
m/z 813.4831
m/z 814.5807
m/z 814.6317
m/z 815.4989
m/z 816.6479
m/z 817.5144
m/z 818.5091
m/z 818.5670

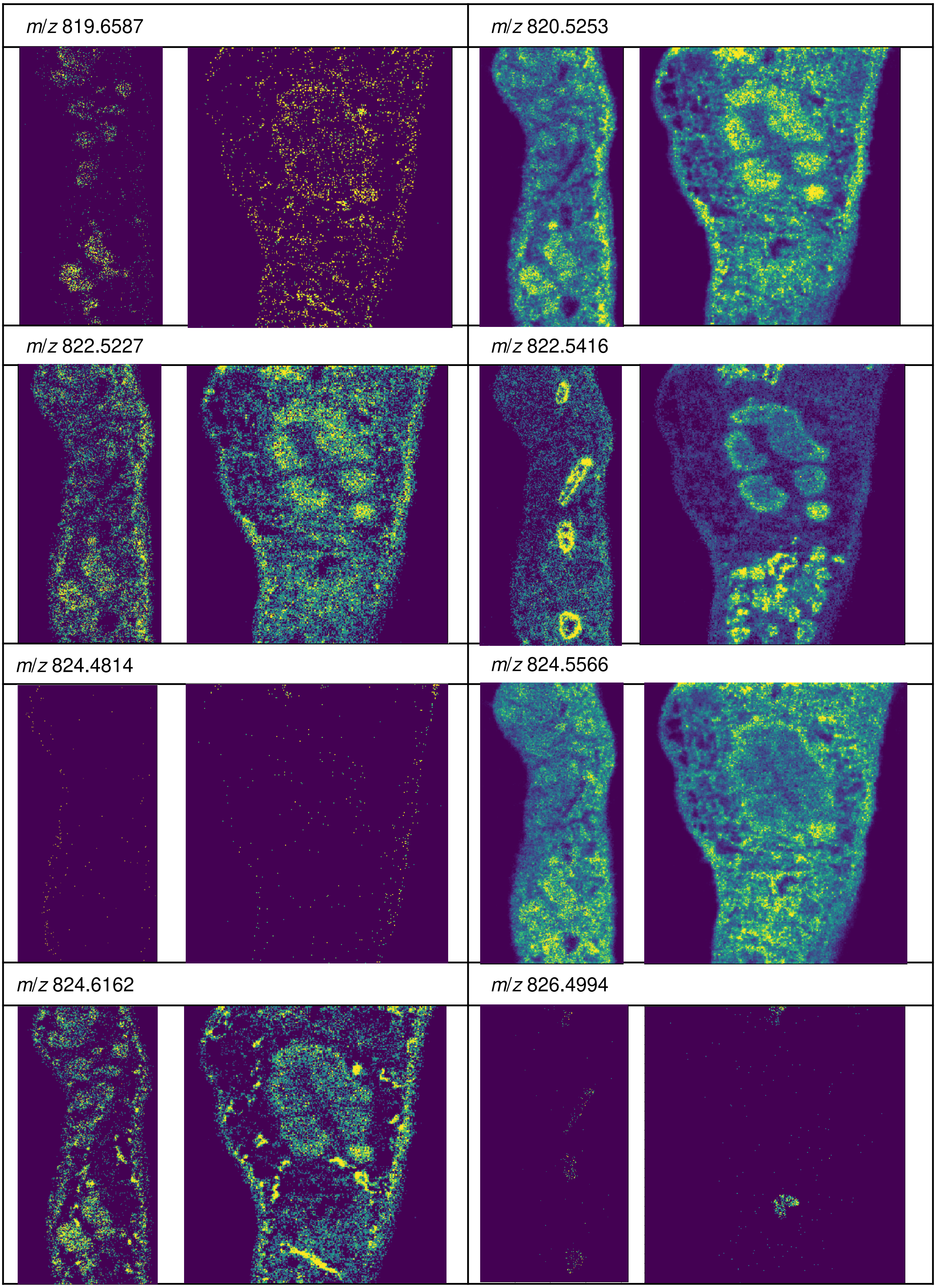
m/z 819.6587
m/z 820.5253
m/z 822.5227
m/z 822.5416
m/z 824.4814
m/z 824.5566
m/z 824.6162
m/z 826.4994

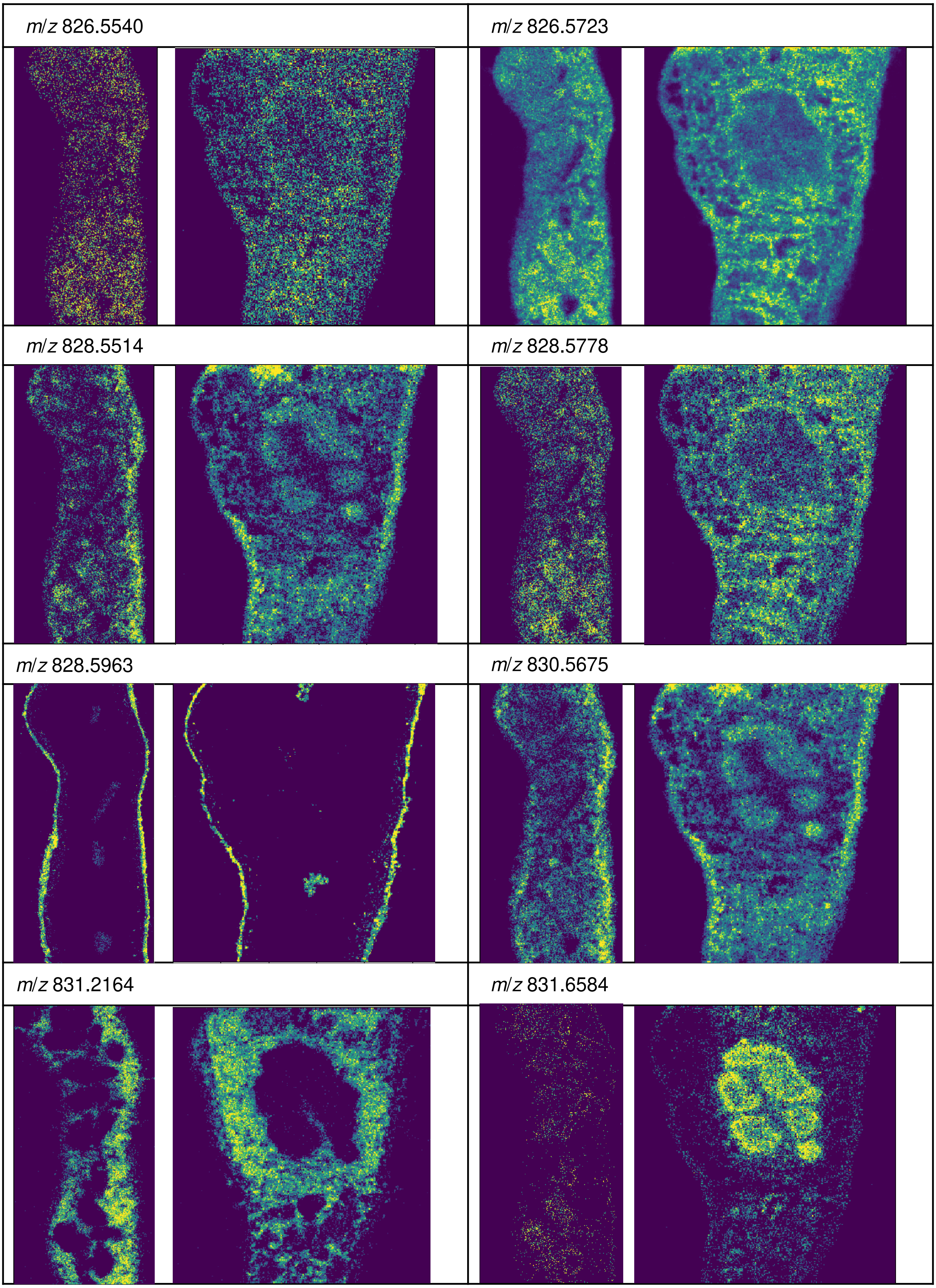
m/z 826.5540
m/z 826.5723
m/z 828.5514
m/z 828.5778
m/z 828.5963
m/z 830.5675
m/z 831.2164
m/z 831.6584

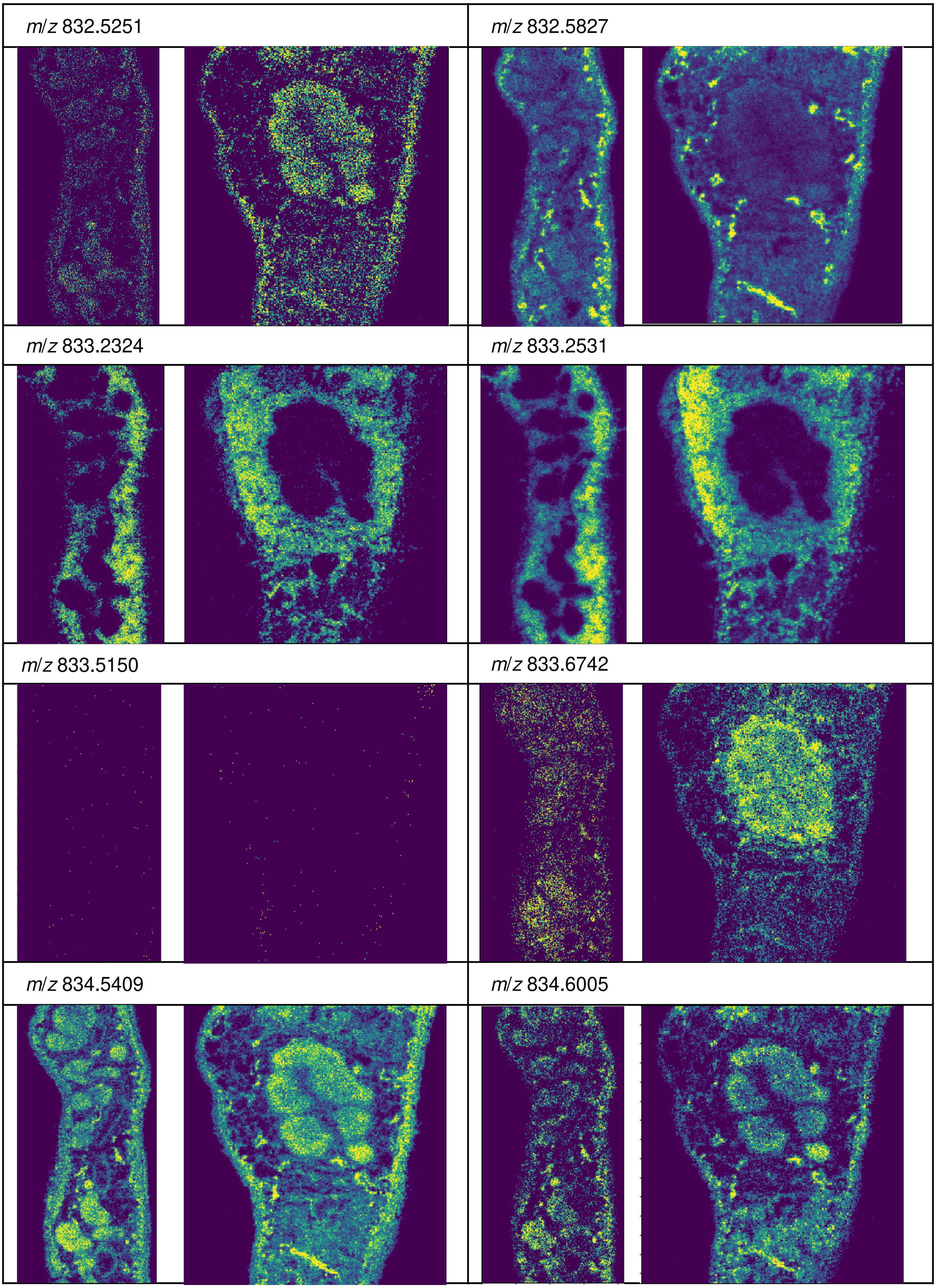

m/z 832.5251
m/z 832.5827
m/z 833.2324
m/z 833.2531
m/z 833.5150
m/z 833.6742
m/z 834.5409
m/z 834.6005

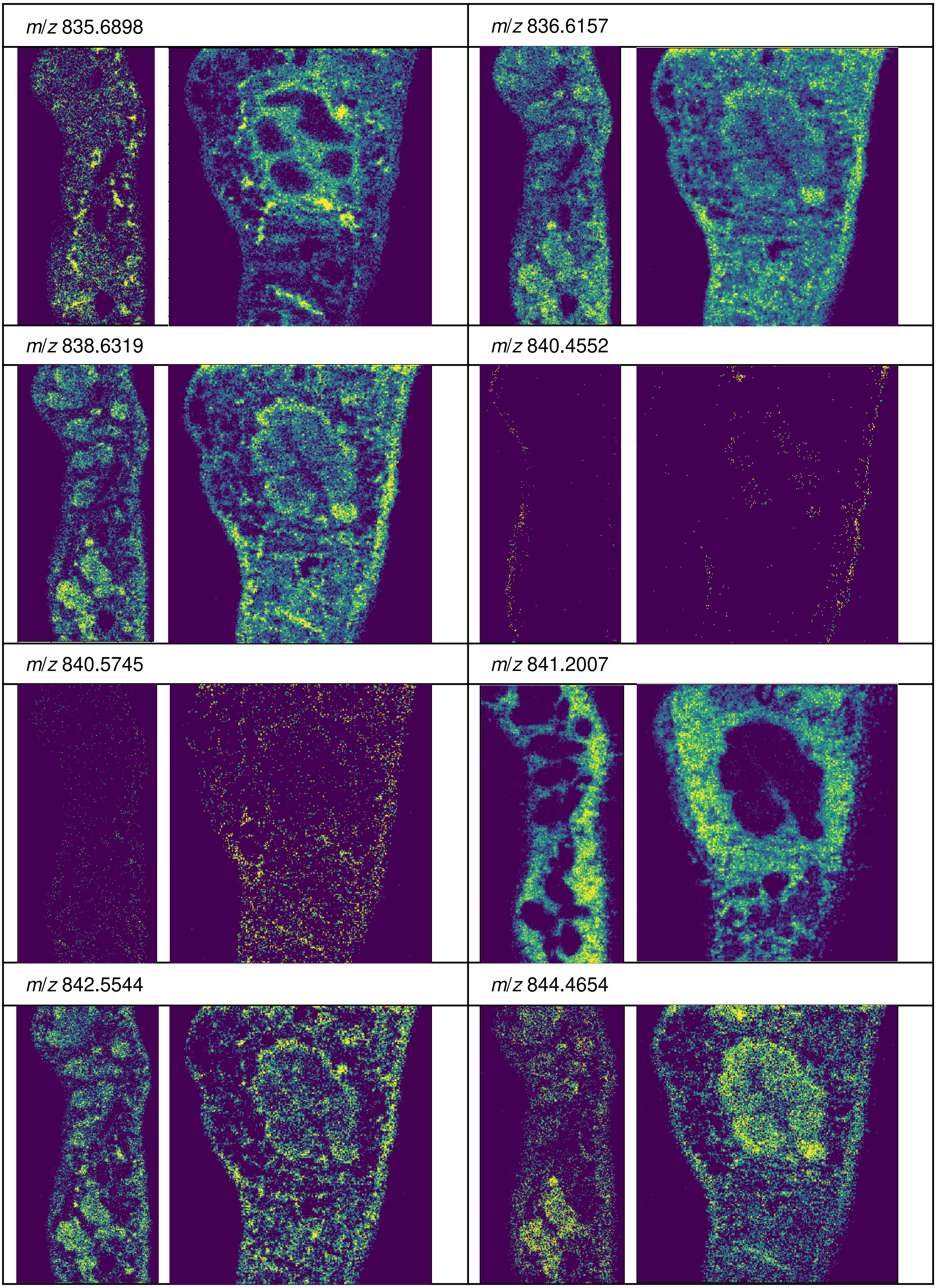
m/z 835.6898
m/z 836.6157
m/z 838.6319
m/z 840.4552
m/z 840.5745
m/z 841.2007
m/z 842.5544
m/z 844.4654

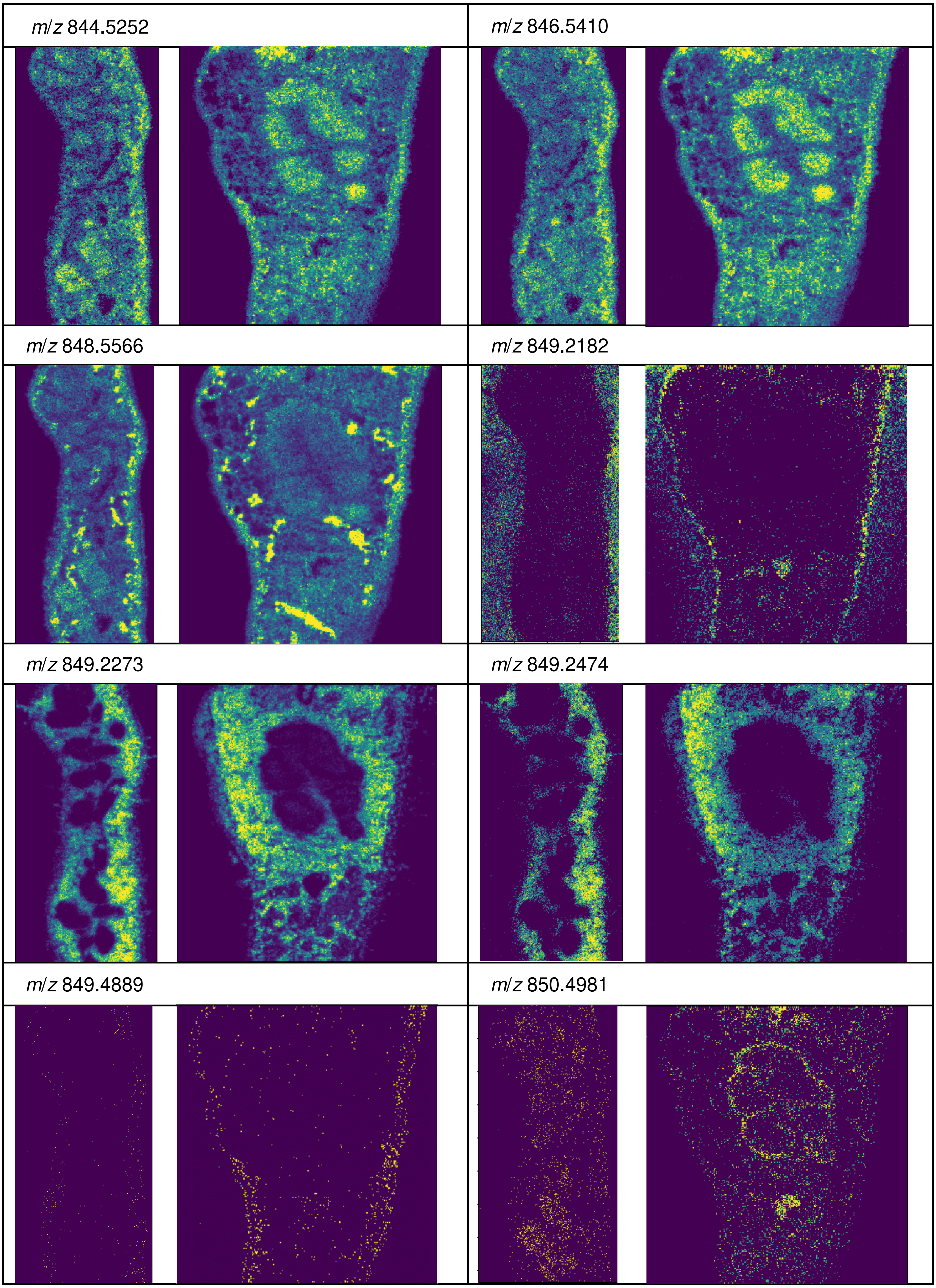

m/*z* 844.5252
m/*z* 846.5410
m/*z* 848.5566
m/*z* 849.2182
m/*z* 849.2273
m/*z* 849.2474
m/*z* 849.4889
m/*z* 850.4981

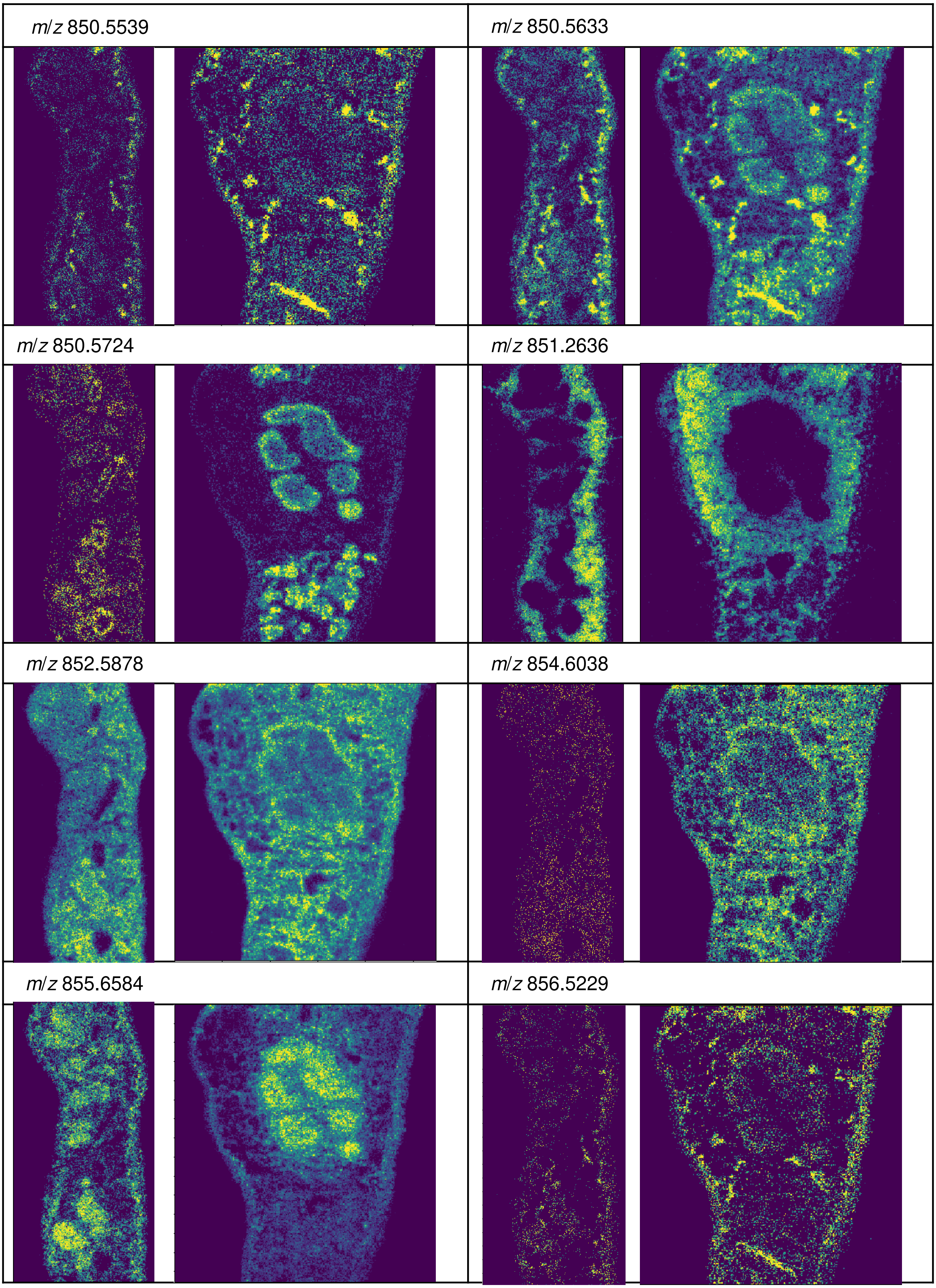
m/z 850.5539
m/z 850.5633
m/z 850.5724
m/z 851.2636
m/z 852.5878
m/z 854.6038
m/z 855.6584
m/z 856.5229

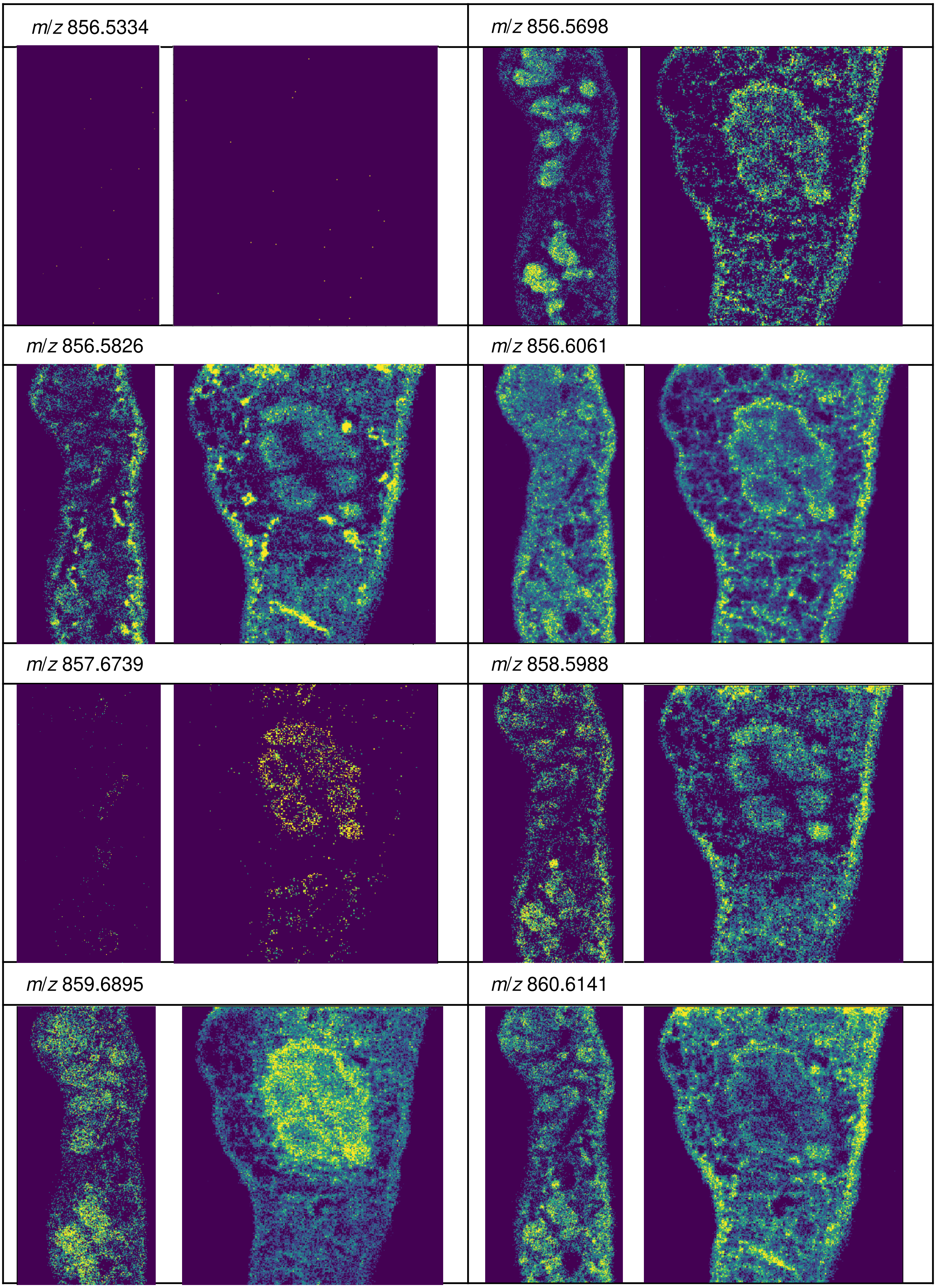

m/*z* 856.5334
m/*z* 856.5698
m/*z* 856.5826
m/*z* 856.6061
m/*z* 857.6739
m/*z* 858.5988
m/*z* 859.6895
m/*z* 860.6141

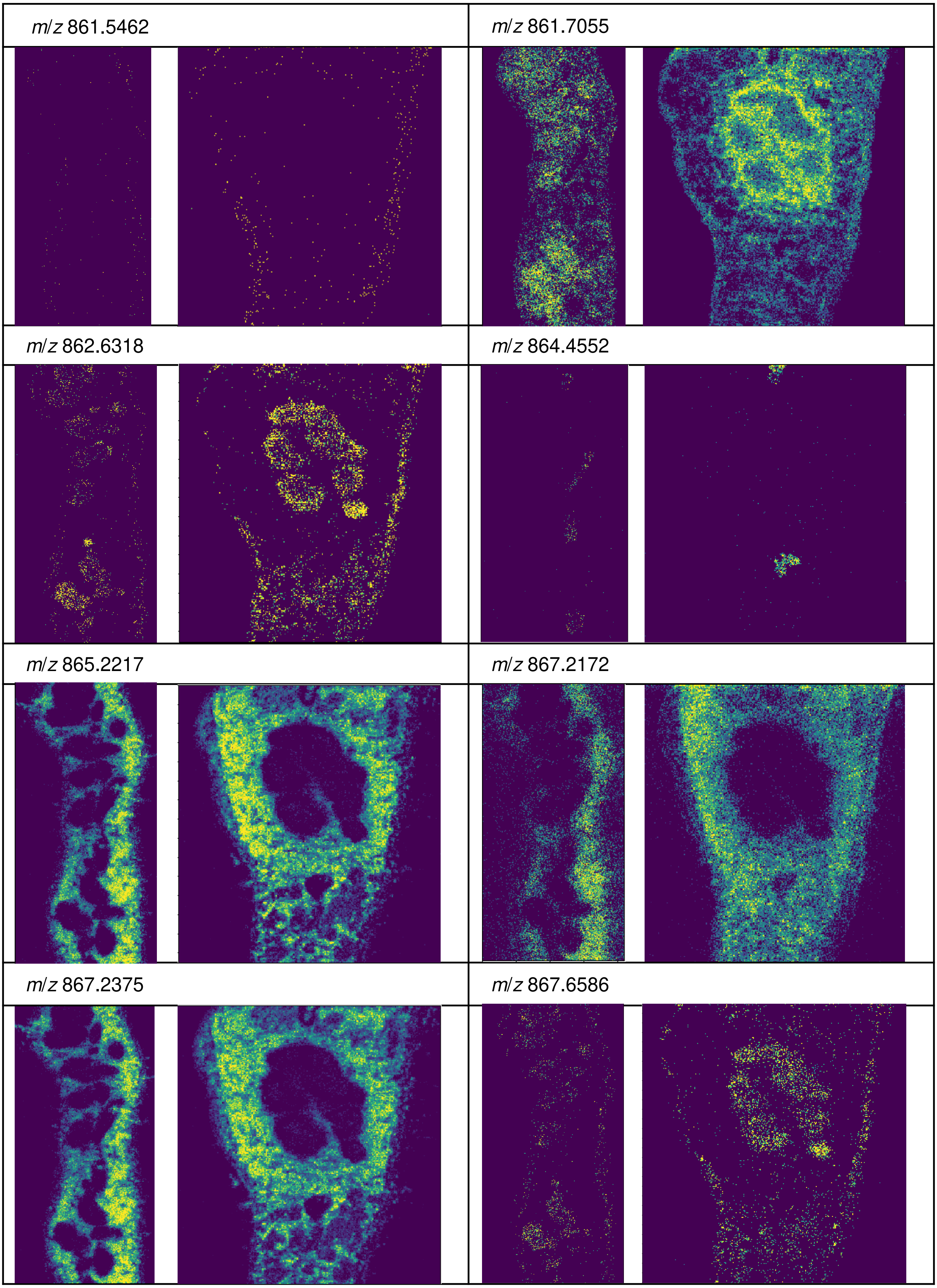
m/z 861.5462
m/z 861.7055
m/z 862.6318
m/z 864.4552
m/z 865.2217
m/z 867.2172
m/z 867.2375
m/z 867.6586

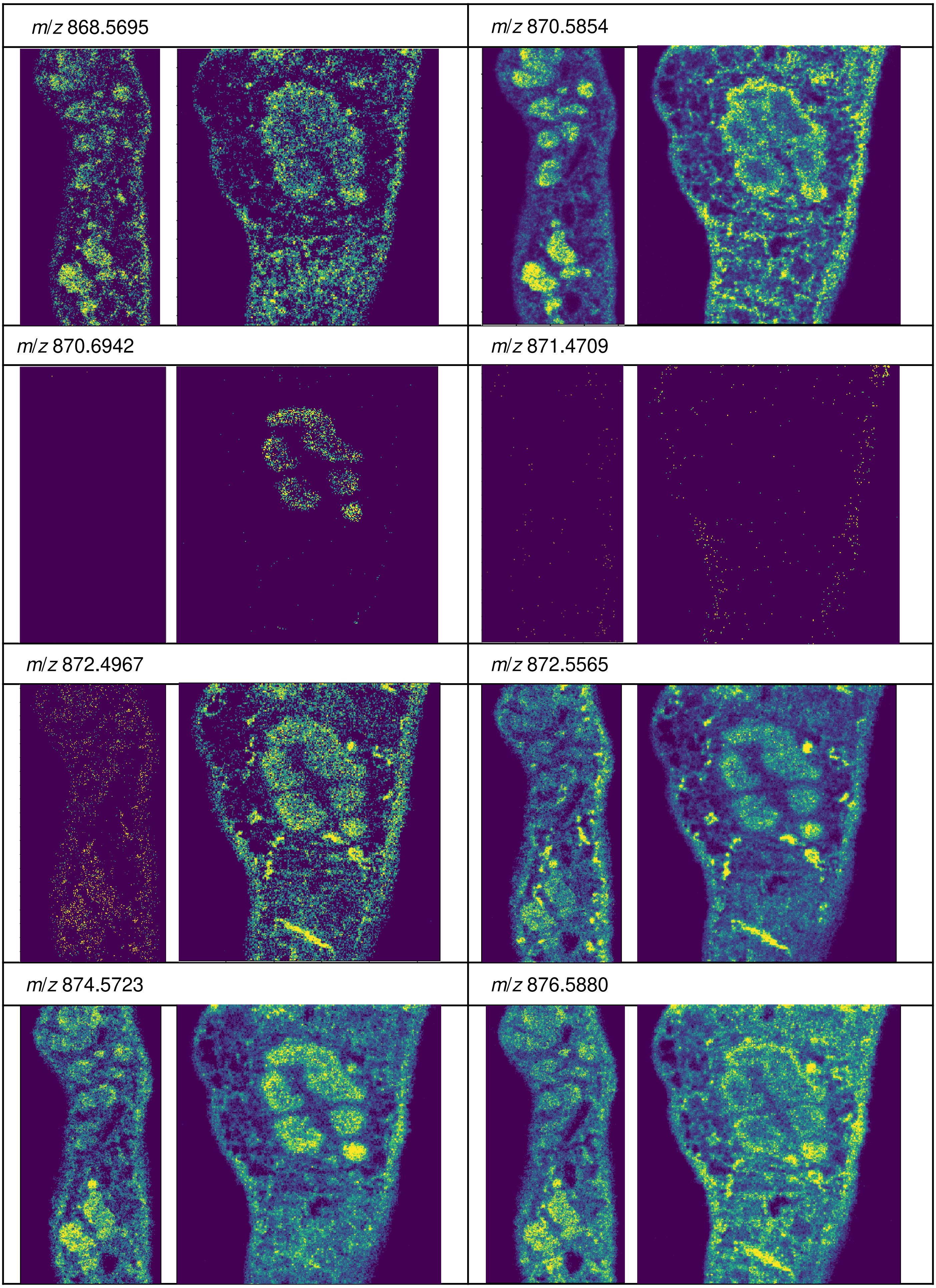

m/z 868.5695
m/z 870.5854
m/z 870.6942
m/z 871.4709
m/z 872.4967
m/z 872.5565
m/z 874.5723
m/z 876.5880

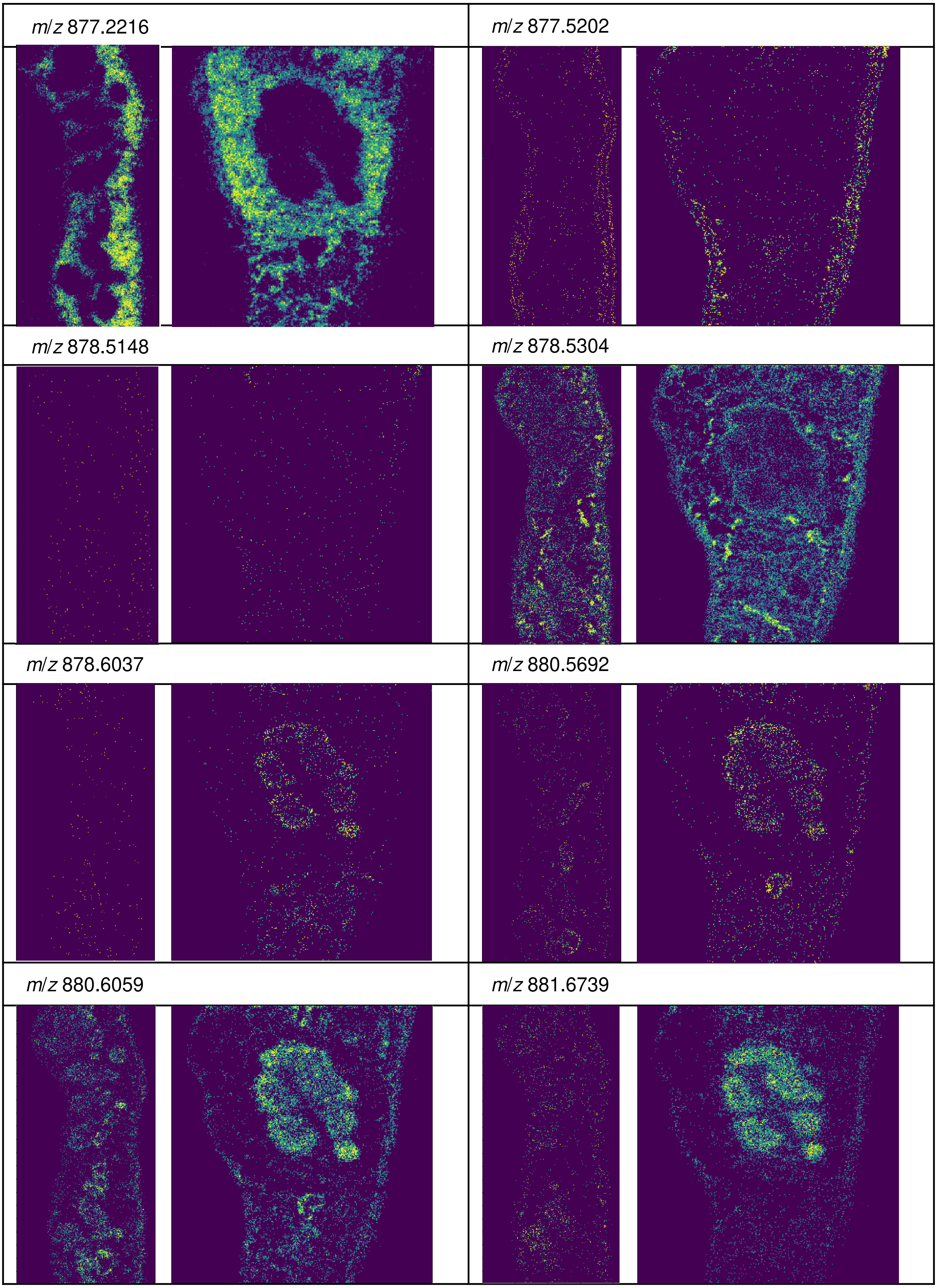

m/z 877.2216
m/z 877.5202
m/z 878.5148
m/z 878.5304
m/z 878.6037
m/z 880.5692
m/z 880.6059
m/z 881.6739

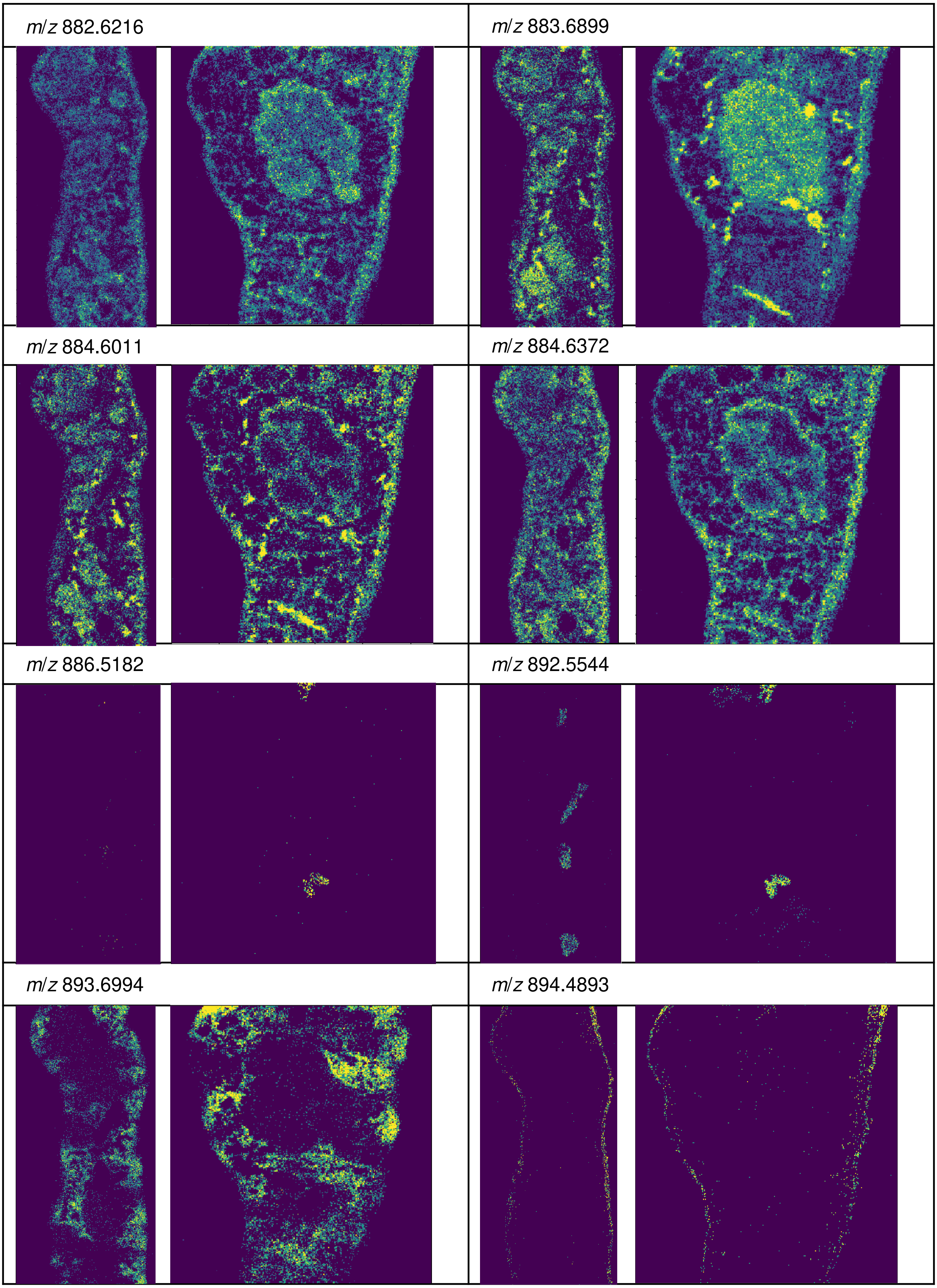
m/z 882.6216
m/z 883.6899
m/z 884.6011
m/z 884.6372
m/z 886.5182
m/z 892.5544
m/z 893.6994
m/z 894.4893

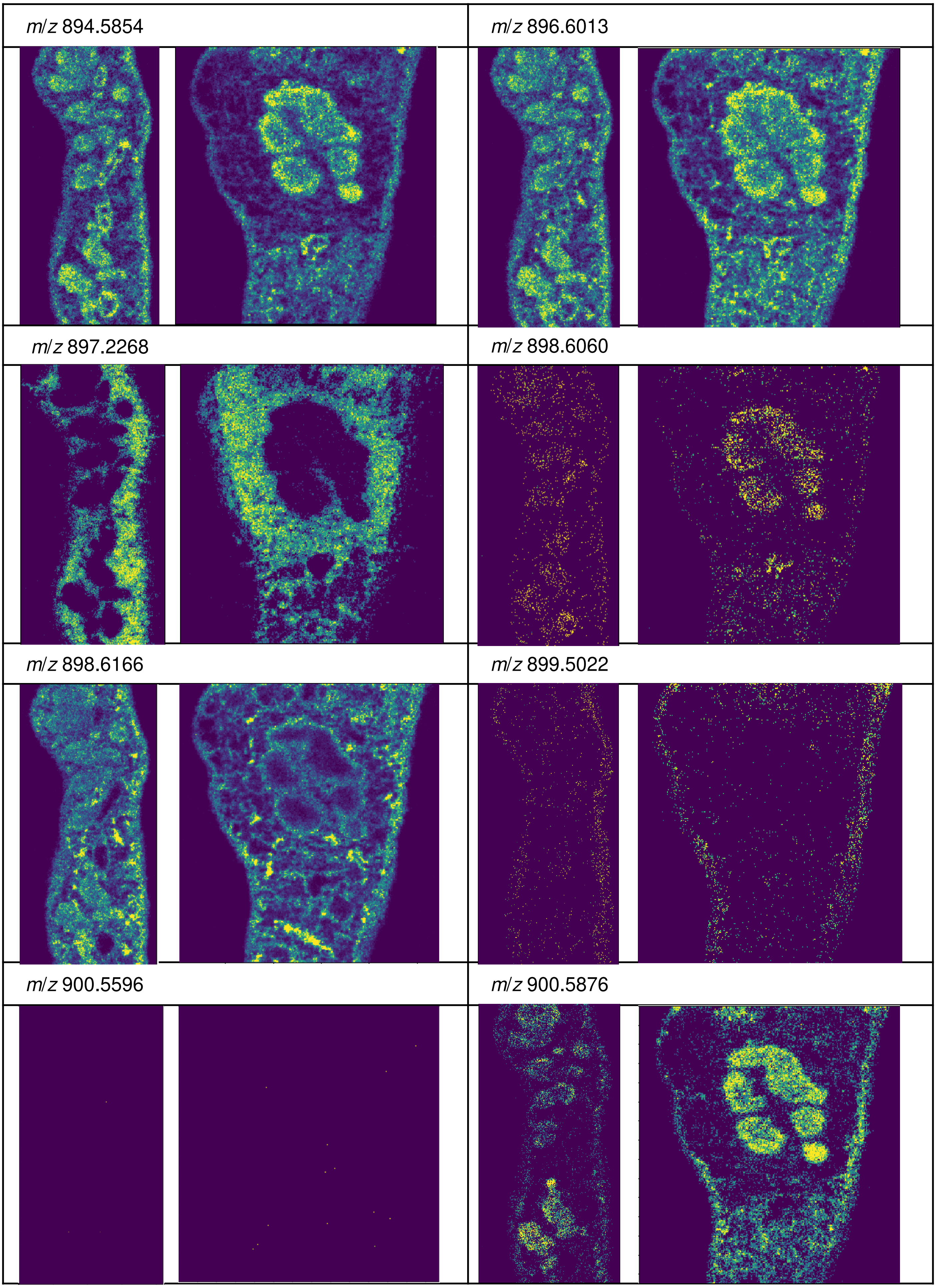

m/z 894.5854
m/z 896.6013
m/z 897.2268
m/z 898.6060
m/z 898.6166
m/z 899.5022
m/z 900.5596
m/z 900.5876

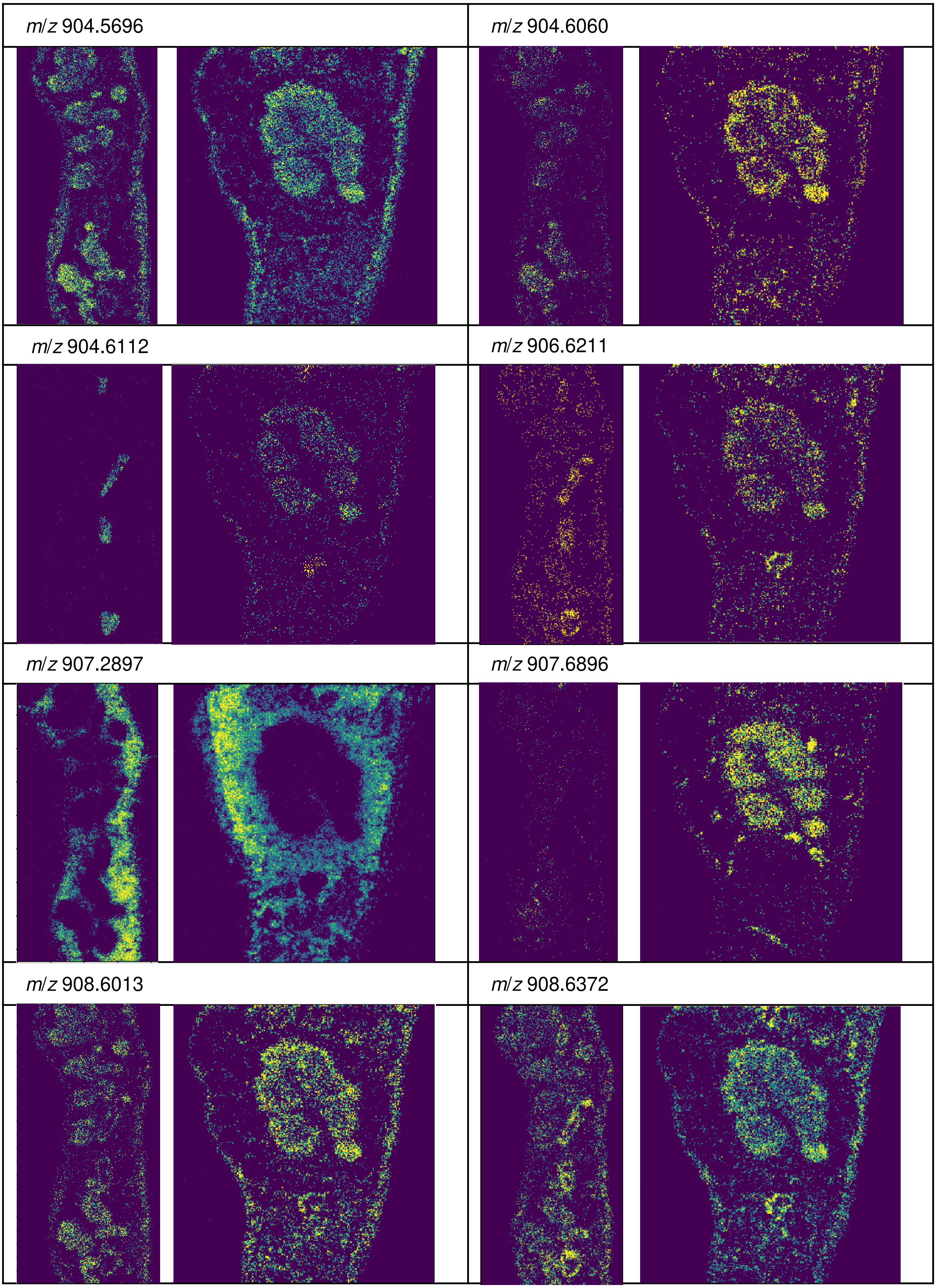
m/z 904.5696
m/z 904.6060
m/z 904.6112
m/z 906.6211
m/z 907.2897
m/z 907.6896
m/z 908.6013
m/z 908.6372

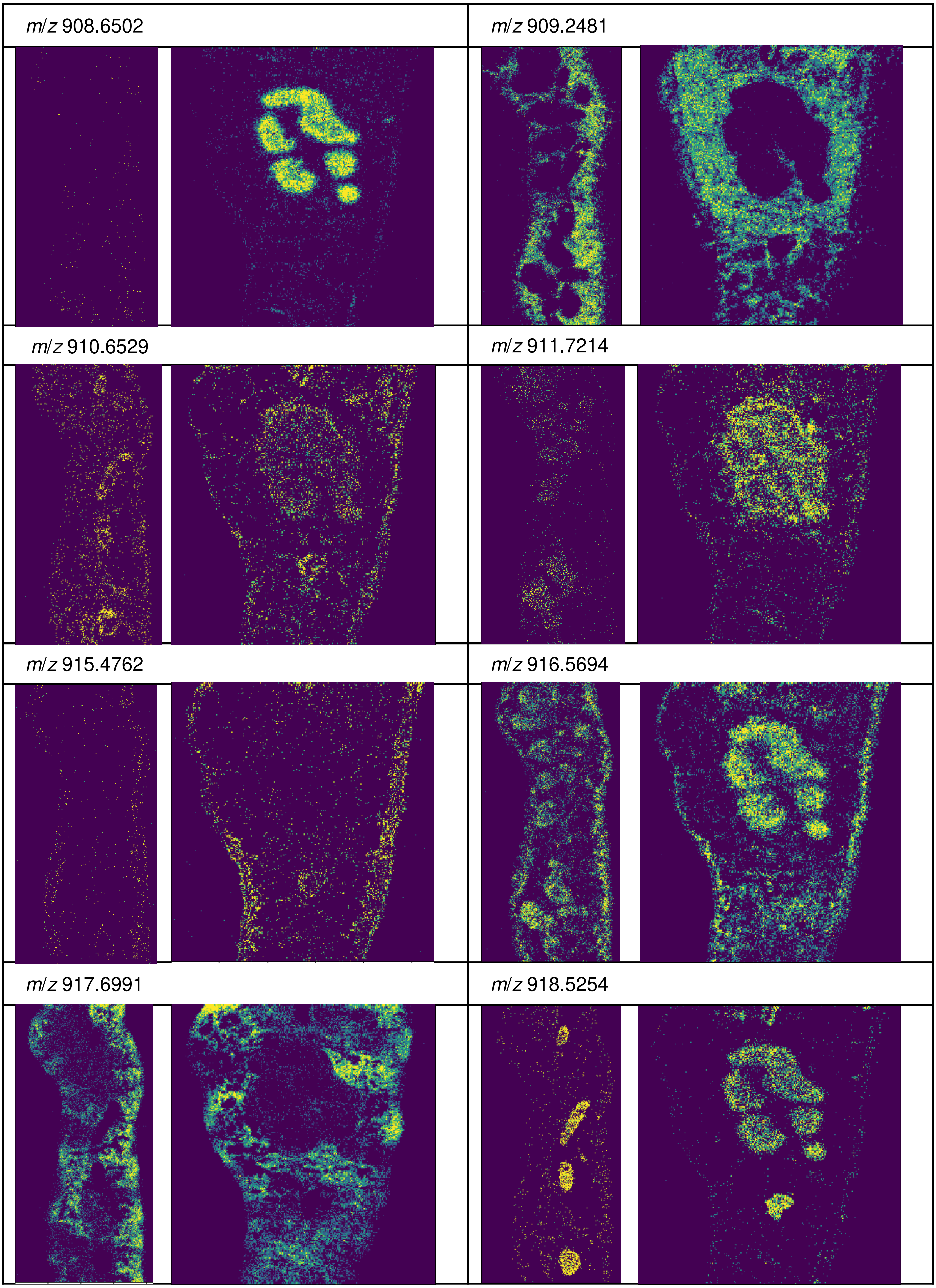

m/z 908.6502
m/z 909.2481
m/z 910.6529
m/z 911.7214
m/z 915.4762
m/z 916.5694
m/z 917.6991
m/z 918.5254

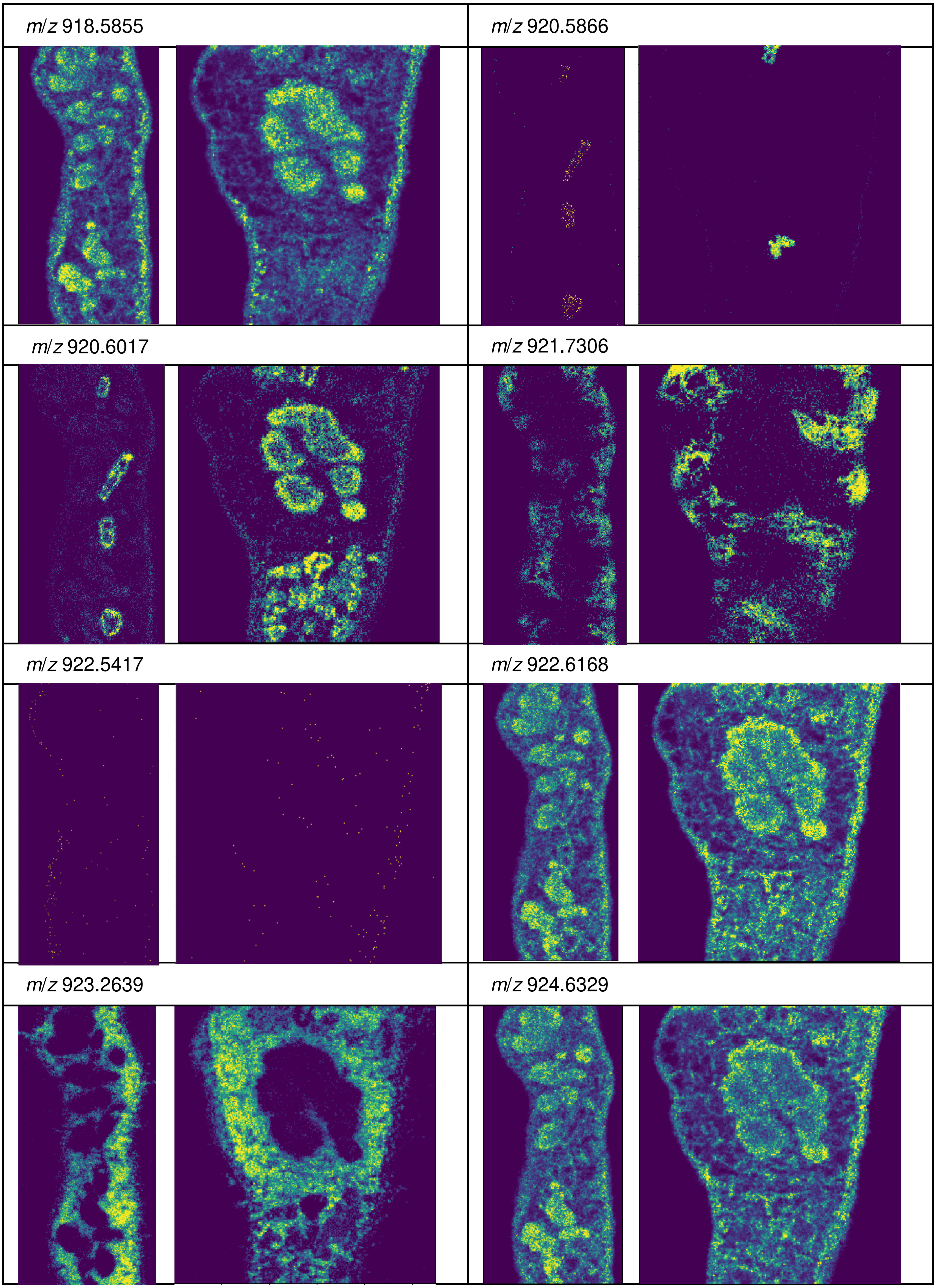
m/z 918.5855
m/z 920.5866
m/z 920.6017
m/z 921.7306
m/z 922.5417
m/z 922.6168
m/z 923.2639
m/z 924.6329

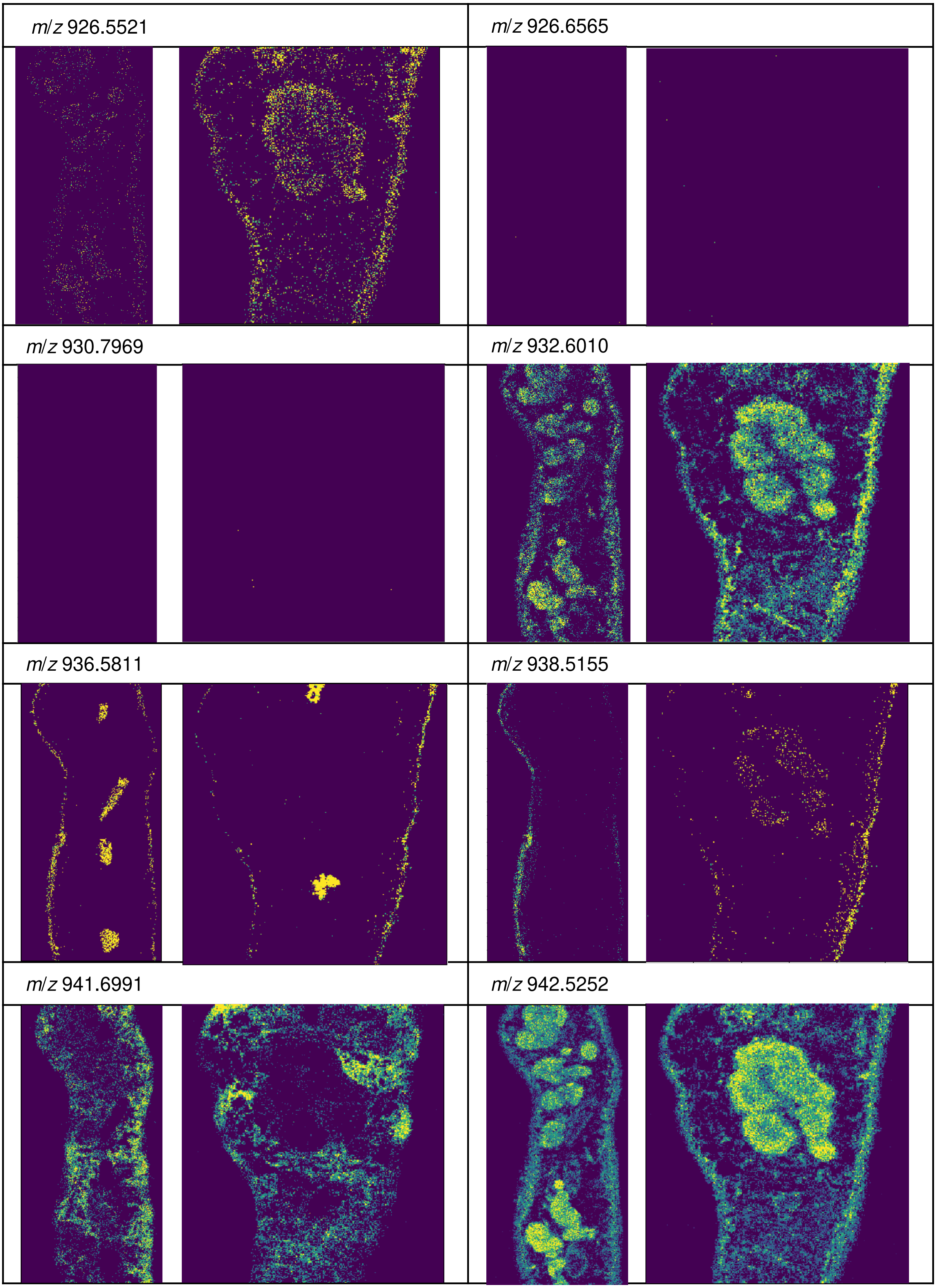
m/z 926.5521
m/z 926.6565
m/z 930.7969
m/z 932.6010
m/z 936.5811
m/z 938.5155
m/z 941.6991
m/z 942.5252

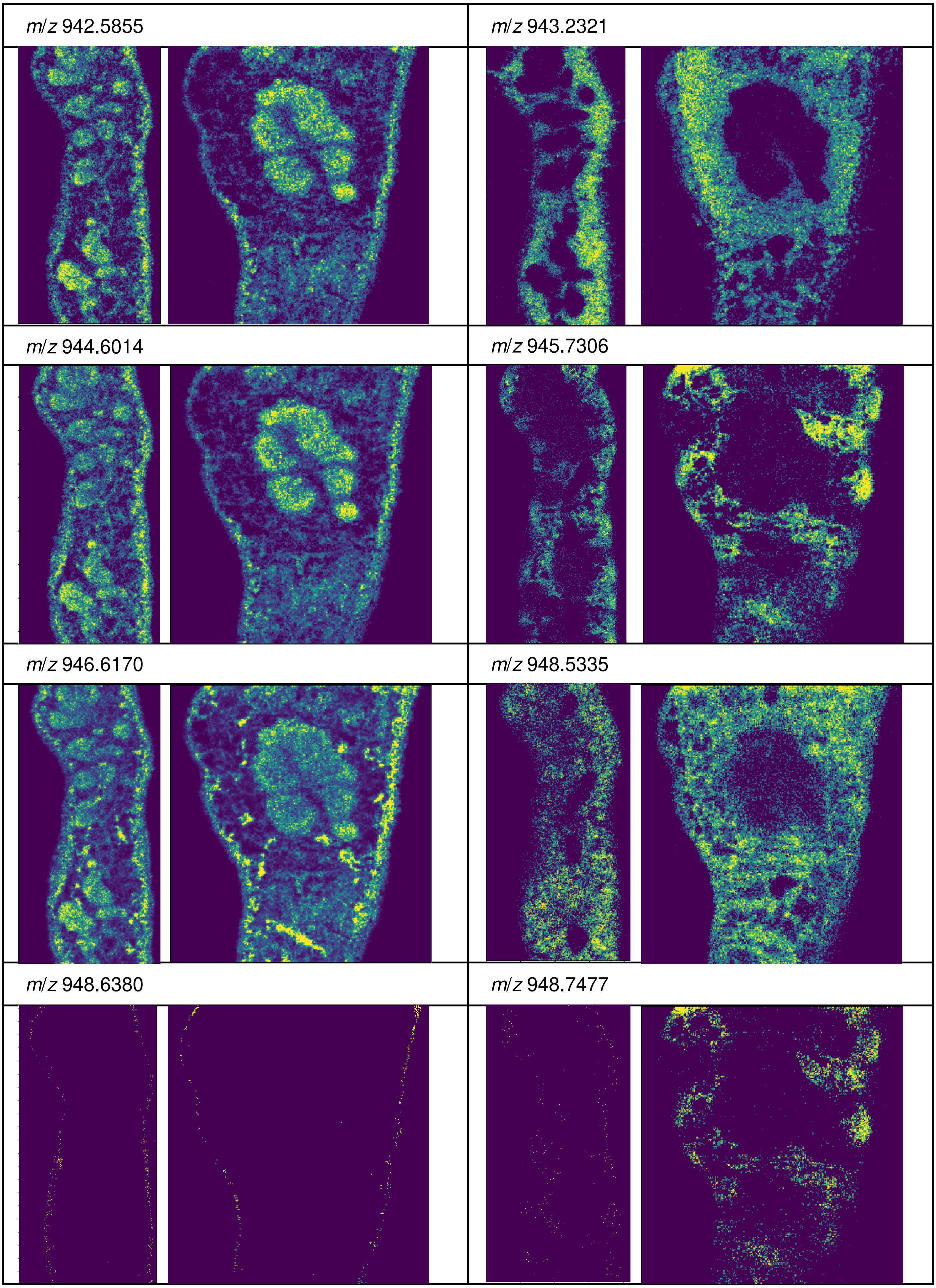
m/*z* 942.5855
m/*z* 943.2321
m/*z* 944.6014
m/*z* 945.7306
m/*z* 946.6170
m/*z* 948.5335
m/*z* 948.6380
m/*z* 948.7477

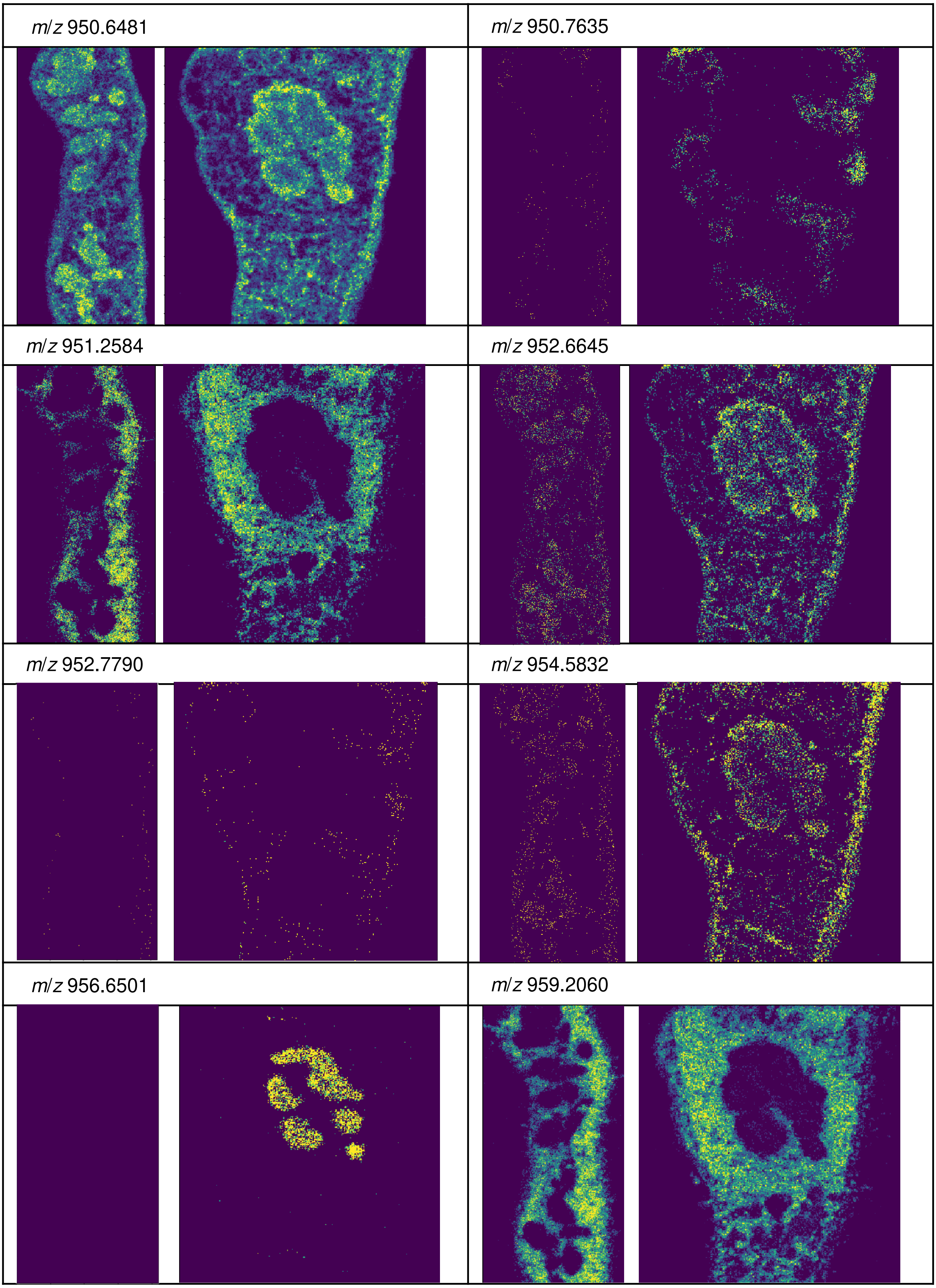
m/z 950.6481
m/z 950.7635
m/z 951.2584
m/z 952.6645
m/z 952.7790
m/z 954.5832
m/z 956.6501
m/z 959.2060

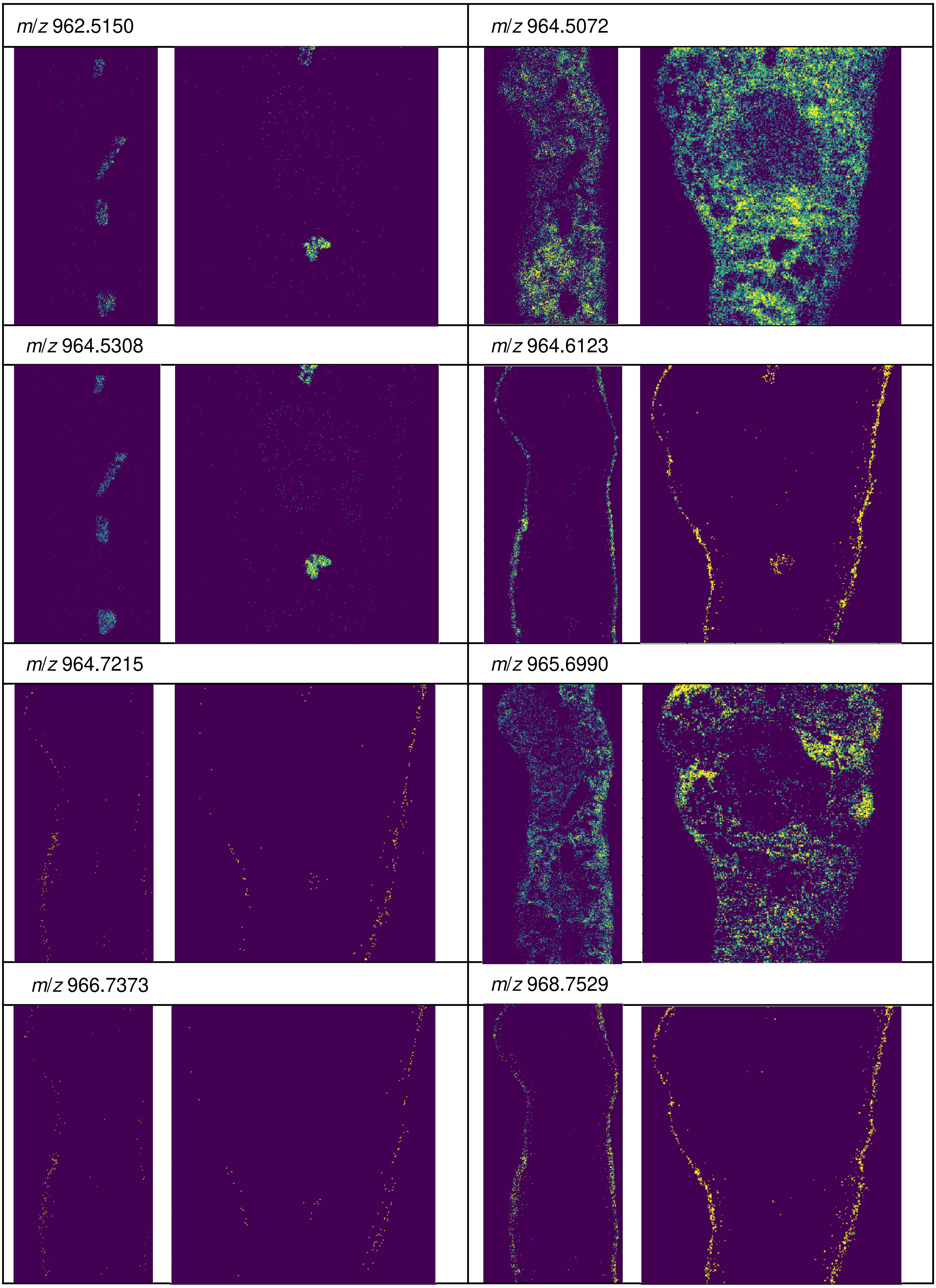
m/z 962.5150
m/z 964.5072
m/z 964.5308
m/z 964.6123
m/z 964.7215
m/z 965.6990
m/z 966.7373
m/z 968.7529

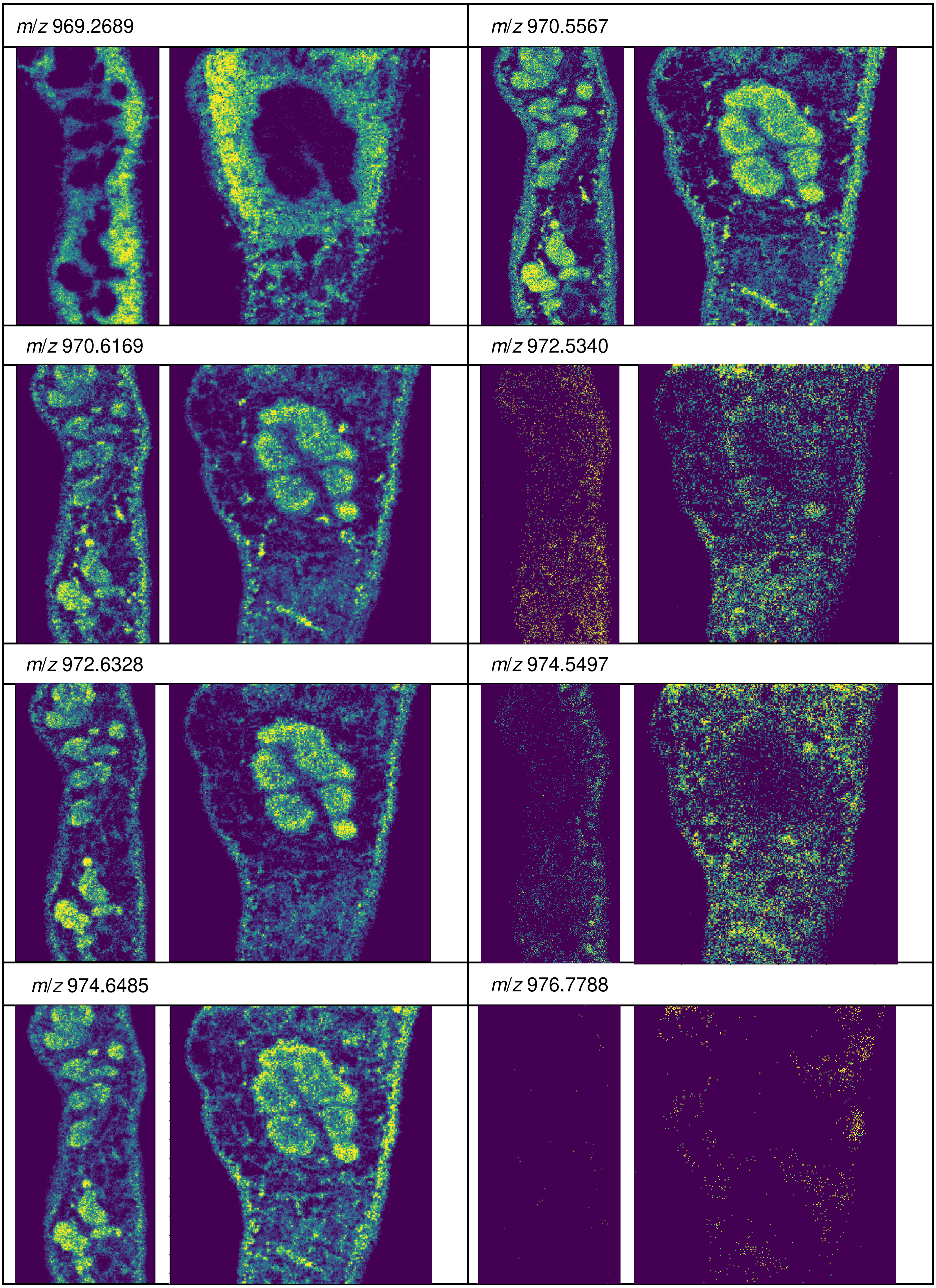
m/z 969.2689
m/z 970.5567
m/z 970.6169
m/z 972.5340
m/z 972.6328
m/z 974.5497
m/z 974.6485
m/z 976.7788

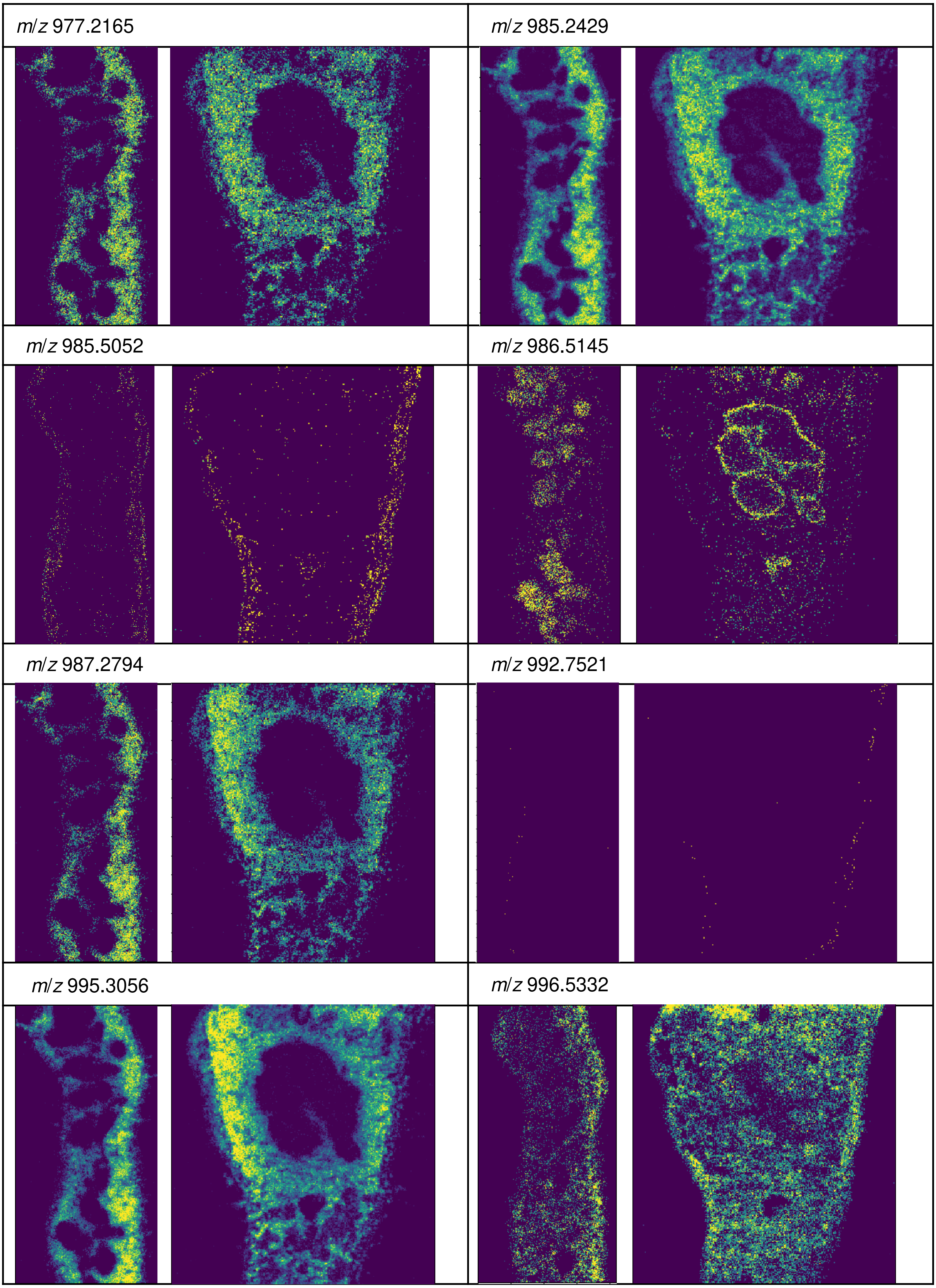

m/z 977.2165
m/z 985.2429
m/z 985.5052
m/z 986.5145
m/z 987.2794
m/z 992.7521
m/z 995.3056
m/z 996.5332

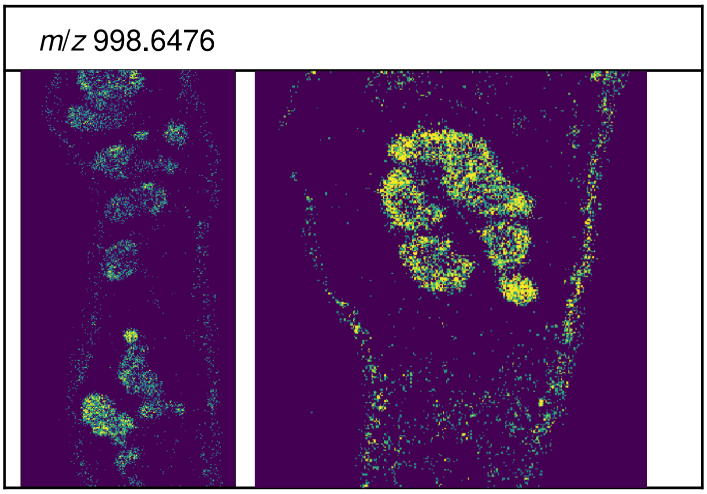
m/z 998.6476
