# Supplementary material for: Spatial lipidomics of Fasciola hepatica using high-resolution AP-SMALDI Orbitrap MSI – a lipid tissue atlas
Source: Front Cell Infect Microbiol. 2026 Oct 22;16:1934046. doi: 10.3389/fcimb.2026.1934046 (PMC13735547; doi:10.3389/fcimb.2026.1934046)
Supplement: Supplementary file 1 [file DataSheet1.zip › Supplementary images 2.pdf]

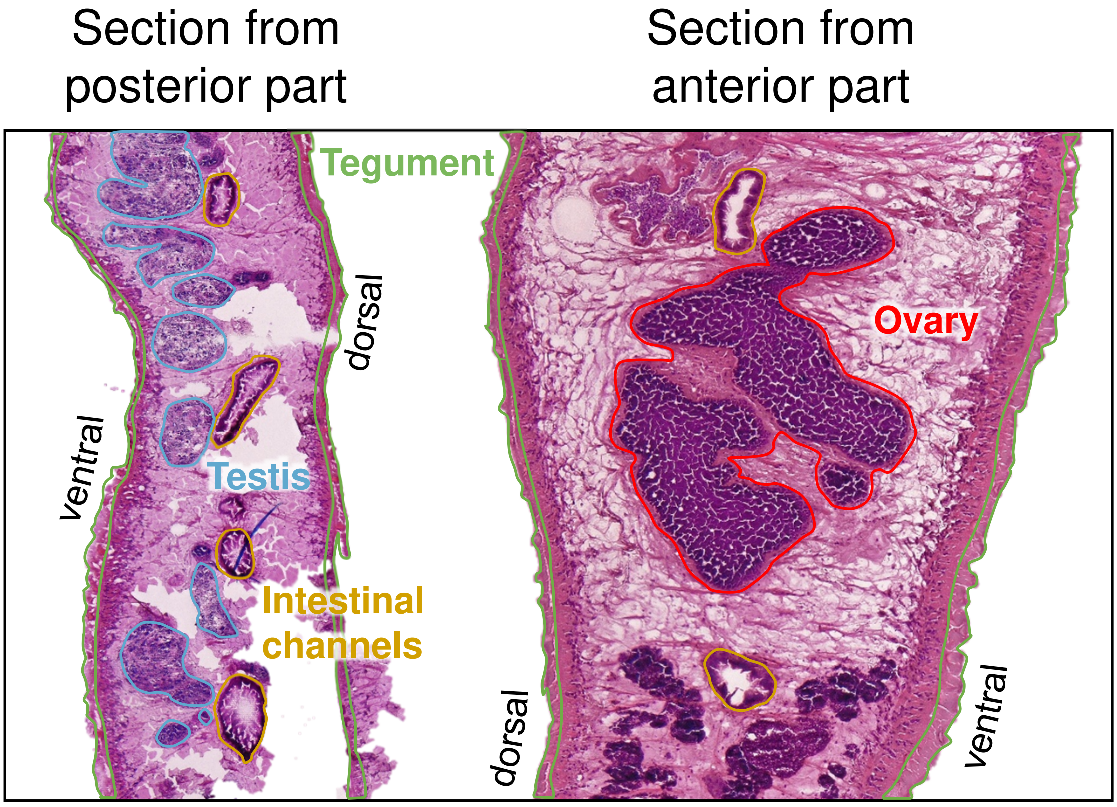
Section from posterior part
Section from anterior part
Tegument
dorsal
ventral
Testis
Intestinal channels
Ovary
dorsal
ventral

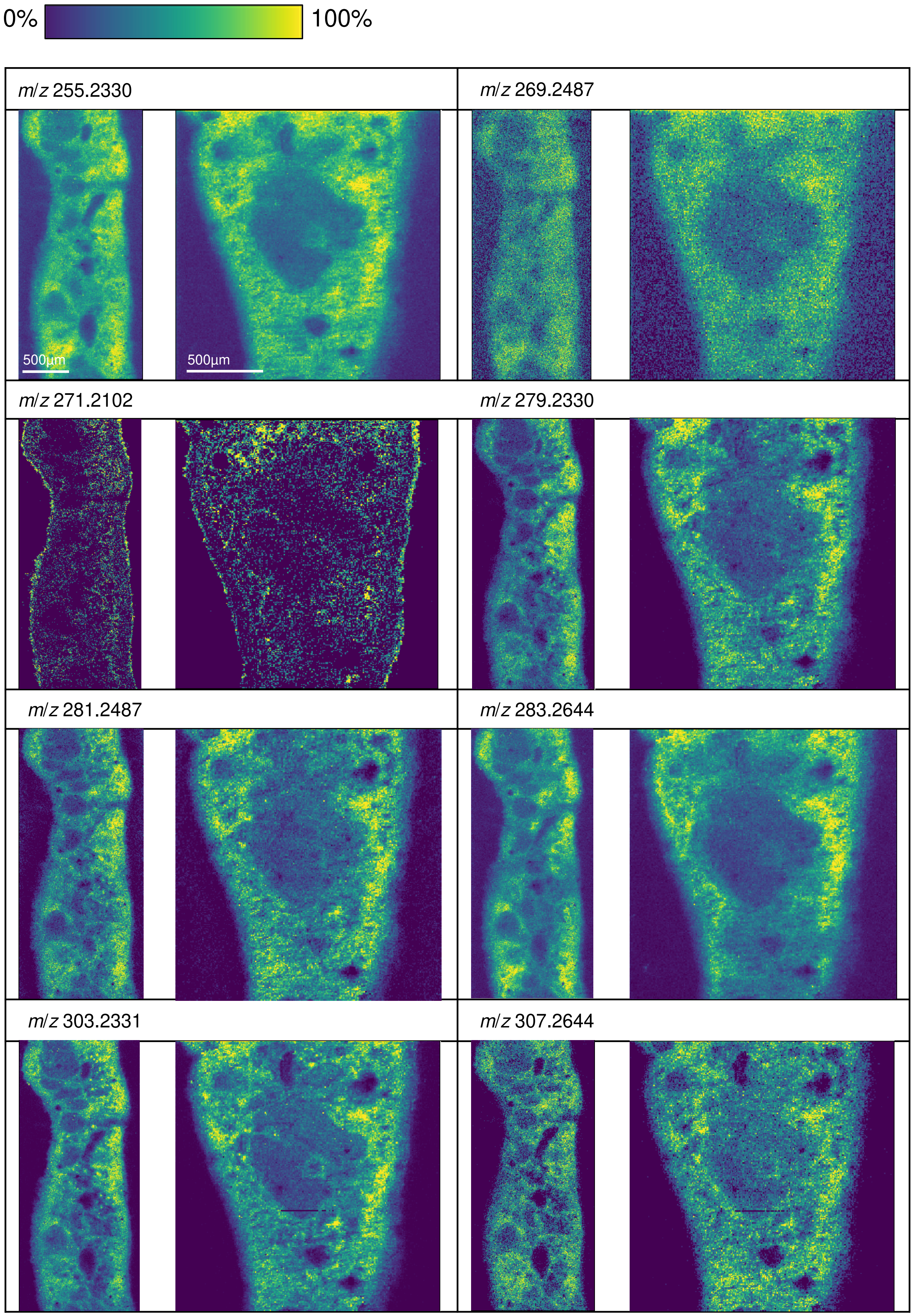
0%
100%
m/z 255.2330
500µm
500µm
m/z 269.2487
m/z 271.2102
m/z 279.2330
m/z 281.2487
m/z 283.2644
m/z 303.2331
m/z 307.2644

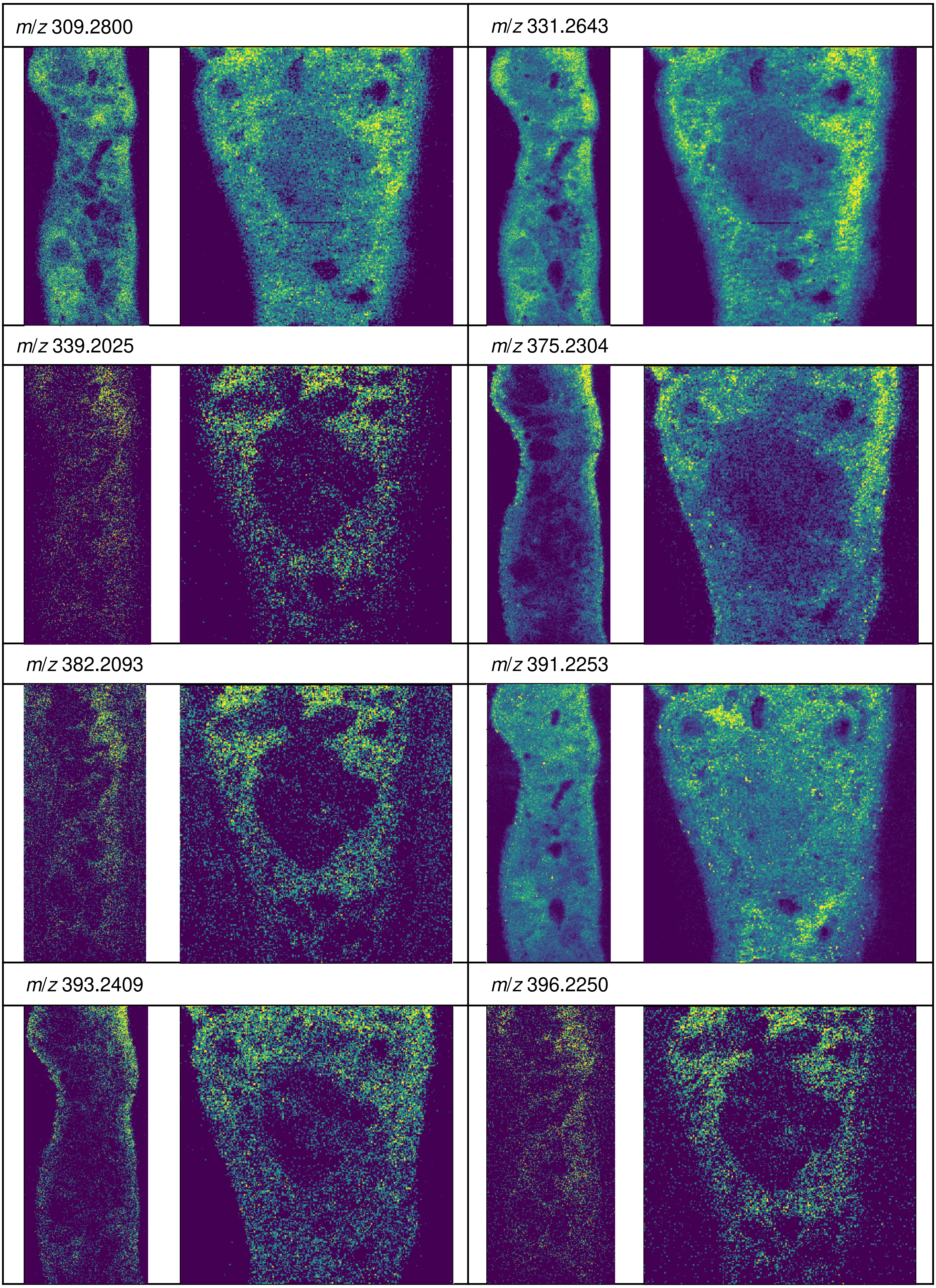

m/*z* 309.2800
m/*z* 331.2643
m/*z* 339.2025
m/*z* 375.2304
m/*z* 382.2093
m/*z* 391.2253
m/*z* 393.2409
m/*z* 396.2250

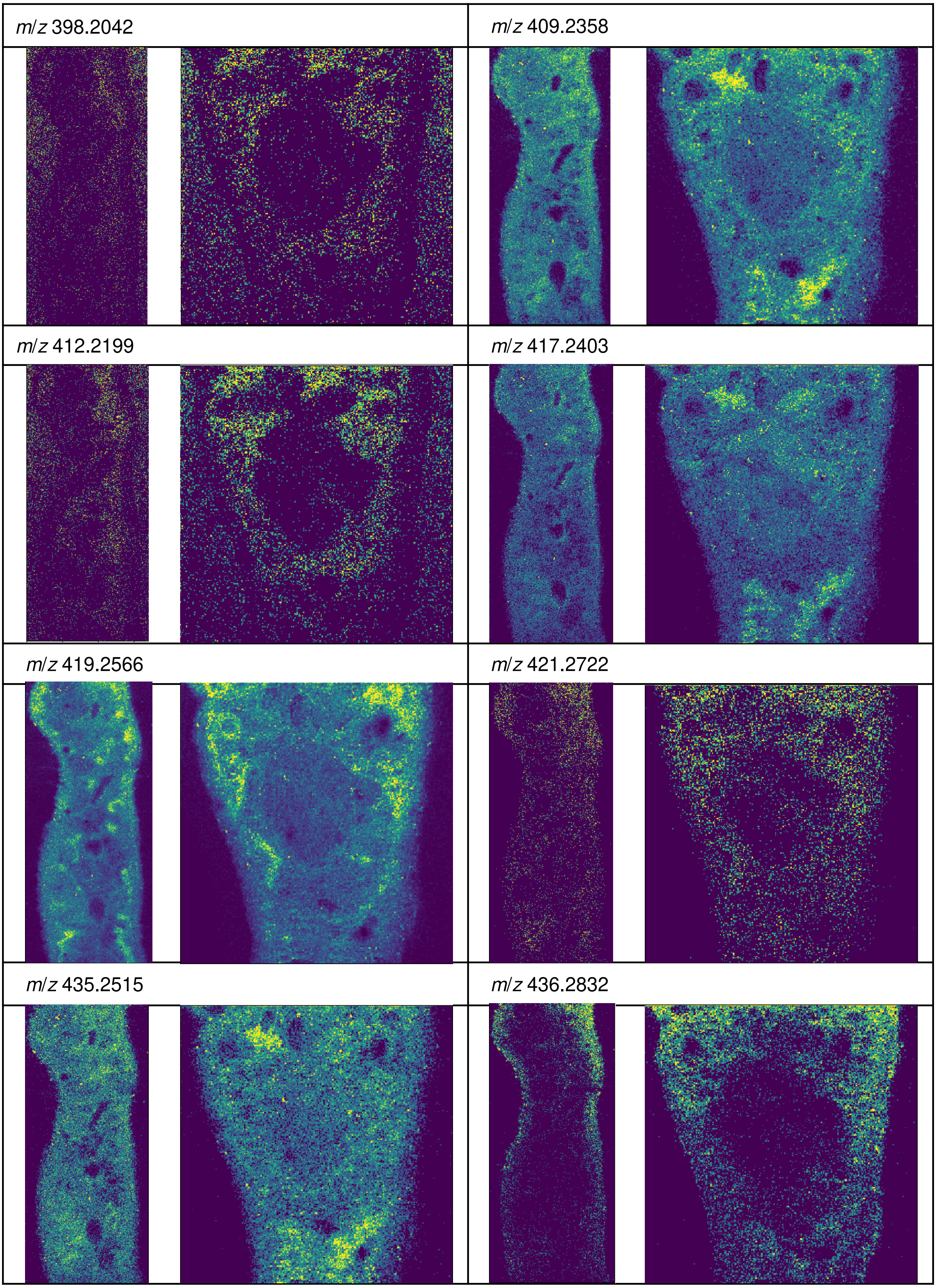

m/z 398.2042
m/z 409.2358
m/z 412.2199
m/z 417.2403
m/z 419.2566
m/z 421.2722
m/z 435.2515
m/z 436.2832

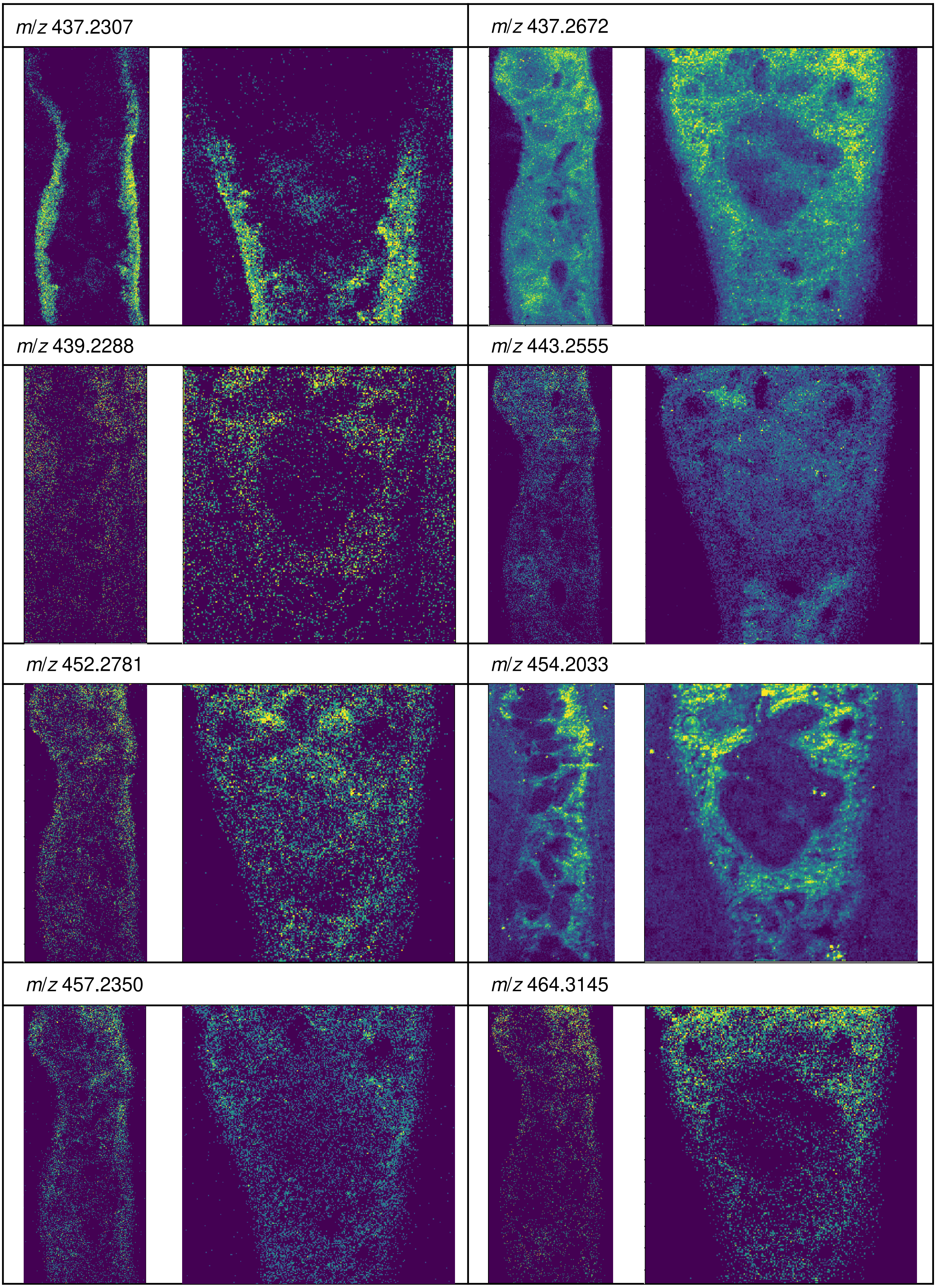
m/z 437.2307
m/z 437.2672
m/z 439.2288
m/z 443.2555
m/z 452.2781
m/z 454.2033
m/z 457.2350
m/z 464.3145

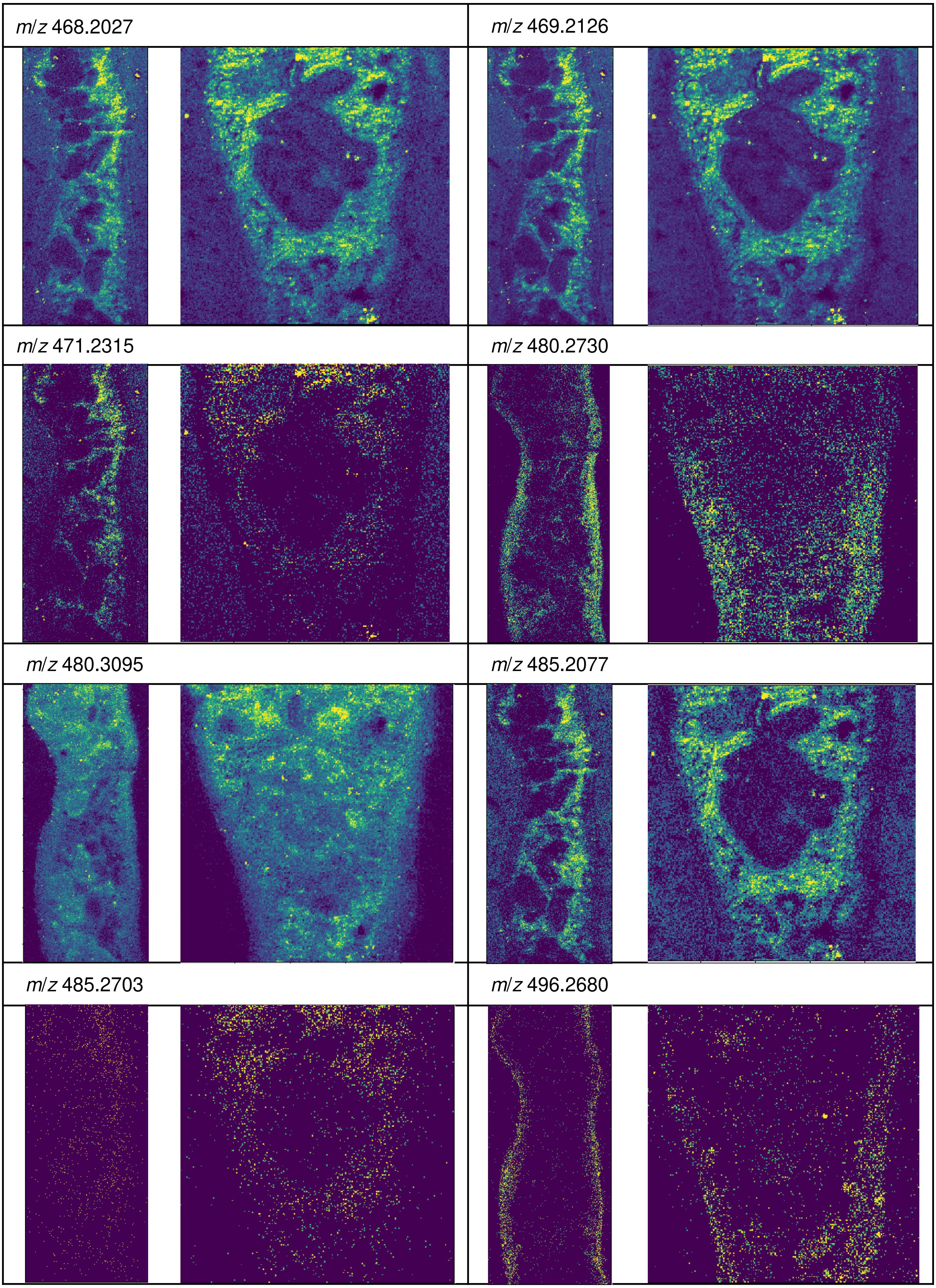
m/z 468.2027
m/z 469.2126
m/z 471.2315
m/z 480.2730
m/z 480.3095
m/z 485.2077
m/z 485.2703
m/z 496.2680

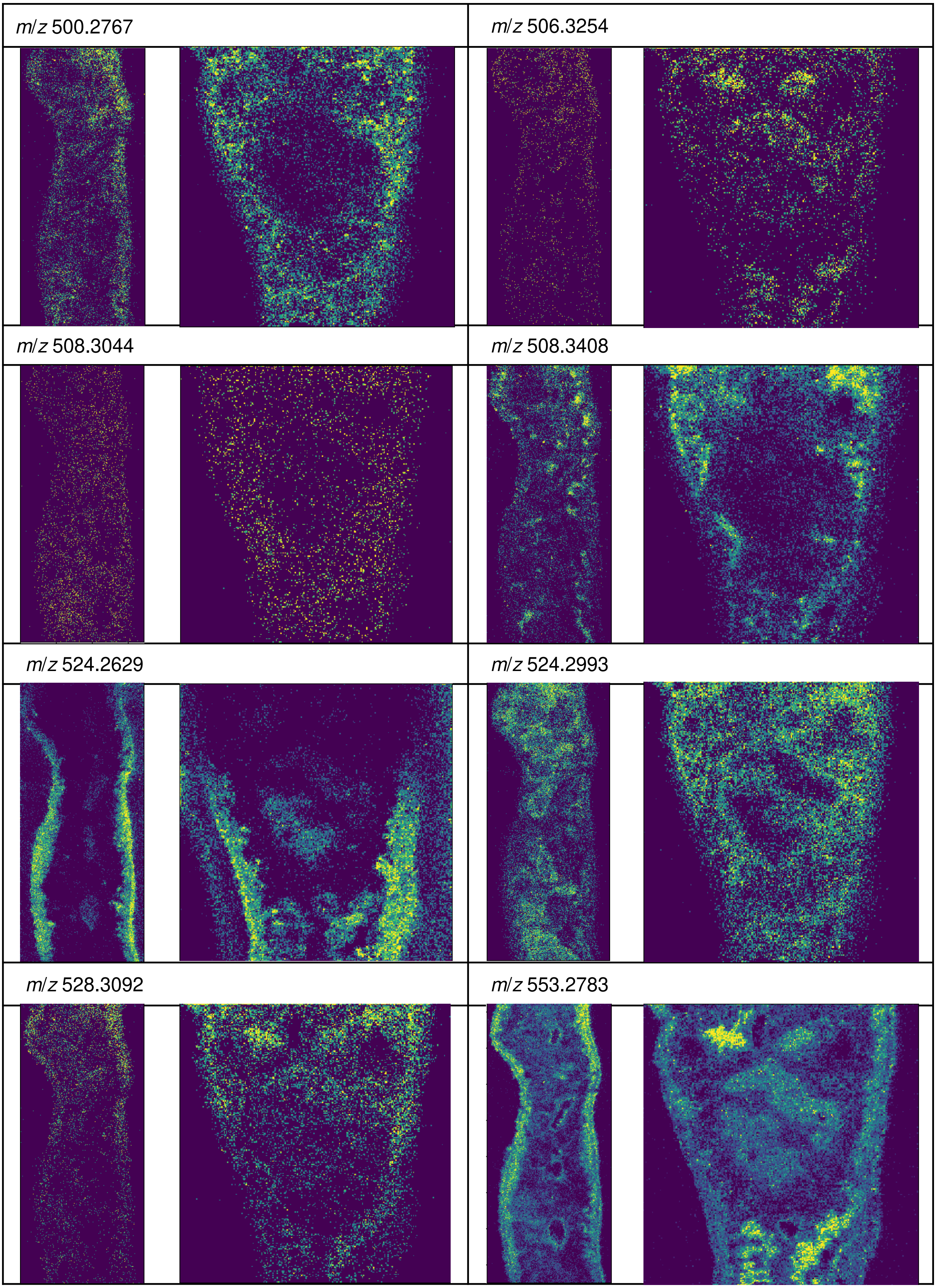
m/z 500.2767
m/z 506.3254
m/z 508.3044
m/z 508.3408
m/z 524.2629
m/z 524.2993
m/z 528.3092
m/z 553.2783

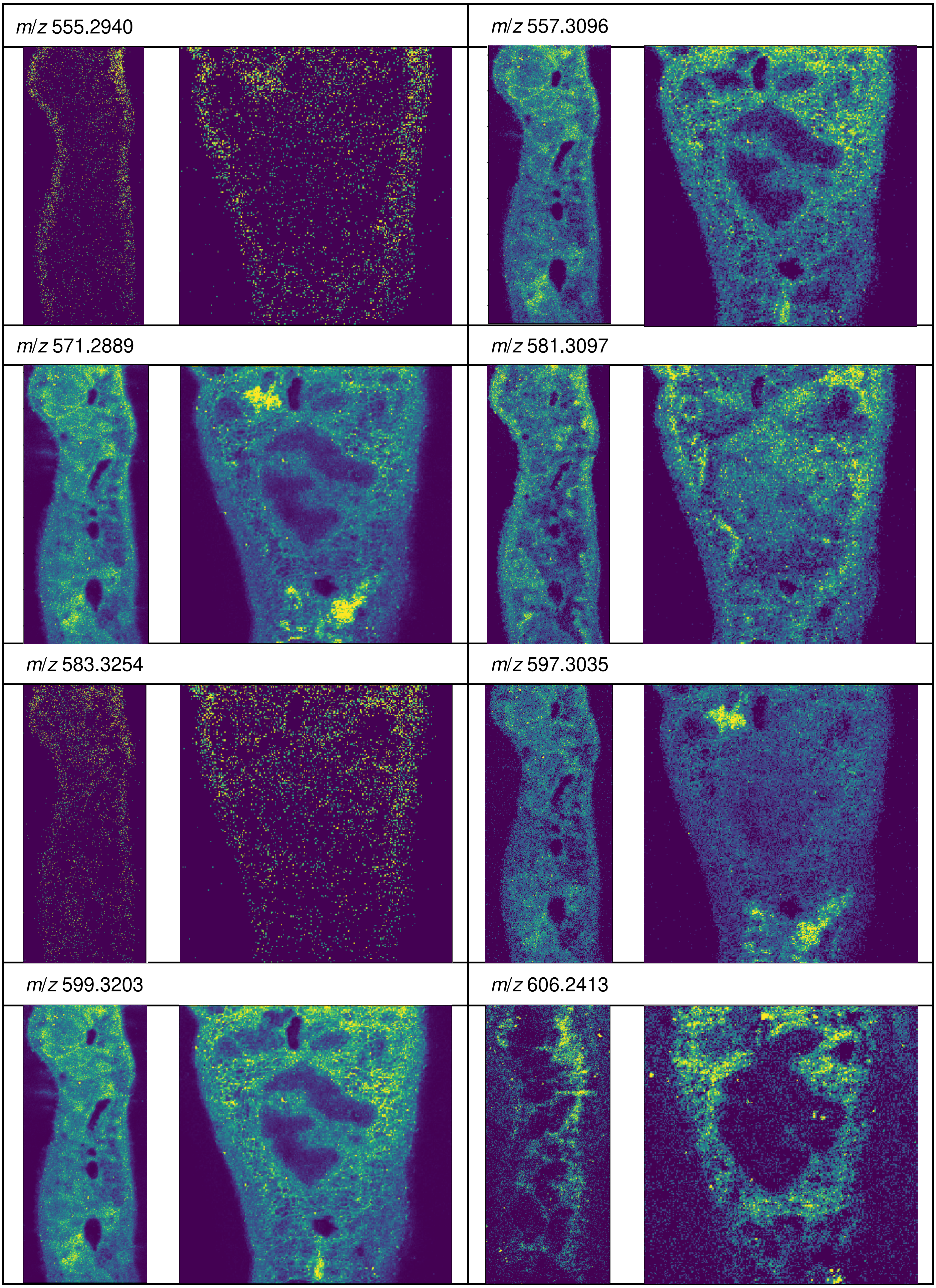
m/z 555.2940
m/z 557.3096
m/z 571.2889
m/z 581.3097
m/z 583.3254
m/z 597.3035
m/z 599.3203
m/z 606.2413

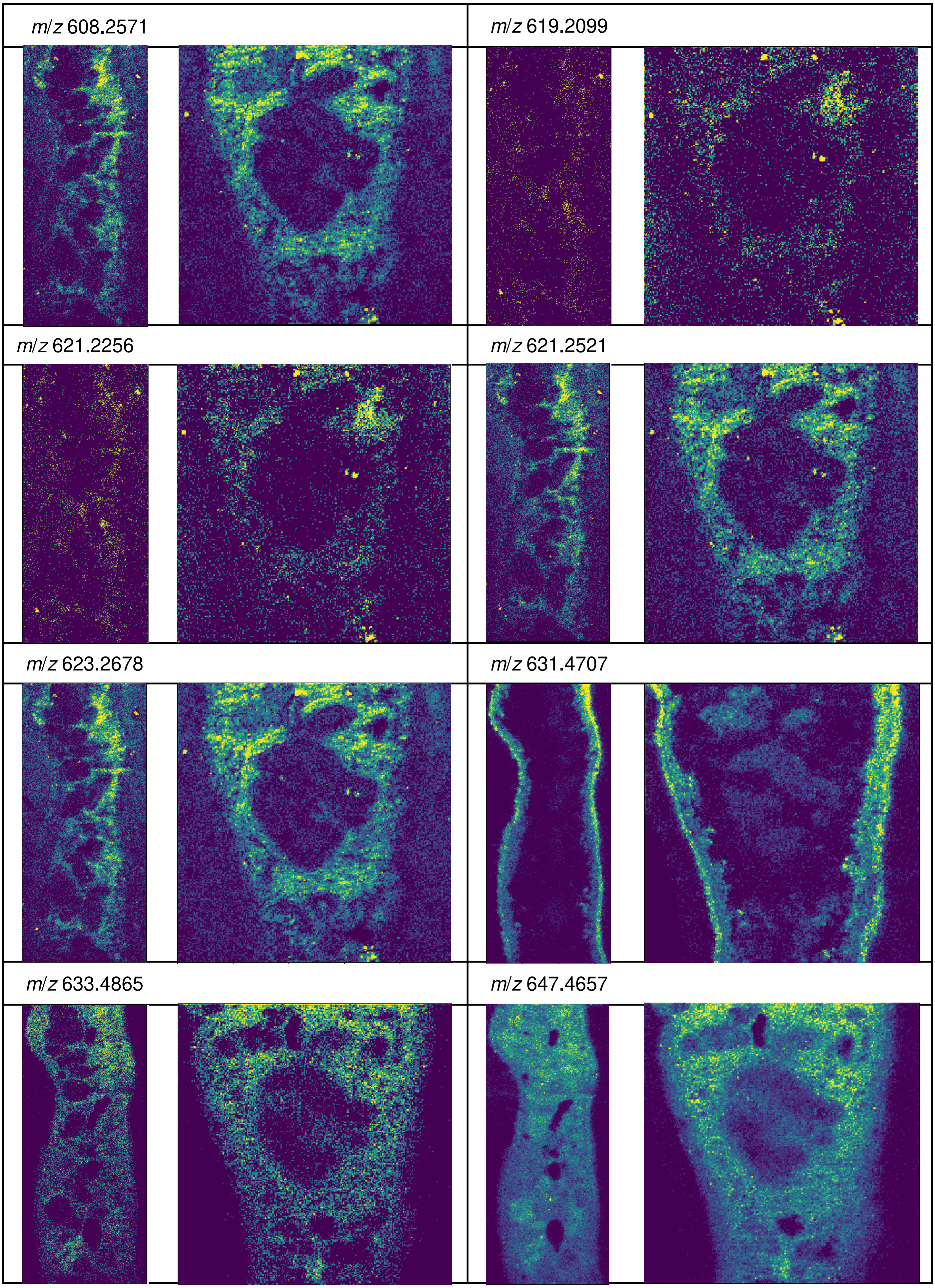
m/z 608.2571
m/z 619.2099
m/z 621.2256
m/z 621.2521
m/z 623.2678
m/z 631.4707
m/z 633.4865
m/z 647.4657

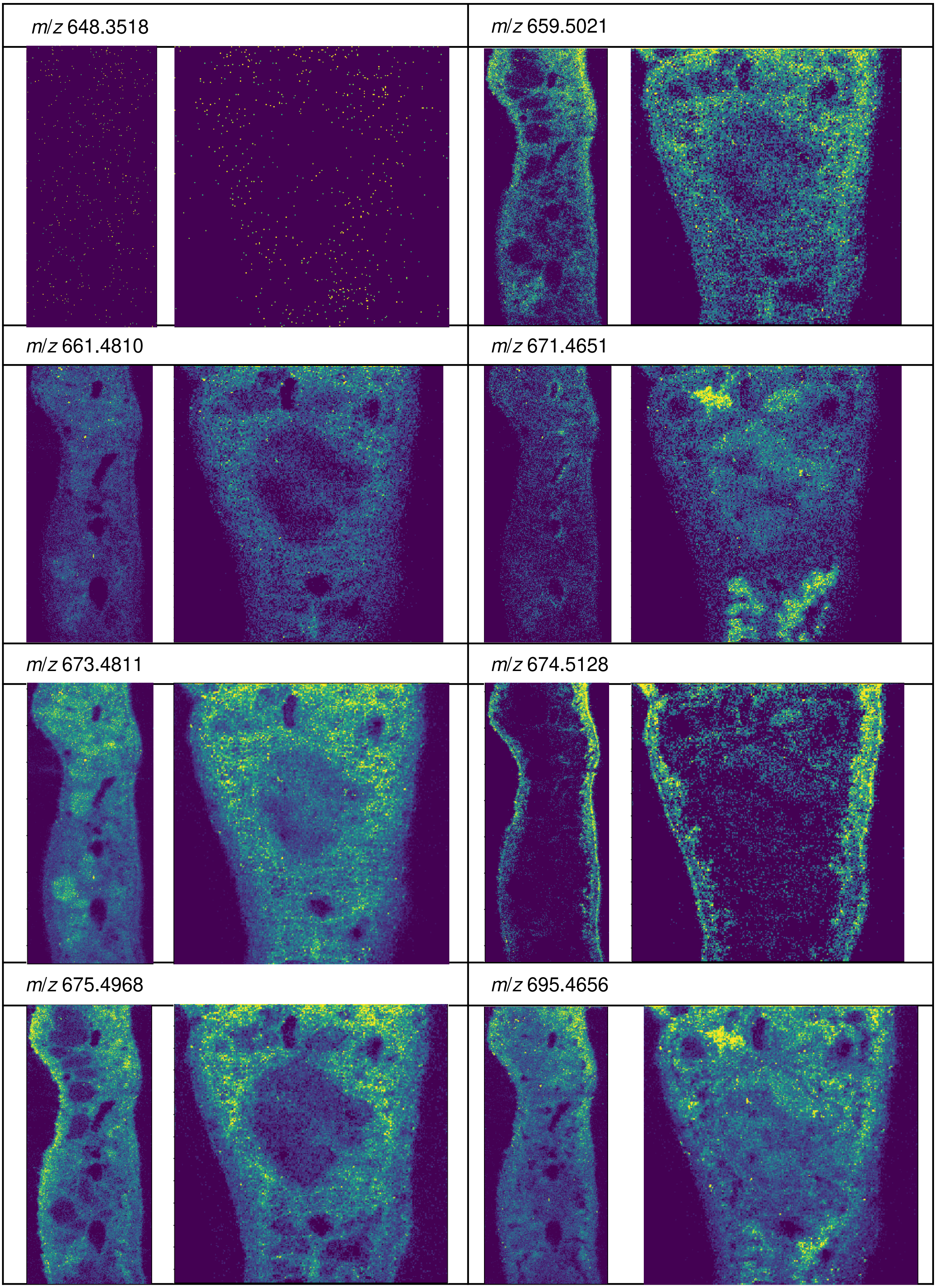
m/z 648.3518
m/z 659.5021
m/z 661.4810
m/z 671.4651
m/z 673.4811
m/z 674.5128
m/z 675.4968
m/z 695.4656

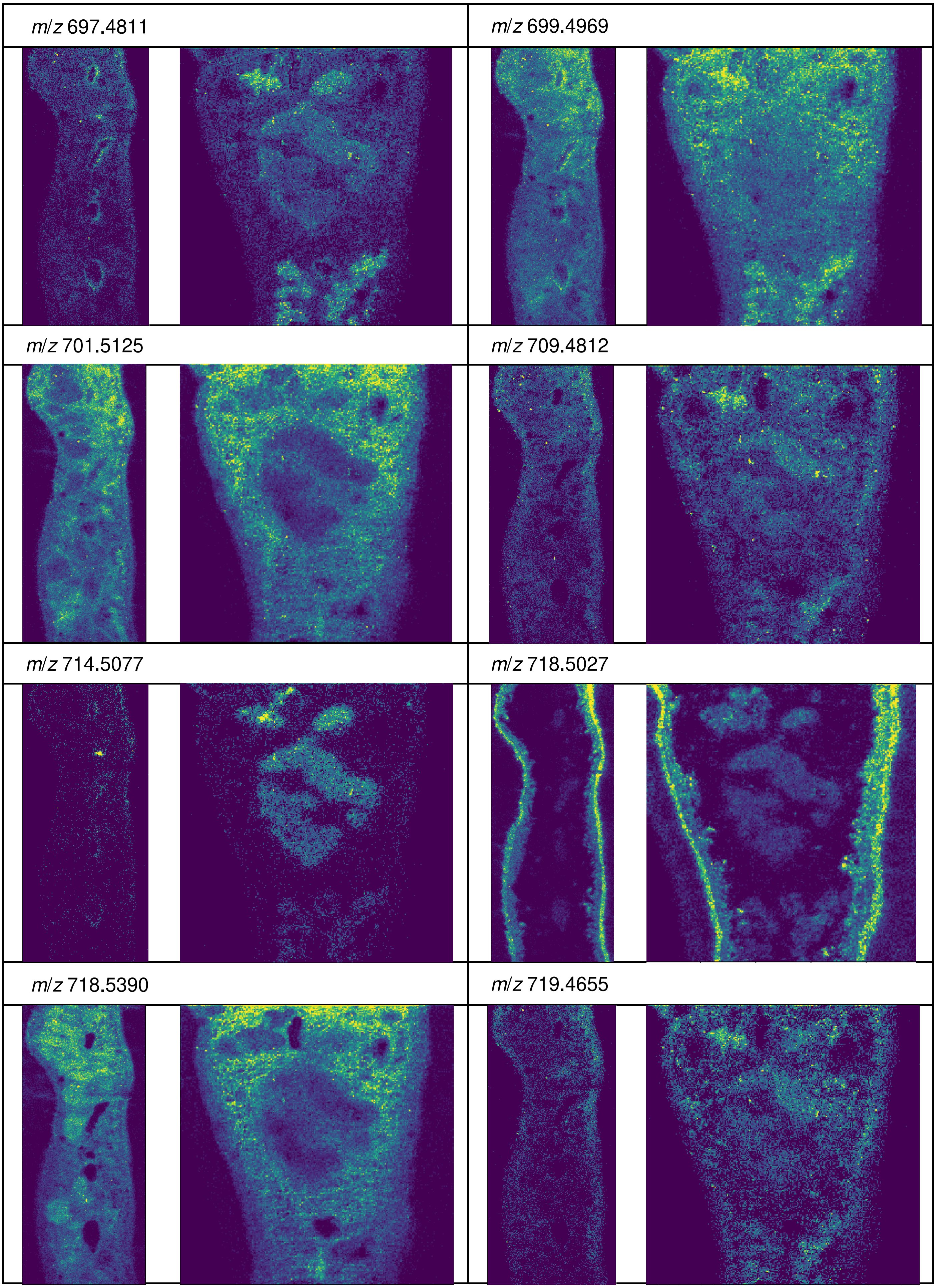

m/z 697.4811
m/z 699.4969
m/z 701.5125
m/z 709.4812
m/z 714.5077
m/z 718.5027
m/z 718.5390
m/z 719.4655

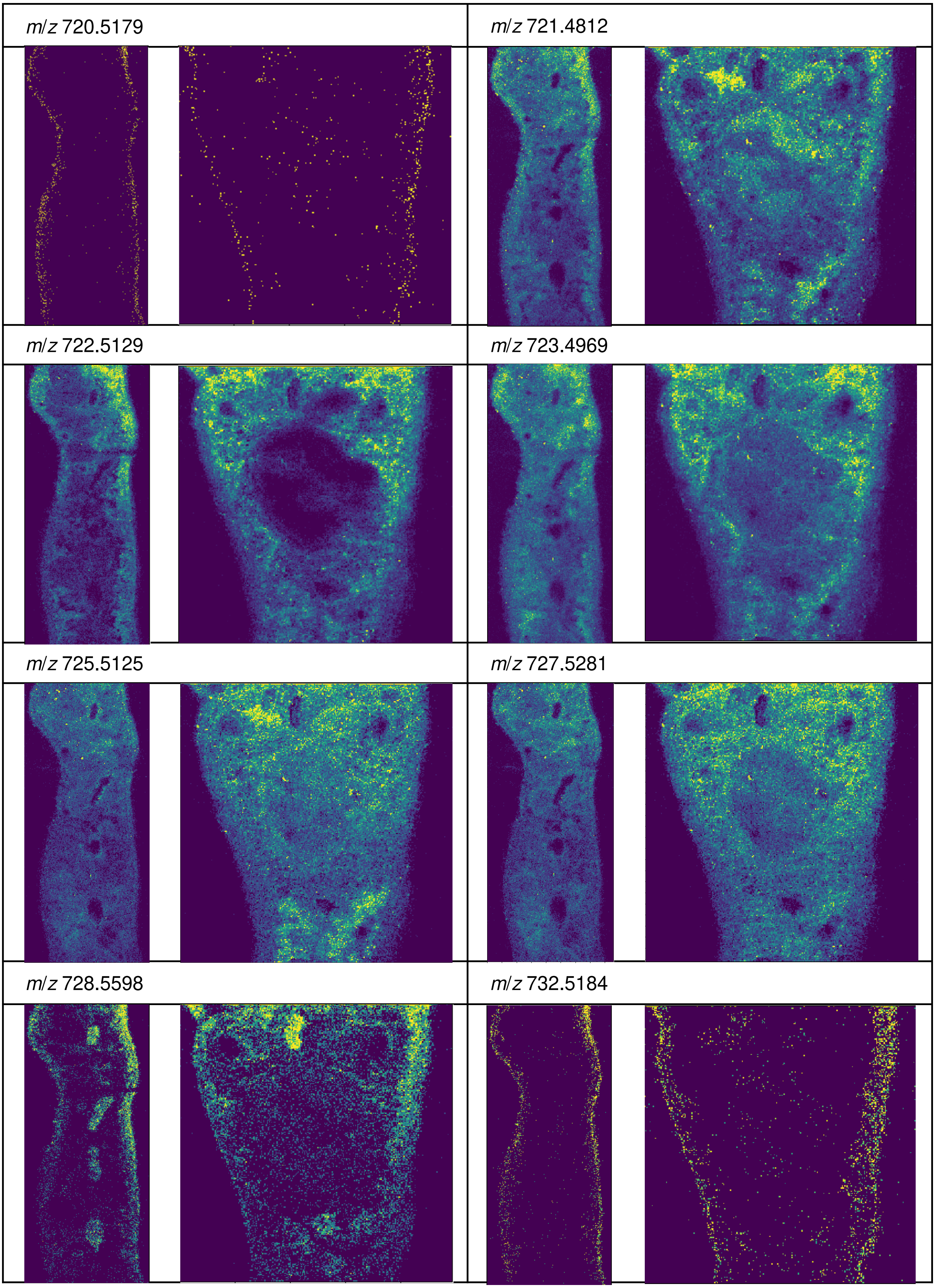
m/z 720.5179
m/z 721.4812
m/z 722.5129
m/z 723.4969
m/z 725.5125
m/z 727.5281
m/z 728.5598
m/z 732.5184

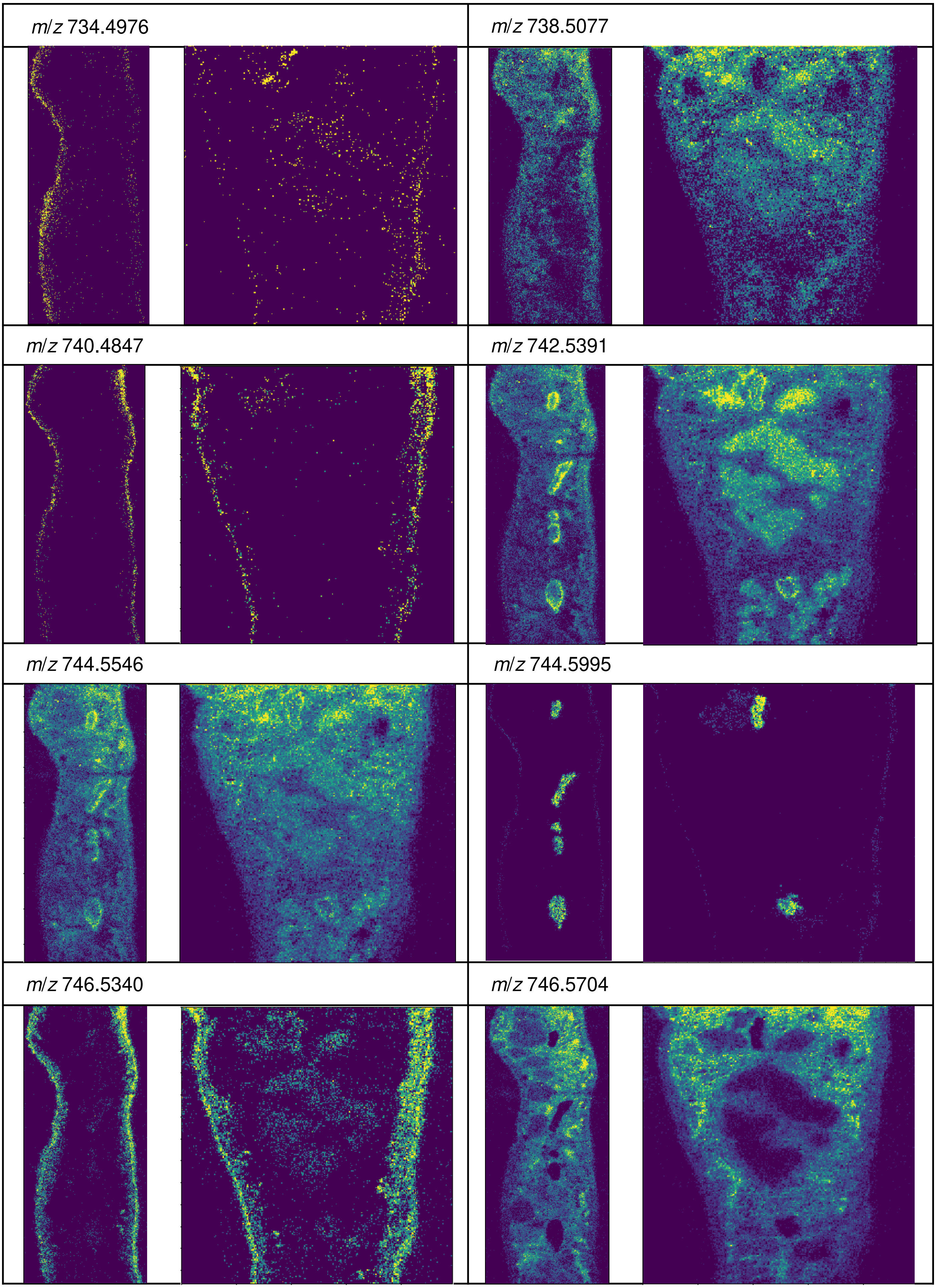

m/z 734.4976
m/z 738.5077
m/z 740.4847
m/z 742.5391
m/z 744.5546
m/z 744.5995
m/z 746.5340
m/z 746.5704

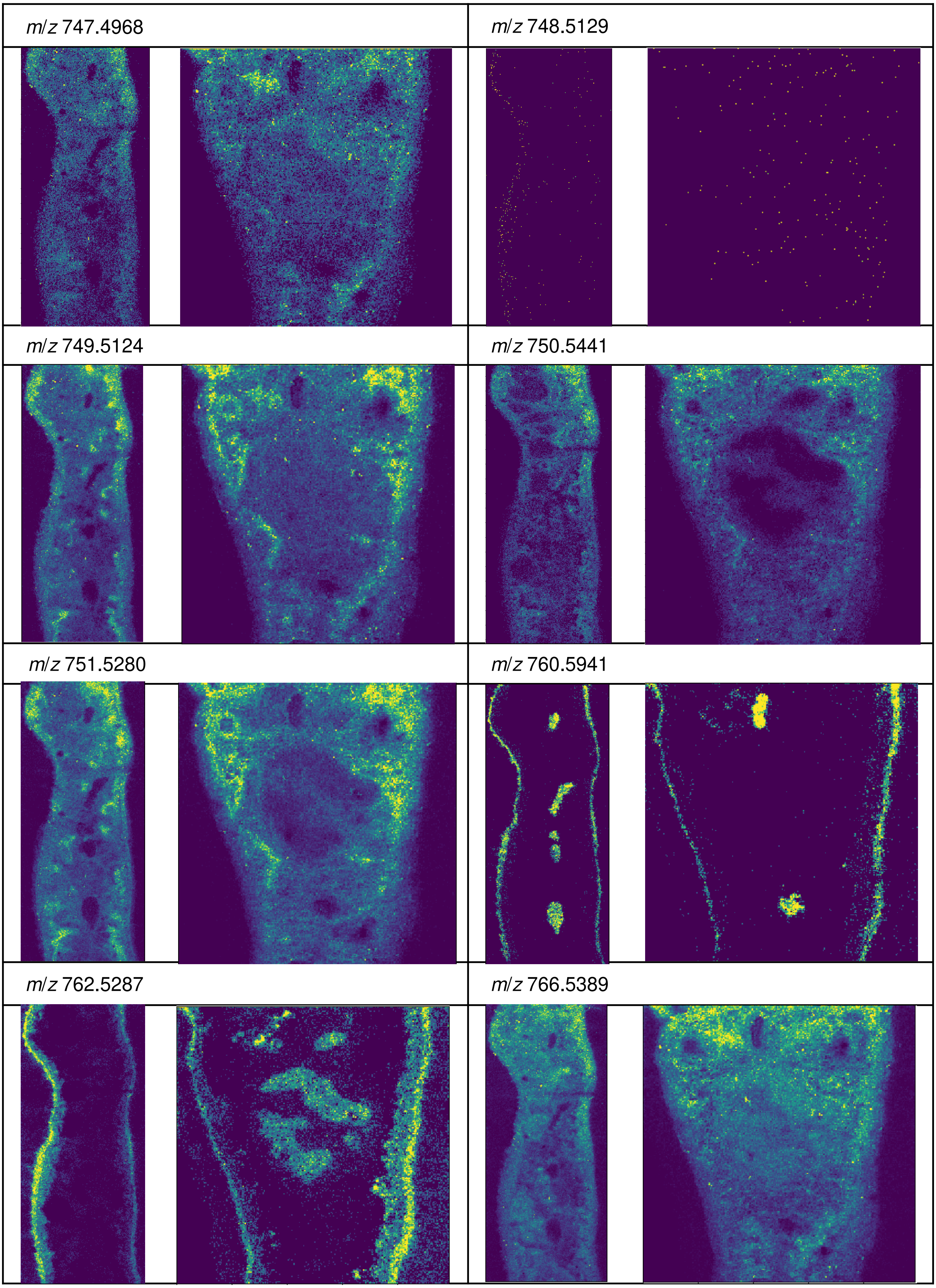
m/z 747.4968
m/z 748.5129
m/z 749.5124
m/z 750.5441
m/z 751.5280
m/z 760.5941
m/z 762.5287
m/z 766.5389

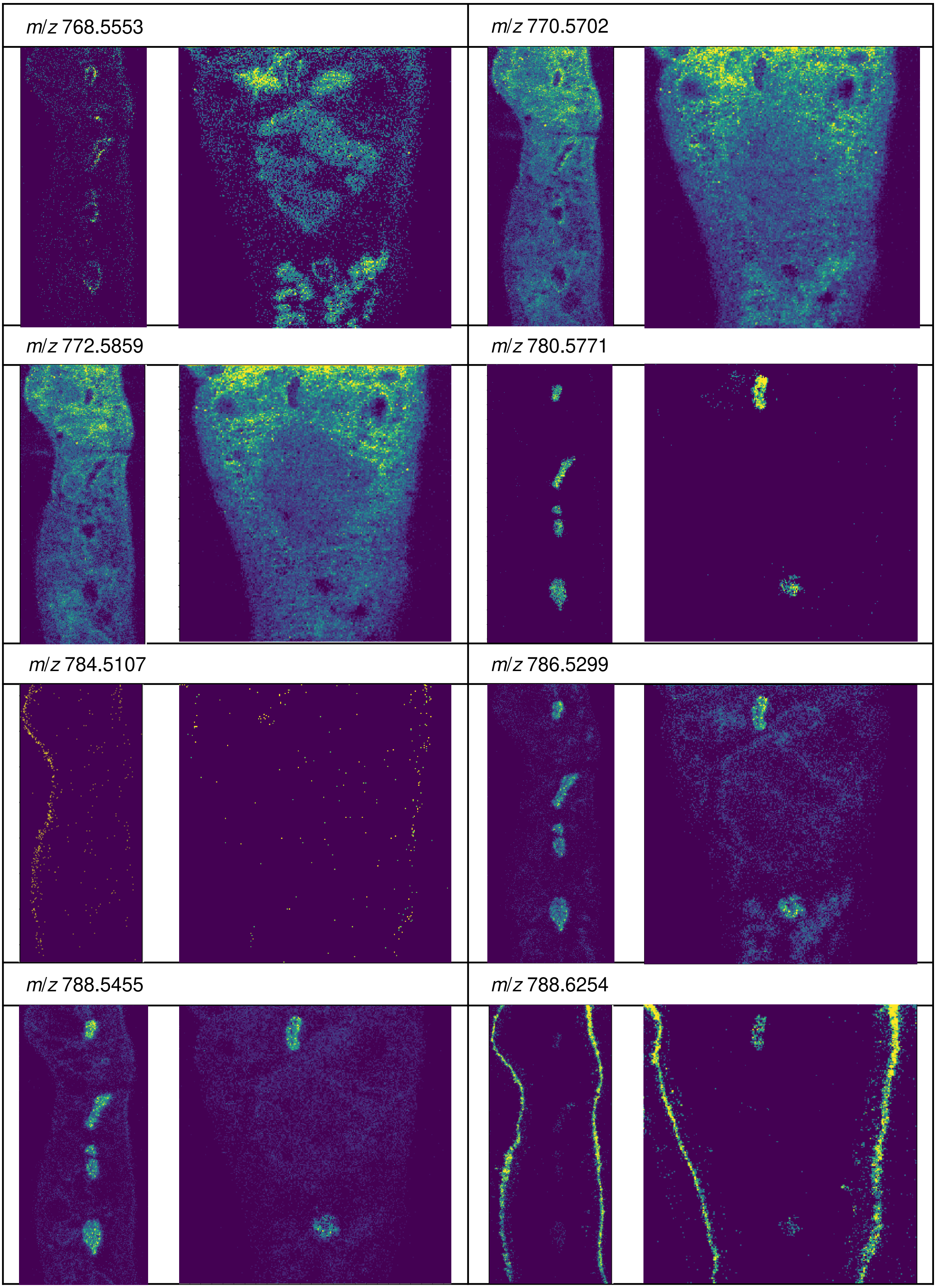
m/z 768.5553
m/z 770.5702
m/z 772.5859
m/z 780.5771
m/z 784.5107
m/z 786.5299
m/z 788.5455
m/z 788.6254

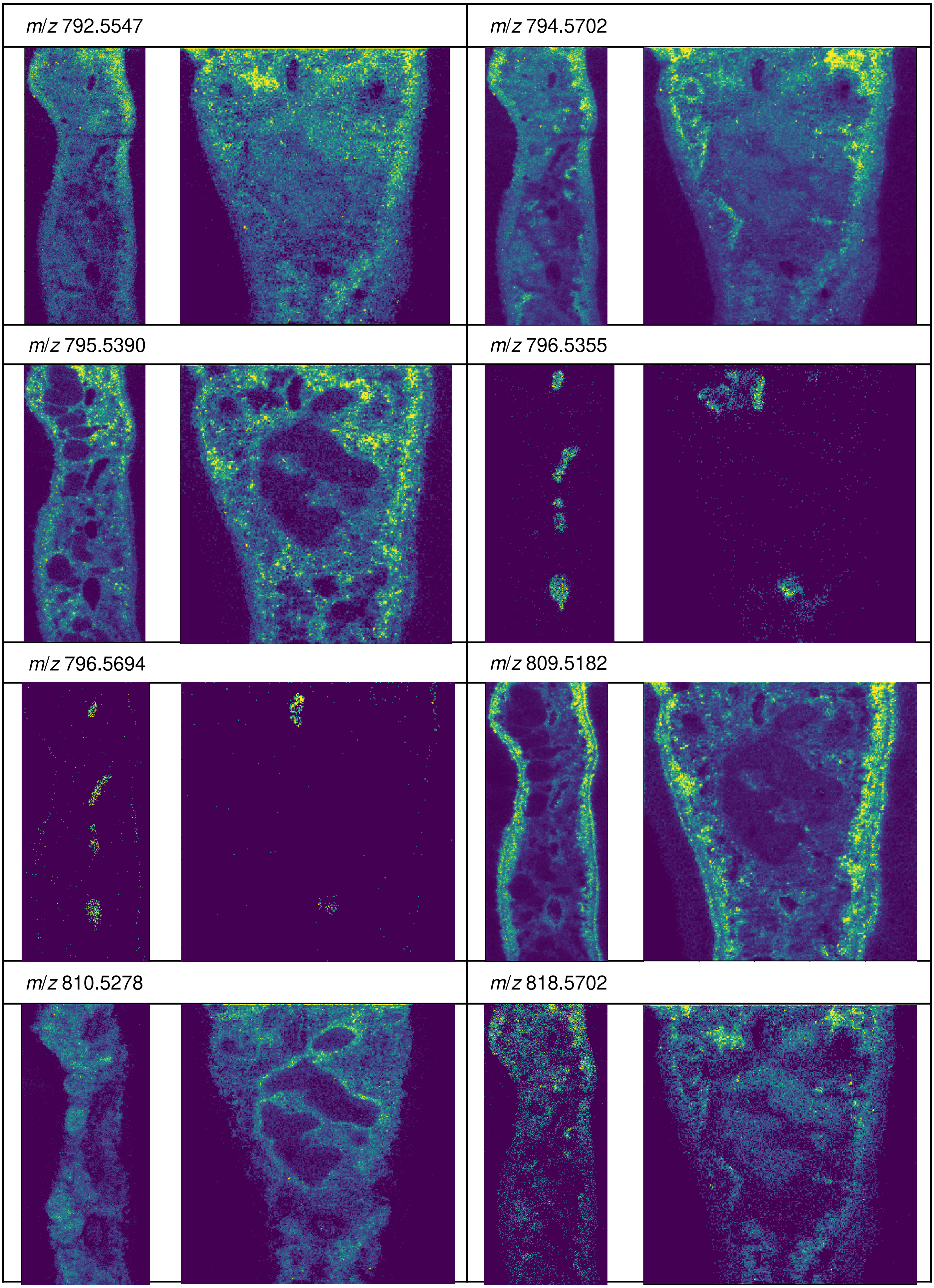
m/z 792.5547
m/z 794.5702
m/z 795.5390
m/z 796.5355
m/z 796.5694
m/z 809.5182
m/z 810.5278
m/z 818.5702

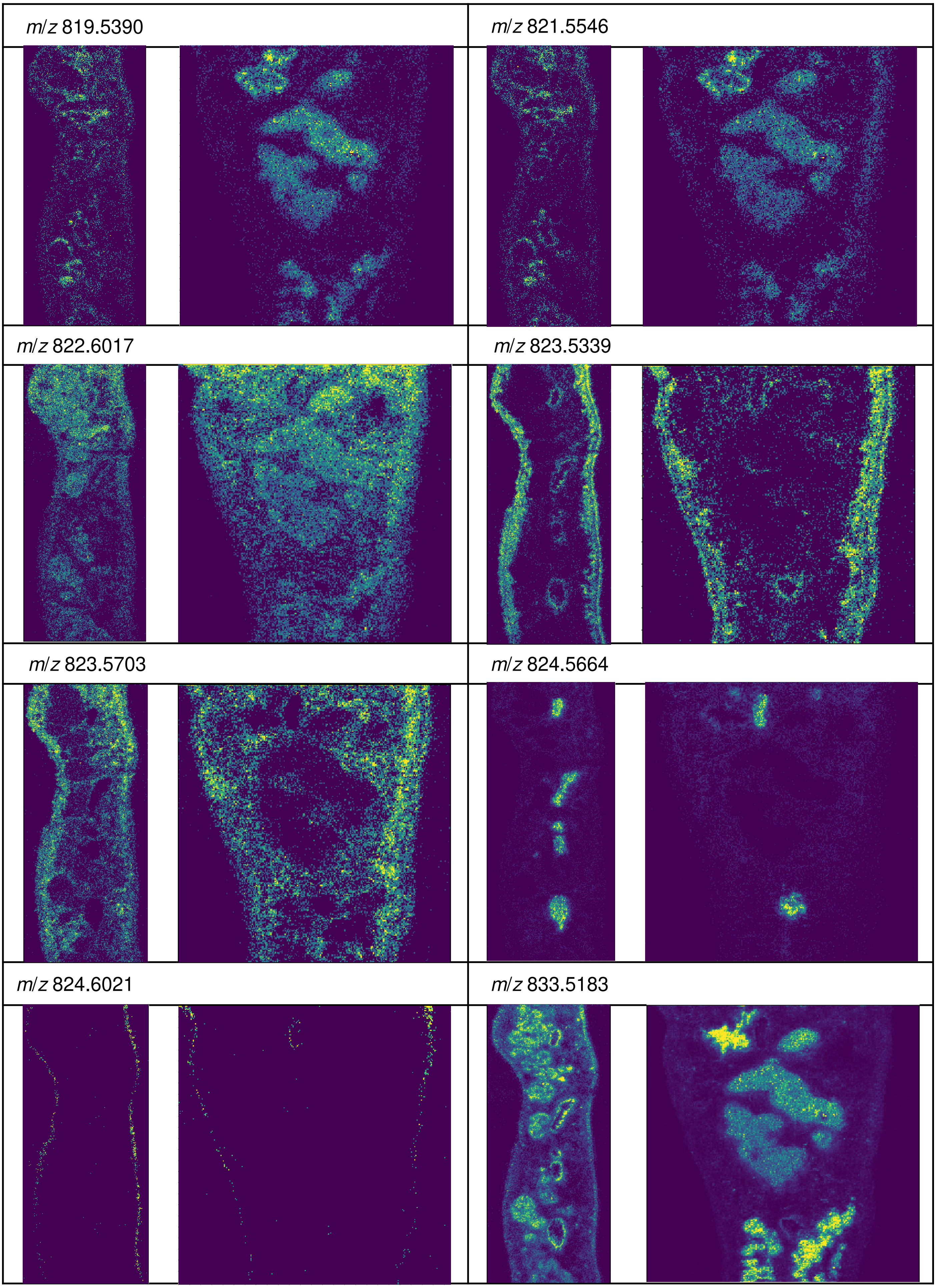
m/z 819.5390
m/z 821.5546
m/z 822.6017
m/z 823.5339
m/z 823.5703
m/z 824.5664
m/z 824.6021
m/z 833.5183

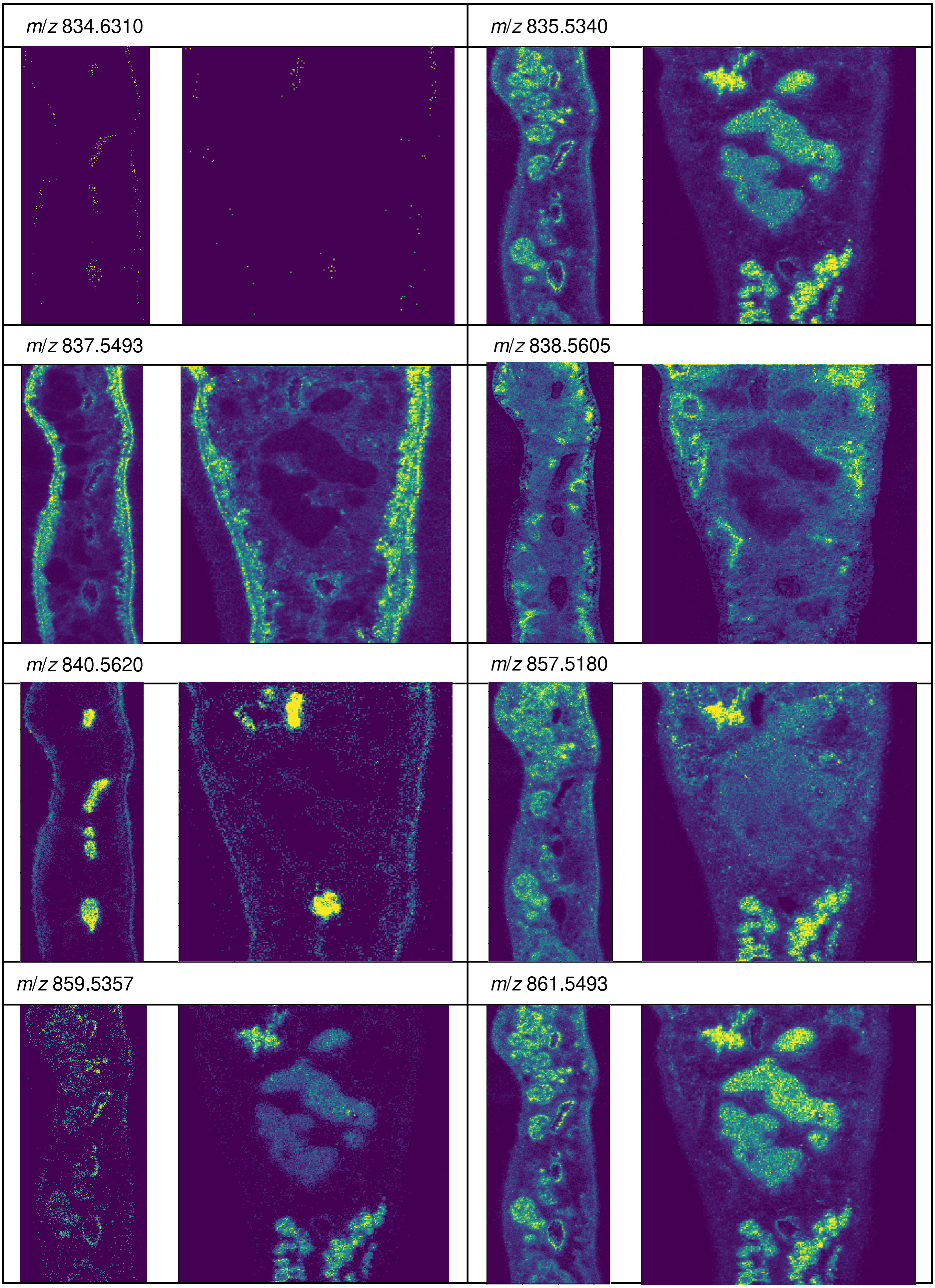

m/*z* 834.6310
m/*z* 835.5340
m/*z* 837.5493
m/*z* 838.5605
m/*z* 840.5620
m/*z* 857.5180
m/*z* 859.5357
m/*z* 861.5493

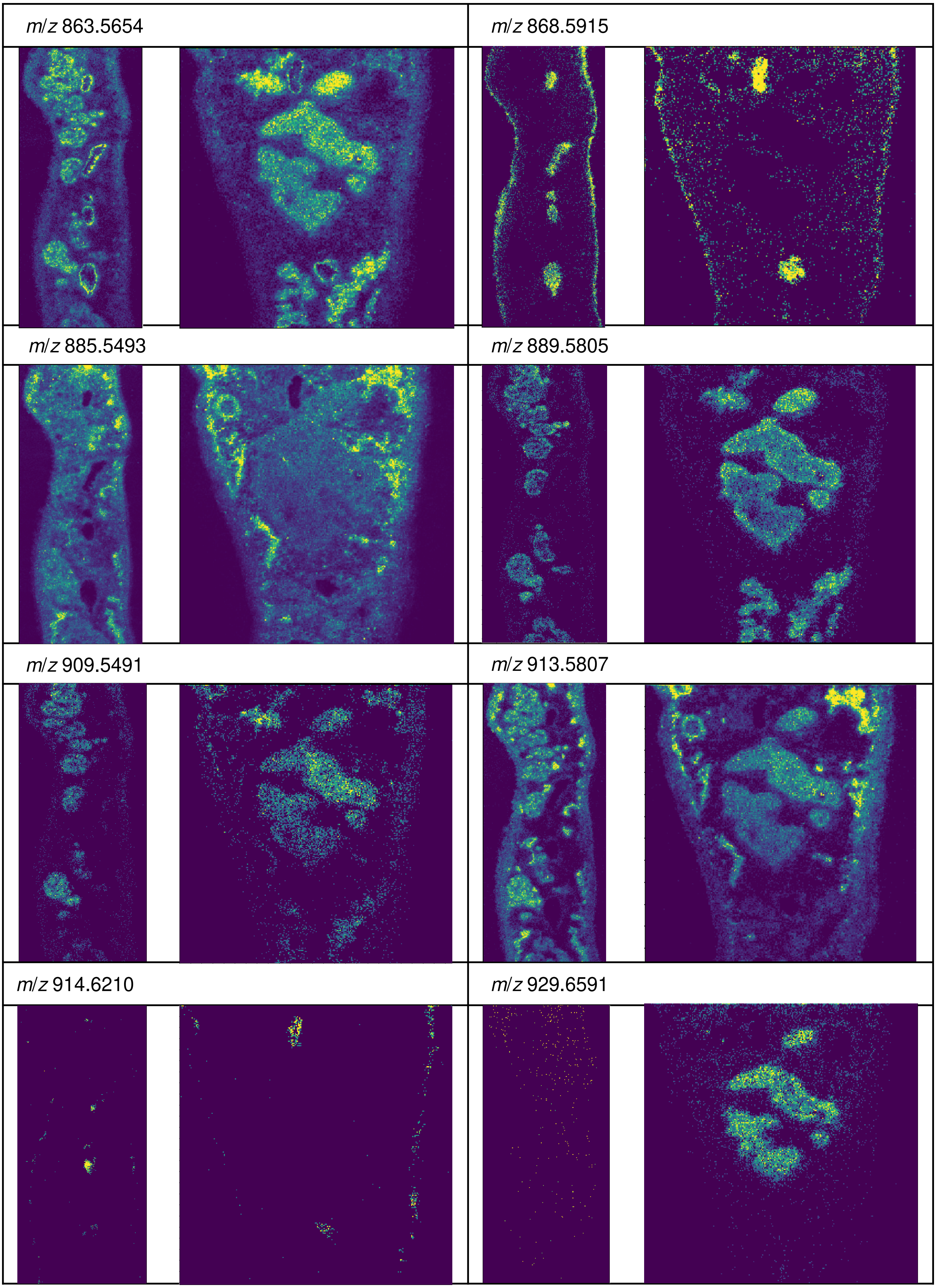

m/z 863.5654
m/z 868.5915
m/z 885.5493
m/z 889.5805
m/z 909.5491
m/z 913.5807
m/z 914.6210
m/z 929.6591

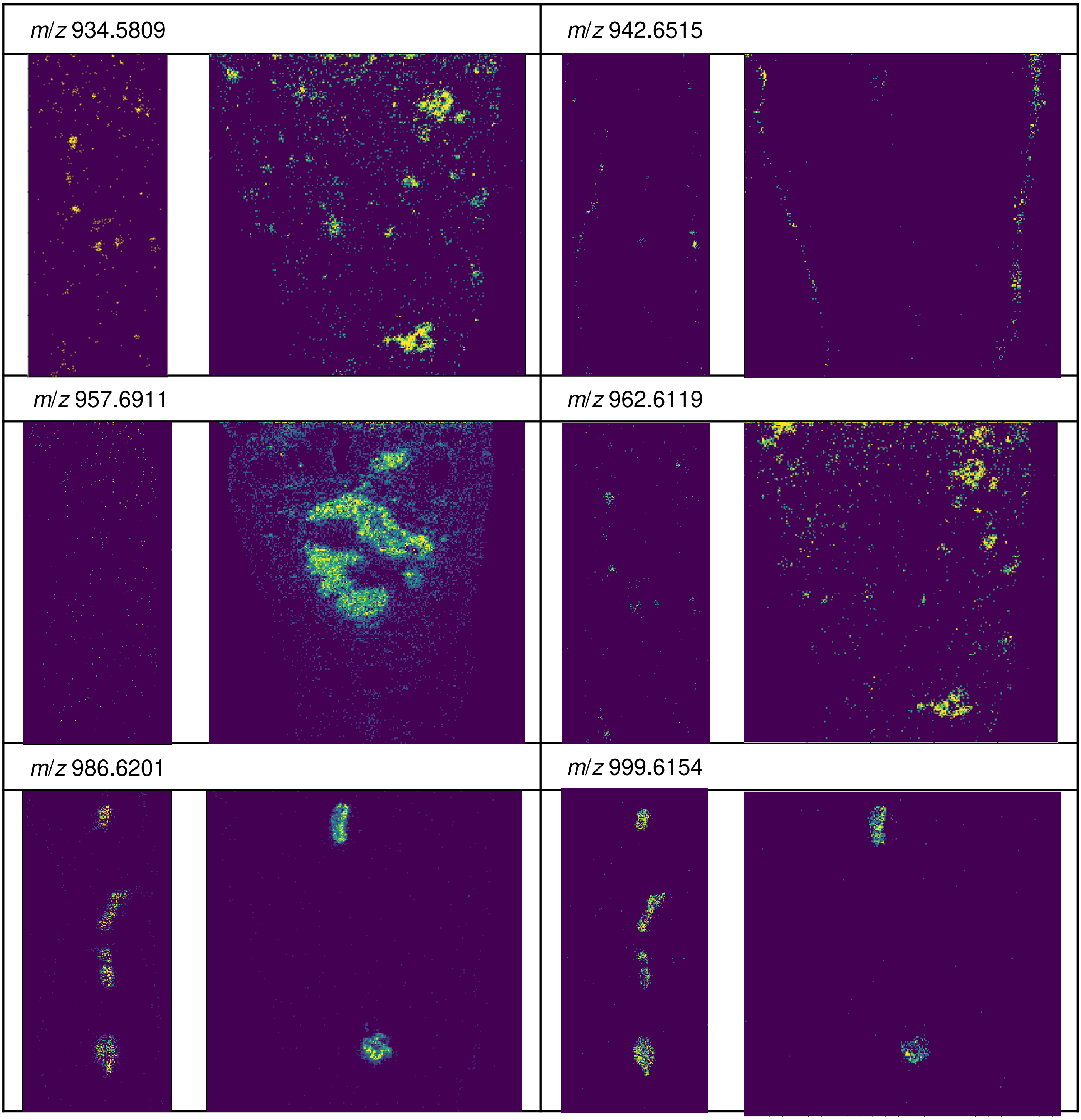

m/*z* 934.5809
m/*z* 942.6515
m/*z* 957.6911
m/*z* 962.6119
m/*z* 986.6201
m/*z* 999.6154
