# Supplementary material for: Spatial lipidomics of Fasciola hepatica using high-resolution AP-SMALDI Orbitrap MSI – a lipid tissue atlas
Source: Front Cell Infect Microbiol. 2026 Oct 22;16:1934046. doi: 10.3389/fcimb.2026.1934046 (PMC13735547; doi:10.3389/fcimb.2026.1934046)
Supplement: Supplementary file 1 [file DataSheet1.zip › Supplementary information.docx]

**Supplementary information**

**Suppl. Tab. 1**: Chemicals used for the experiments with their purities and manufacturers.

| Chemical | Quality grade | manufacturer |
| --- | --- | --- |
| gelatin powder | Ph. Eur. | VWR International, Leuven, Belgium |
| DHB | for synthesis | Merck KGaA, Darmstadt, Germany |
| DAN | 97% | Acros Organics, Geel, Belgium |
| acetone | uvasol | Merck KGaA, Darmstadt, Germany |
| H_2_O | HiPerSolv | VWR International, Rosny-sous-Bois-cedex, France |
| MeOH | HiPerSolv | VWR International, Leuven, Belgium |
| TFA | uvasol | Merck KGaA, Darmstadt, Germany |
| ammonium acetate | for LC-MS | Sigma Aldrich, Steinheim, Germany |
| MtBE | for HPLC | Merck KGaA, Darmstadt, Germany |
| ACN | LC-MS grade | VWR International |
| isopropanol | LiChroSolv | Merck KGaA, Darmstadt, Germany |
| EtOH | LiChroSolv | Merck KGaA, Darmstadt, Germany |
| ammonium formate | 99% | Acros Organics, Geel, Belgium |
| formic acid | for LC-MS | Merck KGaA, Darmstadt, Germany |
| Mayer’s Hematoxylin | - | Sigma Aldrich, Steinheim, Germany |
| Eosin Y | - | Sigma Aldrich, Steinheim, Germany |
| Eukitt quick hardening medium | - | Sigma Aldrich, Steinheim, Germany |

**Suppl. Tab. 2**: UHPLC separation gradient (positive- and negative-ion mode analyses).

| *t* [min] | Mobile phase A | Mobile Phase B |
| --- | --- | --- |
| 0 | 70 | 30 |
| 2 | 57 | 43 |
| 2.1 | 45 | 55 |
| 12 | 35 | 65 |
| 18 | 15 | 85 |
| 20 | 0 | 100 |
| 25 | 0 | 100 |
| 25.1 | 70 | 30 |
| 28 | 70 | 30 |

**Suppl. Tab. 3:** HESI source parameters for LC-MS/MS measurements.

| Parameter | Positive-ion mode | Negative-ion mode |
| --- | --- | --- |
| Sheath gas [a.u.] | 40 | 45 |
| Auxiliary gas [a.u.] | 15 | 12 |
| Sweep gas [a.u.] | 2 | 1 |
| Spray voltage [kV] | 3.5 | −3.5 |
| Capillary *T* [°C] | 300 | 320 |
| S-lens RF | 50 | 55 |
| Auxiliary heater *T* [°C] | 300 | 320 |

**Suppl. Tab. 4:** MS parameters for LC-MS (LC-MS/MS).

| Parameter | Positive-ion mode | Negative-ion mode |
| --- | --- | --- |
| Run time [min] | 28 | 28 |
| Charge state | 1 | 1 |
| Exclusion list | on | on |
| Inclusion list | on | on |
| Resolution | 60 k (45 k) | 60 k (45 k) |
| AGC target | 10^6^ (10^5^) | 10^6^ (10^5^) |
| Maximum injection time | 250 (75) | 250 (75) |
| Scan range | 200-1800 | 200-1800 |
| Top N | 15 | 15 |
| Isolation window (*m*/*z*) | 1 | 1 |
| Stepped NCE | 25, 30 | 20, 30, 40 |
| Intensity threshold | 8∙10^3^ | 8∙10^3^ |
| Exclude isotopes | on | on |
| Dynamic exclusion /s | 8 | 8 |

**Suppl. Tab. 5:** Settings for exclusion list generation in MS-DIAL used in positive- (negative-ion mode).

| Parameter | Setting |
| --- | --- |
| Data type (MS1) | Profile data |
| MS1 tolerance | 0.005 Da |
| MS1 mass range | 0-2000 Da |
| Number of threads | 8 |
| Min. peak height | 50000 amplitude (10000 amplitude) |
| Mass slice width | 0.4 Da |
| Sigma window value | 0.5 |
| Keep isotopic ions until 5 Da | yes |

**Suppl. Tab. 6:** Set parameters for LIPIDMAPS database search.

| Parameter | Positive-ion mode | Negative-ion mode |
| --- | --- | --- |
| Adduct ions | [M+H]^+^, [M+H−H_2_O]^+^, [M+Na]^+^, [M+K]^+^, [M+Na−H]^+^ | [M−H]^−^, [M+Cl]^−^ |
| Mass tolerance | +/− 0.005 Da | +/− 0.01 Da |

**Suppl. Tab. 7:** Overview on the number of reproducibly detected signals with decimals between 0.2 and 0.8, the fraction of these that had hit(s) in the LIPIDMAPS database (within reasonable mass deviation), and the fraction that was confirmed in its bulk identity by (LC-) MS/MS. The numbers are given for each organ and refer to the measurements in positive-ion mode. ^13^C isotopologue signals were removed before the calculation.

| Organ | # Repr. detected signals | # Signals with DB hit(s) | # Signals with MS/MS-confirmed identity |
| --- | --- | --- | --- |
| Tegument | 217 | 160 (74%) | 118 (74%) |
| Ovary | 208 | 148 (71%) | 123 (83%) |
| Intestine | 246 | 147 (60%) | 120 (82%) |
| Testis | 198 | 139 (70%) | 112 (81%) |

**Suppl. Tab. 8:** Overview on the number of reproducibly detected signals with decimals between 0.2 and 0.8, the fraction of these that had hit(s) in the LIPIDMAPS database (within reasonable mass deviation), and the fraction that was confirmed in its bulk identity by (LC-) MS/MS. The numbers are given for each organ and refer to the measurements in negative-ion mode. ^13^C isotopologue signals were removed before the calculation.

| Organ | # Repr. detected signals | # Signals with DB hit(s) | # Signals with MS/MS-confirmed identity |
| --- | --- | --- | --- |
| Tegument | 117 | 89 (76%) | 52 (58%) |
| Ovary | 74 | 69 (93%) | 48 (70%) |
| Intestine | 49 | 45 (92%) | 32(71%) |
| Testis | 39 | 38 (97%) | 28 (74%) |

**Suppl. Fig. 1:** HCD fragment spectrum (average of 4 single spectra) of the precursor *m*/*z* 566.55. The full spectrum shows two fragments originating from water loss commonly observed for ceramides. In the zoom-in, fragment signals from N^II^ type ceramide fragmentation were observed. Here, not only the fragments from doubly hydroxylated Cer species are present („d“ in green) but also those from triply hydroxylated ones („t“ in blue). Together with the FA residue fragments (in orange), this proofs the presence of both, Cer 36:1;O2 and Cer 36:0;O3 in the sample. Chain lengths other than 18, 16 and 20 were also found but not highlighted here.

**Suppl. Fig. 2:** Scores plots (as shown in **Fig. 2**) with corresponding loading plots (three different perspectives each) from positive- and negative-ion mode analyses. Colors in the score plots indicate ROIs of tegument (green), intestine (yellow), ovary (red), and testis (blue).
